# Complete genome sequence of hypervirulent and outbreak-associated *Acinetobacter baumannii* strain LAC-4: epidemiology, resistance genetic determinants and potential virulence factors

**DOI:** 10.1038/srep08643

**Published:** 2015-03-02

**Authors:** Hong-Yu Ou, Shan N. Kuang, Xinyi He, Brenda M. Molgora, Peter J. Ewing, Zixin Deng, Melanie Osby, Wangxue Chen, H. Howard Xu

**Affiliations:** 1 State Key Laboratory of Microbial Metabolism and School of Life Sciences & Biotechnology, Shanghai Jiaotong University, Shanghai, China; 2 Department of Biological Sciences, California State University Los Angeles, Los Angeles, California, USA; 3 Department of Pathology, LAC+USC Medical Center, Los Angeles, California, USA; 4 Human Health Therapeutics, National Research Council Canada, 100 Sussex Drive, Ottawa, Ontario K1A 0R6, Canada

## Abstract

*Acinetobacter baumannii* is an important human pathogen due to its multi-drug resistance. In this study, the genome of an ST10 outbreak *A. baumannii* isolate LAC-4 was completely sequenced to better understand its epidemiology, antibiotic resistance genetic determinants and potential virulence factors. Compared with 20 other complete genomes of *A. baumannii*, LAC-4 genome harbors at least 12 copies of five distinct insertion sequences. It contains 12 and 14 copies of two novel IS elements, IS*Aba25* and IS*Aba26*, respectively. Additionally, three novel composite transposons were identified: Tn*6250*, Tn*6251* and Tn*6252*, two of which contain resistance genes. The antibiotic resistance genetic determinants on the LAC-4 genome correlate well with observed antimicrobial susceptibility patterns. Moreover, twelve genomic islands (GI) were identified in LAC-4 genome. Among them, the 33.4-kb GI12 contains a large number of genes which constitute the K (capsule) locus. LAC-4 harbors several unique putative virulence factor loci. Furthermore, LAC-4 and all 19 other outbreak isolates were found to harbor a heme oxygenase gene (*hemO*)-containing gene cluster. The sequencing of the first complete genome of an ST10 *A. baumannii* clinical strain should accelerate our understanding of the epidemiology, mechanisms of resistance and virulence of *A. baumannii*.

Recognized as one of the most problematic bacterial pathogens due to emergence of multidrug-resistant (MDR) strains^1^, *Acinetobacter baumannii* has been responsible for a significant proportion of world-wide healthcare-acquired infections, including ventilator-associated pneumonia, surgical site and urinary tract infections and septicemia^2^,^3^,^4^,^5^. Additionally, MDR clinical isolates of this species have been found to infect military personnel wounded in combat zones^6^,^7^.

Hospital outbreaks of *A. baumannii* infection are often associated with multidrug resistance in the causative strains^8^,^9^. Besides their intrinsic resistance to certain antibiotics due to the presence of native β-lactamase genes, poor permeability and efflux systems, *A. baumannii* strains have acquired a large array of antibiotic resistance mutations and genes, located either on the chromosome or plasmids^10^,^11^. Furthermore, clinical strains of *A. baumannii* resistant to carbapenems, the last resort antibiotics to treat infections caused by *A. baumannii*, have been found to possess acquired *bla* genes encoding several groups of carbapenem-hydrolyzing β-lactamases, such as *bla*_OXA-23_^12^,^13^, *bla*_OXA-24/33/40_^14^,^15^, and *bla*_OXA-58_^16^. These *bla* genes appear to have been transferred via mobile genetic elements such as insertion sequences, transposons or plasmids^12^,^13^,^15^,^16^.

Despite the clinical significance of *A. baumannii* infections, the molecular basis on the virulence and acquisition of multidrug resistance by *A. baumannii* remains largely unknown. To better understand the genome plasticity, natural history, epidemiology and acquisition of resistance and pathogenicity islands/genes of *A. baumannii*, the complete genomes of 21 *A. baumannii* strains, including LAC-4, became available as of December 31, 2014^17^,^18^,^19^,^20^,^21^,^22^,^23^,^24^,^25^,^26^,^27^,^28^,^29^,^30^. In addition, the genomes of hundreds of *A. baumannii* strains have been sequenced to scaffold or contig levels (http://www.ncbi.nlm.nih.gov/genome/genomes/403). Most of these sequenced *A. baumannii* genomes are divided into 31 groups on basis of their sequence similarity. These efforts and other incomplete Whole Genome Shotgun (WGS) projects involving *A. baumannii* strains and strains of other *Acinetobacter* species^31^,^32^ will likely offer additional insights on epidemiology, phylogenetics, evolution of pathogenic strains and gene flows among *Acinetobacter* species including *A. baumannii*.

Several years ago, we described antibiotic resistance patterns and clonal relationships of 20 MDR clinical isolates of *A. baumannii* obtained from nosocomial outbreaks in Los Angeles County hospitals (LAC-1 to LAC-20)^5^. Our pulsed-field gel electrophoresis (PFGE) fingerprinting analysis indicated that these isolates appeared to have originated from eight epidemiologically distinct lineages^5^. More significantly, we identified the LAC-4 strain as hypervirulent in a mouse model of intranasal infection^33^ in comparison to other clinical isolates and laboratory strains of *A. baumannii*, including the eight representative LAC isolates and the widely studied clinical strain AYE^22^,^34^. The LAC-4 strain reliably reproduces the most relevant features of human pulmonary *A. baumannii* infection, including significant extrapulmonary dissemination and bacteremia^33^. Subsequent studies showed that LAC-4 exhibits high serum resistance, expresses a highly efficient heme utilization system^35^ and contains some unique structure and composition in its surface polysaccharide^36^, which may contribute to its hypervirulence. However, the precise mechanism of the hypervirulence of LAC-4 remains to be determined. Here we describe the complete genome sequence of the LAC-4 strain with special emphasis on the comparative genomics analyses to identify genomic regions that may contribute to the acquisition of antibiotic resistance and establishment of superior colonization and invasion by this hypervirulent strain.

## Results and discussion

### Phylogenetic lineages based on trilocus multiplex PCR and MLST

To understand the epidemiology and phylogenetics of 20 clinical isolates of *A. baumannii* (including LAC-4) obtained from four apparent nosocomial outbreaks, we first attempted to determine the clonal relationships among these isolates of *A. baumannii* by Trilocus multiplex PCR (TLM-PCR) analyses. Our results indicate that we can only type four isolates (LAC-11, LAC-12, LAC-13 and LAC-14) belonging to Global Clone (GC) II (Table 1). Since the TLM-PCR method failed to resolve phylogenetic relationships of most of these *A. baumannii* isolates, multilocus sequence typing (MLST) scheme based on Pasteur Institute approach was subsequently employed. Our results showed that previously non-typable isolates belong to unusual ST types (such as ST10, ST241 and ST417) (Table 1). Previously, PFGE profiling divided these 20 outbreak isolates into eight distinct clonal groups: LAC-1 to LAC-3; LAC-4; LAC-5 and LAC-8; LAC-6; LAC-7, LAC-9, and LAC-10; LAC-11 to LAC-14; LAC-15; and LAC-16 to LAC-20^5^. In accordance with the PFGE grouping^5^, MLST typed LAC-5 and LAC-8 to a rare ST241 (Table 1). Since these two strains were isolated during two separate outbreaks in a single hospital, it appears that the ST241 lineage persisted for at least four years (1997–2001) in the same facility. More interestingly, we found that LAC-4, which was much more virulent than other LAC isolates in mice^33^, belongs to ST10 (Table 1). Most importantly, two series of outbreaks were caused by ST10 strains in two separate hospitals (LAC-1 to LAC-4 in Hospital A; LAC-16 to LAC-20 in Hospital C) in Los Angeles County, California, during the late 1990s (Table 1), suggesting that ST10 strains were quite dominant in causing nosocomial outbreaks in Los Angeles County at the time, with LAC-4 being their representative. While LAC-1 to LAC-4 were all typed to ST10, the PFGE profile of LAC-4 diverged from those of LAC-1 to LAC-3 sufficiently to be grouped as a separate clone^5^, indicative of possible divergent evolution of LAC-4 from its original clone.

There have been few reports describing clinical isolates of *A. baumannii* belonging to ST10. Among 1237 *A. baumannii* strains with assigned STs in Pasteur Institute's MLST database as of Oct 23 2014 (the most recent update), only three *A. baumannii* ST10 strains were listed. Recently, an ST10 strain of *A. baumannii* was isolated from a wound of a Canadian patient who had been previously hospitalized in India^37^. This strain and a strain of *Klebsiella pneumoniae* were transmitted to five other patients in an Edmonton, Alberta hospital, resulting in the colonization of four patients and the death of one patient due to septic shock caused by the OXA-23-producing *A. baumannii* strain^37^. Furthermore, four of five MDR strains of *A. baumannii* (LAC-6, LAC-7, LAC-9 and LAC-10) isolated during another outbreak in Hospital A in 2001 were typed to an uncommon ST417, indicating ST417 became dominant in this hospital in 2001 (Table 1)^5^. These results show that there was a clonal succession of outbreak strains in that hospital during a five-year span (1996–2001), transitioning from ST10 dominance in 1996–1999 to ST417 dominance in 2001. Previously reported clinical outbreaks were frequently caused by strains belonging to ST1 (Global Clone I)^38^,^39^,^40^, ST2 (Global Clone II)^40^,^41^ or ST15^40^. To our knowledge, in this report we describe the first nosocomial outbreaks caused by unusual ST10 and ST417 *A. baumannii* strains (Table 1).

### LAC-4 genome sequences and general features

Our MLST analysis showed that LAC-4 and eight other outbreak LAC strains belong to the unusual ST10 (Table 1). Previous studies have shown that LAC-4 exhibits several distinctive attributes (iron utilization and unusual repeating unit composition of surface polysaccharides) that may contribute to its hypervirulence in mice^33^,^36^. Since none of the *A. baumannii* strains whose genomes were completely sequenced belongs to ST10, we decided to completely sequence the genome of LAC-4 using a combination of Roche 454 FLX+, Illumina Hiseq2000 and Illumina Miseq platforms, with gaps/uncertainties filled or clarified by genomic PCR and sequencing of PCR products. Our analysis showed that the LAC-4 genome consists of a circular chromosome of 3,954,354 base pairs and two circular plasmids, one with 8,006 base pairs while the other with 6,076 base pairs. The general features of the LAC-4 genome are summarized in Table 2.

**Figure 1:**
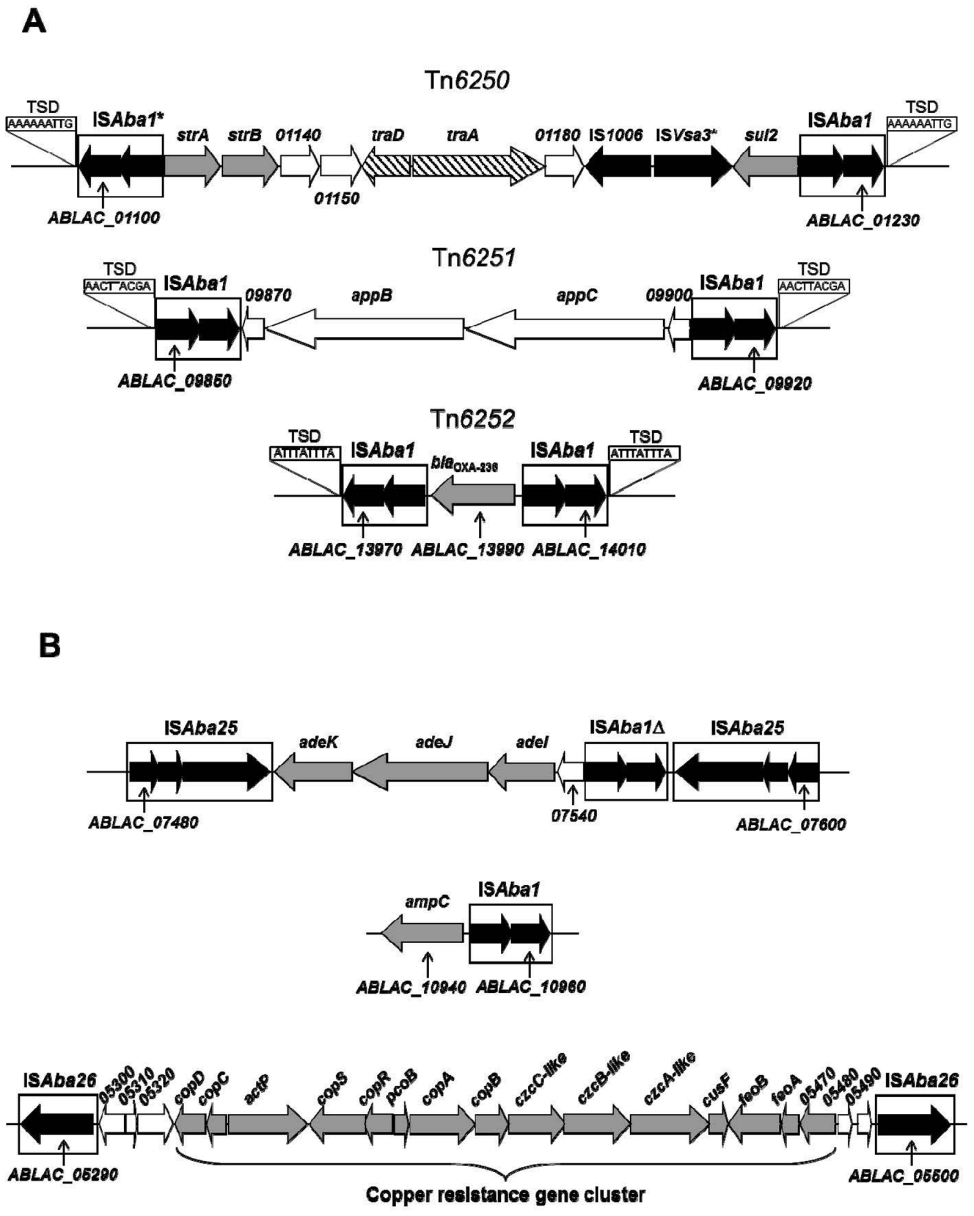
Select examples of insertion sequences and transposons in LAC-4 genome. Direction of transcription of genes is shown by the direction of the arrows. Dark arrows denote ORFs for transposase genes. Gray shaded arrows mark genes potentially conferring resistance to antimicrobial agents or copper. Genes involved in conjugation (*tra* genes) are marked by striped arrows, while genes coding for proteins of unknown or other functions are shown in open arrows. Due to space limitation, certain loci are labeled with abbreviated locus-tags without the full name (e.g., ABLAC_01150 is abbreviated as 01150). Sizes of genes and intergenic distances are not drawn to scale. (A) Composite transposons (Tn*6250*, Tn*6251* and Tn*6252*) formed by IS*Aba1* elements. Target site duplication (TSD) of 9 bases in length was noted only next to the external inverted repeats (facing outwards). An asterisk (*) next to IS*Vsa3* indicates the partial nature of the element (likely not functional). (B) Gene or gene clusters flanked by IS elements. A delta symbol (Δ) next to IS*Aba1* denotes an apparently nonfunctional copy of the element.

The LAC-4 genome contains multiple copies of five types of IS elements: IS*Aba1*, IS*Aba13*, IS*Aba125*, and novel IS*Aba25* and IS*Aba26* whose names were recently assigned (see Table 2 and Supplementary Table S1 online). There are 19 copies of IS*Aba1* in the LAC-4 genome. Three pairs of IS*Aba1* constitute three novel transposons (Tn*6250*, Tn*6251* and Tn*6252*) because identical target site duplication (TSD) sequences are found next to the external (outward facing) inverted repeats (see Fig. 1A; Supplementary Table S1 online), while one IS*Aba1* element is found to be linked to an *ampC* gene (Fig. 1B). In addition, we identified in the LAC-4 genome 12 copies of a novel IS element (IS*Aba25*) whose closest hit from the IS Finder database is IS*Aba16* (see Supplementary Table S1 online). This IS element has 2,491 base pairs in length with three ORFs, while IS*Aba16* has 2,552 bp. Importantly, the largest ORF of IS*Aba25* has a 63-bp deletion compared to IS*Aba16*, resulting in a predicted polypeptide of 527 amino acids, 21 amino acids shorter than its counterpart in IS*Aba16* (which has 548 amino acids). Two copies of IS*Aba25* are associated with genes of a RND type of efflux pump system (AdeIJK) (Fig. 1B), probably contributing to LAC-4's resistance to a number of antimicrobial agents. Furthermore, 14 copies of another novel IS element (IS*Aba26*) are located on the LAC-4 chromosome (see Supplementary Table S1 online). This IS is 1,318 bp in length and contains one gene encoding a transposase with 402 amino acids. While transposase protein sequence BLAST analysis indicates IS*Aba26* belongs to the mutator family of transposases, IS Finder database BLASTp search identifies IS*Ec39* as its closest homologue, sharing 72% transposase amino acid identities. IS*Aba26* produces TSD of 8 bases in length (see Supplementary Table S1 online) and its inverted repeats (IRL and IRR) are 26 bp in lengths. Interestingly, two copies of IS*Aba26* flank a large number of continuous loci (15 genes) which are predicted to be involved in copper resistance (Fig. 1B). Moreover, 22 copies of IS*Aba13* are scattered around the chromosome of LAC-4 (see Supplementary Table S1 online). Among these elements, 14 copies were found to create TSD sequences of 9 bases in length as expected, while one element has an 8-base TSD (see Supplementary Table S1 online). Finally, we found 14 IS*Aba125* elements in LAC-4 genome, each with a TSD of 3 bases (see Supplementary Table S1 online).

The LAC-4 genome also includes two plasmids (Table 2). Plasmid pABLAC1 contains nine predicted ORFs (ABLAC_p100010–ABLAC_p100090). Among these loci, ABLAC_p100020 (*rep*) encodes a replicase (Aci3) belonging to group 3 of replicases defined^42^. Fifty-six base pairs upstream of the *rep* gene, four copies of iterons were found with a sequence of 5′- TAAAACGAGGTTTACCTTGCAT-3′, which is identical to those observed in replicons Ab203-Aci3 and Ab736-Aci7^42^. Iterons have been shown to be involved in controlling plasmid replication via interacting with replicase proteins^43^. Additionally, an AT-rich sequence (5′-AAAAATAT-3′) identical to that found in the pRAY plasmid^44^ was located 37 bp downstream of the fourth iteron and 11 bp upstream of *rep* start codon. The AT-rich element and the iterons presumably serve as the *oriV* site of the plasmid. Furthermore, ABLAC-p100050 and ABLAC_p100060 were found to encode homologues of RelE toxin of toxin/antitoxin system and Cro/C1 transcriptional regulator, respectively. However, no homologue of antitoxin partner gene was found on the plasmid. Many of predicted proteins of plasmid pABLAC1 loci (ABLAC_p100020 through ABLAC_p10002060) share identical amino acid sequences with those encoded by genes harbored by the plasmid (pD1279779) in *A. baumannii* strain D1279779^23^. On the other hand, plasmid pABLAC2 shares nearly 100% with a series of plasmids related to pRAY^44^,^45^, in particular pRAY*. Of particular note, two pABLAC2 loci (ABLAC_p200050 and ABLAC_p200060) encode homologues of MobA and MobC, respectively, which are known to be involved in plasmid mobilization; it is tempting to speculate that these mobilization genes may contribute to the transfer of the aminoglycoside resistance gene (ABLAC_p200010, *ant(2″)-Ia* or *aadB*) carried on this plasmid. Moreover, two copies of AT-rich sequence 5′-AAAAATAT-3′ were found within the coding region of a predicted ORF (ABLAC_p200080). However, no potential replication gene (*rep*) or iterons were found on pABLAC2. Similar to pRAY series of plasmids^44^,^45^, no plasmid partitioning or restriction/modification systems were identified in both plasmids of LAC-4.

### Comparative genomics of LAC-4 with 20 other completely sequenced A. baumannii genomes

**Figure 2:**
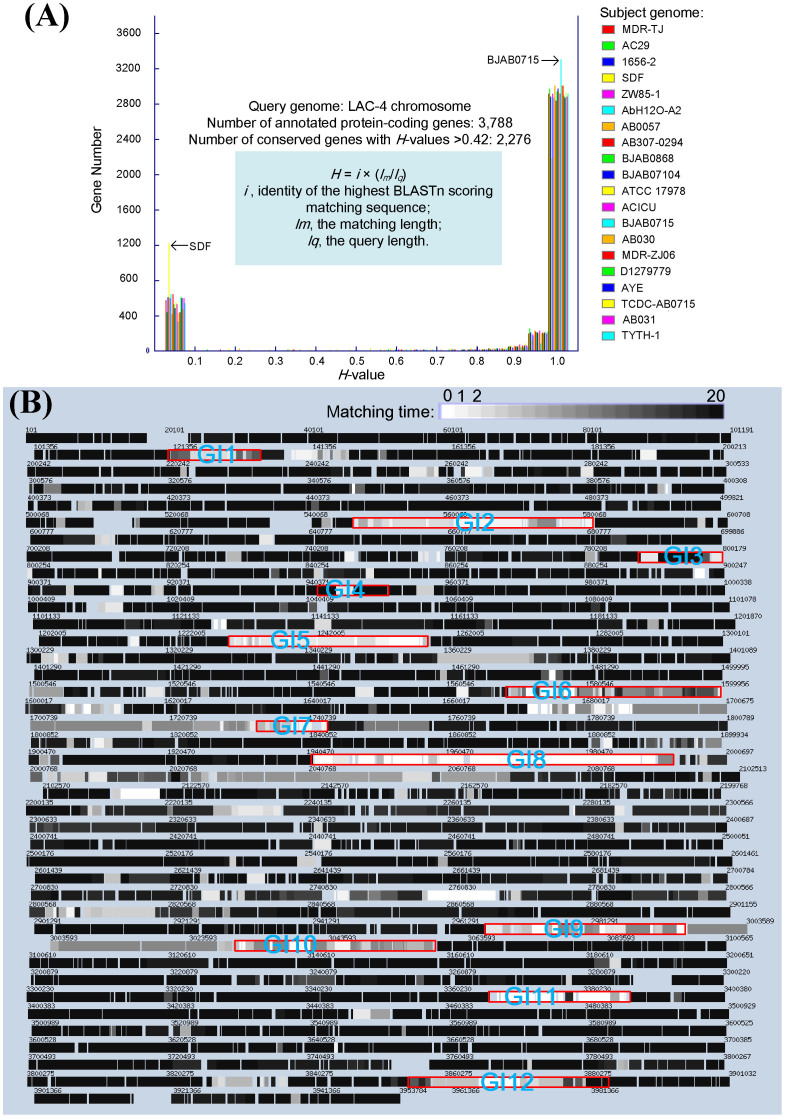
The mGenomeSubtractor-based *in silico* subtractive hybridization of the LAC-4 genome against genomes of twenty other completely sequenced *A. baumannii* isolates. The twenty subject *A. baumannii* chromosomes include: ATCC 17978 (NCBI accession no. NC_009085), SDF (NC_010400), AYE (NC_010410), ACICU (NC_010611), AB0057 (NC_011586), AB307-0294 (NC_011595), 1656-2 (NC_017162), MDR-ZJ06 (NC_017171), TCDC-AB0715 (NC_017387), MDR-TJ (NC_017847), TYTH-1 (NC_018706), D1279779 (NC_020547), BJAB07104 (NC_021726), BJAB0868 (NC_021729), BJAB0715 (NC_021733) and ZW85-1 (NC_023028) available at NCBI RefSeq project; and AB031 (GenBank accession no. CP009256), AC29 (CP007535), AB030 (CP009257) and AbH12O-A2 (CP009534) available at GenBank. (A) Histogram of BLASTn-based *H*-values for all 3,788 annotated protein-coding genes in the LAC-4 chromosome against all 20 subject chromosome sequences (color-coded). The *H*-value reflects the degree of similarity in terms of the length of match and the degree of identity at a nucleotide level between the matching gene in the subject genome and the query gene examined. The conserved genes were identified based on each of the obtained *H*-values great than 0.42. The genome of LAC-4 shows the most significant sequence identity to the BJAB0715 genome with 3,451 conserved genes (*H*-value > 0.42) among all the other 20 completely sequenced genomes; whereas, the lowest sequence identity to the SDF genome with 2,511 conserved genes (*H*-value > 0.42). (B) Chromosome map of LAC-4 with gene black/white-shade-coded based on the number of comparator *A. baumannii* genomes identified as harboring a nucleotide sequence-conserved homologue. Genes shown in absolute black (‘20’) are conserved across all 20 *A. baumannii* comparator genomes, with genes shown in decreasing shades of black being conserved in lower numbers of *A. baumannii* comparator genomes, while at the other extreme those shown in white (‘0’) are unique to LAC-4. Non-coding regions are shown as gaps. The genomic island-like hyper-variable regions (also see Table 3) are marked by red rectangles.

All protein-coding genes (CDSs) of LAC-4 were analyzed by mGenomeSubtractor-based *in silico* subtractive hybridization for presence of homologues against 20 other completely sequenced *A. baumannii* genomes. Our results indicate that the numbers of homologous CDSs (*H*-value > 0.42) among these genomes are 2,276 (Fig. 2A), representing 60% of the total CDSs in LAC-4. The LAC-4 chromosome is also presented in a continuous linear format showing the locations of all of its CDSs each with a degree of “blackness” reflecting relative conservation among the other 20 *A. baumannii* genomes (Fig. 2B). In particular, the LAC-4 genome exhibits the highest level of sequence identities to the BJAB0715 genome with 3,451 conserved genes (*H*-value > 0.42) (see Fig. 2A, 2B; Supplementary Fig. S1 online). When *Acinetobacter baumannii* genome groups are considered, LAC-4 was found to belong to Genome Group 3, represented by the genome of BJAB7015, as verified by a phylogenetic tree composed of 45 completely or partially sequenced *A. baumannii* genomes (see Supplementary Fig. S1 online).

**Figure 3:**
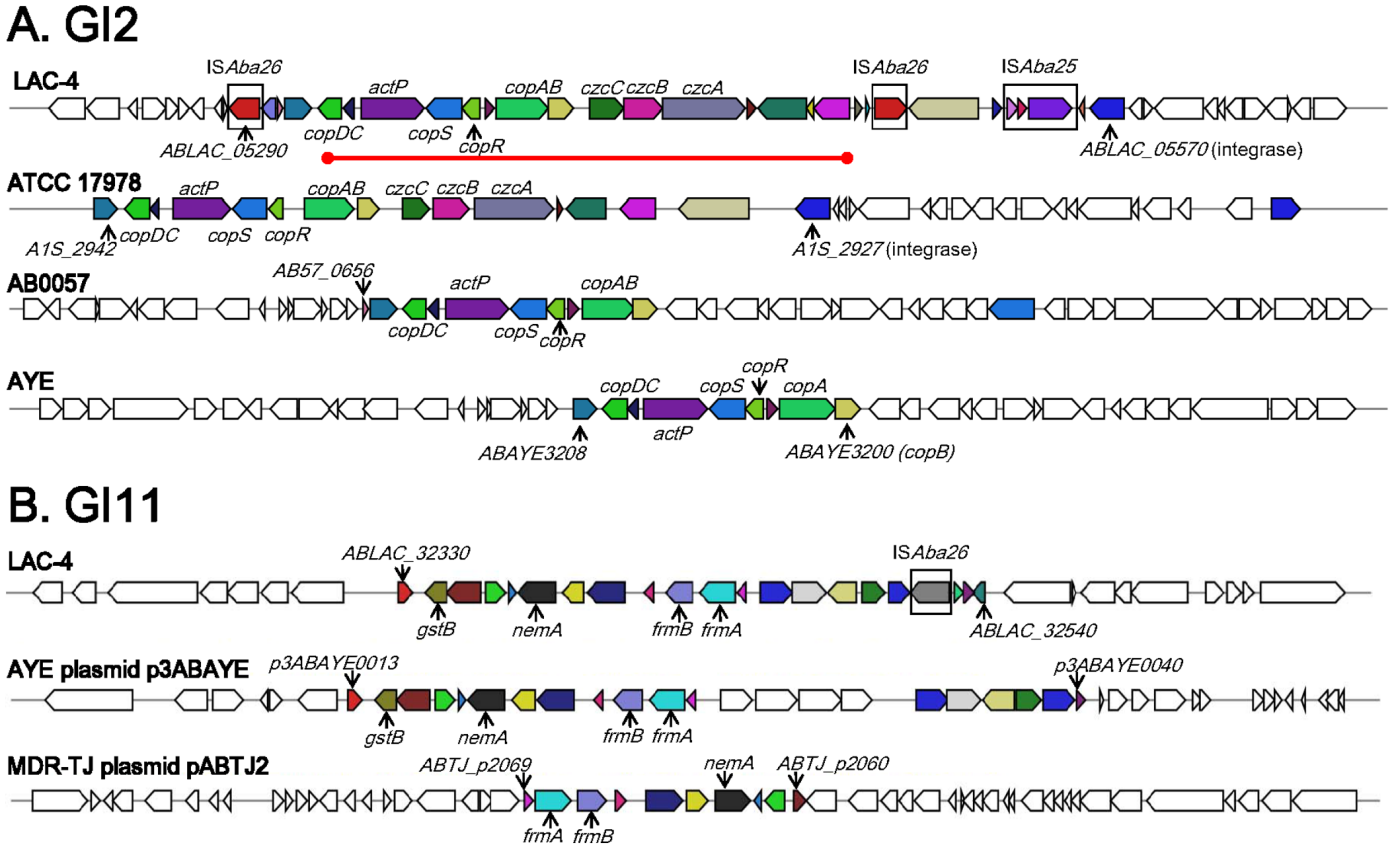
Alignment maps of the two representative *A. baumannii* LAC-4 genomic islands against related gene clusters among completely sequenced *A. baumannii* genomes. The BLASTp searches + hit collocation approach was used to generate the alignment, with matching genes shown as color-matched. (A) The 34-kb GI2 carries the copper resistance gene cluster (highlighted by a red line). (B) GI11 harbors genes predicted to encode enzymes for detoxification of xenobiotic compounds such as formaldehyde.

A total of 12 genomic islands were identified (Fig. 2B and Table 3). The GI1 (Table 3) consists of a novel transposon (Tn*6250*) composed of two IS*Aba1* elements at the ends, and an IS*1006* internally next to a partial IS*Vsa3* element (Fig. 1A). Additionally, this genomic island harbors resistance genes for streptomycin (*strA* and *strB*) and for sulphonamides (*sul2*), thus this GI is a resistance island (Table 3). The 34 kb GI2 contains two novel IS elements (IS*Aba26*) near the ends (Table 3 and Fig. 1B). Notably, these two IS elements sandwich a long 15-gene cluster (ABLAC_05330 to ABLAC_05470) coding for various proteins involved in copper resistance (Fig. 1B), representing the second resistance island found in LAC-4. Most of the genes of this gene cluster were also present in the genome of *A. baumannii* ATCC 17978, and to a lesser extent, in those of *A. baumannii* strains AB0057 and AYE (Fig. 3A). Outside one of the IS*Aba26* elements (ABLAC_05500), another novel IS element (IS*Aba25*) and a phage integrase gene (ABLAC_05570) are found (Table 3 and Fig. 3A), implicating the roles of mobile elements in the transfer of this GI. Furthermore, GI3 is bounded by two copies of a novel IS element (IS*Aba25*; transposase ORFs: ABLAC_07480-07500 and ABLAC_0758-07600) and it harbors a partial IS*Aba1* element and genes for the AdeIJK, which are components of an RND-type efflux pump system involved in resistance phenotypes of multiple classes of antibiotics (Fig. 1B).

**Figure 4:**
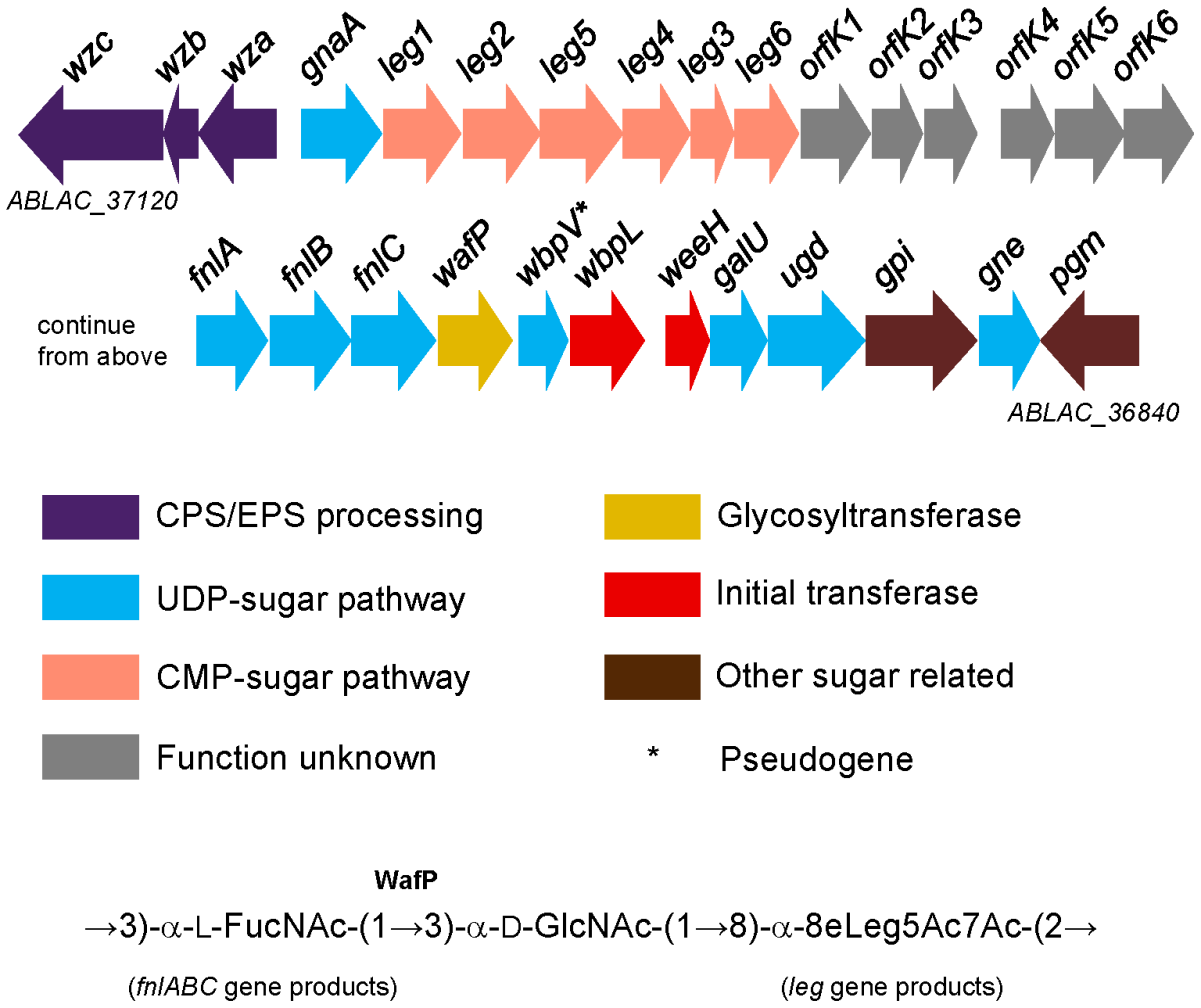
LAC-4 K locus gene organization and functional assignment. GI12 harbors genes of K locus in LAC-4. Color coding scheme follows that of Hu et al^63^. Two of the three-sugar repeating unit recently determined for LAC-4 surface polysaccharide^36^ can be produced via reactions catalyzed by gene products of the genes in the cluster (bottom). The product of *wafP* gene probably catalyzes the formation of the 1 → 3 glycosyl bond between α-L-FucNAC and α-D-GlcNAc. The *wbpV* gene (marked by *) is a pseudogene (with two separate ORFs) as a result of a point mutation.

Several GIs (GI5, GI6, GI7, GI8, GI9 and GI10) contain many genes encoding phage-derived proteins of unknown functions; the significance of these GIs remains to be elucidated (Table 3). Among these GIs, nearly all of the phage-derived genes of the GI8 have no homologues in any of the 20 other completely sequenced *A. baumannii* genomes (except several transposase genes and Zn-dependent protease gene) (data not shown). Moreover, GI11 contains a number of genes encoding putative proteins/enzymes with functions of detoxification of a variety of xenobiotic compounds (Fig. 3B); for example, glutathione S-transferase (ABLAC_32340, *gstB*), S-formylglutathione hydrolase (ABLAC_32430, *frmB*) and S-(hydroxymethyl)glutathione dehydrogenase (ABLAC_32440, *frmA*) are involved in resistance to and detoxification of formaldehyde and chlorine^46^. Interestingly, other than the *frmA* gene, most of the GI11 genes are unique to LAC-4 among the 21 completely sequenced *A. baumannii* genomes (data not shown). Finally, the GI12 contains the K (capsule) locus which encodes enzymes responsible for biosynthesis of the surface polysaccharide of LAC-4 (Fig. 4). Gene product homology analyses indicate that the K locus gene cluster harbors genes encoding two series of enzymes (FnlA, FnlB, FnlC and Leg1–6) which apparently catalyze biosynthesis of α-L-frucosamine and α-8-epi-legionaminic acid, respectively. These two sugars are the two precursor sugars (other than α-D-glucosamine, which is supplied by cellular metabolism) for the biosynthesis of three-sugar repeating unit of LAC-4 surface polysaccharide recently determined to consist of α-L-frucosamine, α-D-glucosamine and α-8-epi-legionaminic acid^36^. Interestingly, six ORFs (orfK1-6, ABLAC_36970–ABLAC_37020) in the middle of the gene cluster encode protein products (two of which are oxidoreductases) sharing no homology with any proteins in the 20 completely sequenced *A. baumannii* genomes (Fig. 4). Among the 20 *A. baumannii* strains whose genomes were completely sequenced, several have acquired resistance islands of various sizes^18^,^25^,^34^,^47^. For example, strain AYE harbors an 86-kb resistance island in which 45 resistance genes are located^34^. Similarly, MDR-TJ contains a 42-kb resistance island with four drug resistance genes^47^, while MDR-ZJ06 has a 38-kb resistance island (AbaR22) containing 40 genes, five of which are involved in antimicrobial resistance^25^.

### Antibiotic resistance of LAC-4 and resistance genetic determinants

The recent emergence and rapid dissemination of multidrug and pandrug resistant *A. baumannii* has caused significant burdens in clinical management of infections world wide. While we previously described antimicrobial susceptibility profile of LAC-4 towards 17 antibiotics, minimal inhibitory concentration (MIC) values were not presented^5^. Our MIC results of 23 antimicrobial drugs indicate that this strain is resistant to 13 of the 23 antibiotics (six of eight classes) tested, including ampicillin, carbenicillin, piperacillin, most of the cephalosporin analogs, three of four aminoglycosides, nalidixic acid and ciprofloxacin, trimethoprim and chloramphenicol (see Supplementary Table S2 online). Compared to the most highly resistant strains (AYE, ACICU, MDR-TJ and MDR-ZJ06) among the 20 *A. baumannii* strains whose genomes were completely sequenced, the MDR LAC-4's level of resistance is moderate (e.g., susceptible to imipenem and meropenem, intermediate to amikacin), which reflects the relatively early isolation of this strain (in 1997) from a hospital in Los Angeles. Nevertheless, the availability of complete genome sequences of *A. baumannii* strains with varied degrees of antimicrobial resistance (including the moderately resistant LAC-4) is highly useful for the scientific research community, especially from the standpoints of emergence and dissemination of antimicrobial resistance genes.

To better understand the repertoire of MDR genetic determinants and organization, in-depth analysis of the genome sequences of LAC-4 was performed. Consistent with the fact that LAC-4 was not among the most resistant strains analyzed in our 2008 report^5^, the LAC-4 genome harbors a moderate number of genetic determinants, some of which are linked to mobile genetic elements (IS or Tn), with a potential to encode resistance functions observed in this bacterial strain (Table 4). For example, genes potentially encoding for all four classes of β-lactamases (Classes A, B, C and D) have been found (Table 4). Two of such loci (*ampC* and *bla*_OXA-236_) are closely associated with IS*Aba1* elements (Fig. 1B and 1A, respectively) which may provide exogenous promoter functions, consistent with LAC-4 being resistant to piperacillin, older versions of the penicillins, and several cephalosporin antibiotics (see Supplementary Table S2 online). IS*Aba1* has also been found in the genomes of other *A. baumannii* strains associated with antibiotic resistance genes (including *ampC*), most likely driving robust expression of the resistance phenotypes^17^,^47^,^48^. While a *bla*_OXA-51-like_ gene (ABLAC_23600) is found in the LAC-4 genome (Table 4) as expected, the absence of *bla*_OXA-23-like_, *bla*_OXA-40-like_ and *bla*_OXA-58-like_ genes (data not shown) in the genome explains its susceptible phenotypes to imipenem and meropenem (see Supplementary Table S2 online). In contrast, in MDR strains AB0057, MDR-TJ, MDR-ZJ06, BJAB07104, BJAB0868 and BJAB0715, the presence of *bla*_OXA-23_ could account for their carbapenem resistance^17^,^18^,^25^,^47^. In addition, presence of aminoglycoside 6-phosphotransferase [*aph(6)-Id*, i.e., *strB*] and/or aminoglycoside 3″-phosphotransferase [*aph(3″)-Ib*, i.e., *strA*] were known to contribute resistance to streptomycin^49^. The existence of several aminoglycoside modification enzyme genes [ABLAC_01120 (*strA*), ABLAC_01130 (*strB*), and ABLAC_p200010 (*ant(2″)-Ia*)] in the LAC-4 genome (Table 4) and an association of an IS*Aba1* with two of these loci (*strA* and *strB*) in the context of a novel transposon Tn*6250* (Fig. 1A) correlate well with the strain's resistance to streptomycin, gentamicin, kanamycin and tobramycin (see Supplementary Table S2 onlinie). Finally, genes encoding a series of proteins for major facilitator superfamily (MFS), resistance-nodulation-division (RND) family, and multidrug and toxic compound extrusion (MATE) family of efflux pumps were also located in the LAC-4 genome (Table 4). Notably, there are at least five complete sets of RND type of efflux pump systems in LAC-4 (Table 4). Besides the AdeIJK genes linked with several IS elements described above in GI3 (Table 3 and Fig. 1B), four other sets of RND type efflux pump systems include AdeABC (encoded by ABLAC_26430–26450), AdeFGH (encoded by ABLAC_11920–11940), CzcABC (encoded by ABLAC_02860–02880) and homologues of CzcABC (Table 4). The last two sets have been shown to participate in transporting toxic metals such as cobalt, zinc and cadmium^50^,^51^. These efflux pump systems and other membrane-associated transporters (Table 4) confer resistance to diverse classes of antibiotics and other toxic compounds/metals in bacteria.

Many *A. baumannii* isolates have been reported to possess a series of efflux pump gene clusters in their genomes, which encode several types of chemical extrusion apparatuses rendering cells resistant to a large variety of antibiotics and toxic compounds^52^,^53^. For instance, there are three sets of RND efflux pump systems in *A. baumannii* strains (AdeABC, AdeIJK and AdeFGH)^54^,^55^,^56^. While LAC-4 genome harbors three gene clusters each of which separately encodes one of the above RND type efflux pumps, no apparent two-component system gene homologues *adeRS* was found upstream of *adeABC* gene. Such two-component systems (AdeRS) were found to be critical in regulating expression of AdeABC efflux pumps in several strains of *A. baumannii*^57^,^58^. Interestingly, Sun and colleagues observed that the insertion of an IS*aba1* into the *adeS* gene resulted in over-expression of *adeABC* operon hence the non-susceptibible phenotypes of clinical strains to tigecycline^59^. Additionally, AdeIJK appears to be regulated by AdeN, a TetR type of regulator in some strains^60^. On the contrary, the LAC-4's *adeIJK* genes exist in an unusual genomic context. These genes are not linked with any recognizable regulatory gene(s). Upstream of *adeIJK* genes, there exist a locus of unknown function (ABLAC_07540) and an apparent nonfunctional copy of IS*Aba1* element (Fig. 1B). All these genes are flanked by two copies of novel IS element IS*Aba25* (Fig. 1B). To our knowledge, this is the first report of a gene cluster encoding an RND efflux pump system being flanked by two IS*Aba25* elements, which may modify the expression of the efflux pump genes, thus conferring resistance to a number of antimicrobial agents.

### Potential virulence factors

Because our recent studies have shown that the LAC-4 strain is much more virulent than other *A. baumannii* strains in the mouse model of intranasal infection^33^, the LAC-4 genome was searched for genes coding for potential virulence factors against entries from VFDB. A total of 615 gene hits were generated which could facilitate the identification, evaluation and validation of virulence factors in this strain and the species (see Supplementary Table S3 online). Potential virulence factor loci that are unique for LAC-4 include ABLAC_03200 (which may encode a fimbrial protein, PilE), ABLAC_37000 and ABLAC_37010 [two genes of K locus gene cluster (Fig. 4)] (see Supplementary Table S3 online). In addition, several other potential virulence factor loci are found in only 1 to 4 genomes of the 20 completely sequenced *A. baumannii* genomes: ABLAC_05370 (encoding a transcriptional activator protein CusR/CopR), ABLAC_05450 (encoding an Fe^2+^ transport system protein FeoB) and K locus gene cluster genes ABLAC_36940–39960 [encoding UDP-N-acetylglucosamine 2-epimerase (FnlC), reductase (FnlB) and 4,6-dehydratase,3-and 5-epimerizase (FnlA)](see Supplementary Table S3 online and Fig. 4). Additional genes of the K locus gene cluster (ABLAC_36850, and ABLAC_36870–ABLAC_36910) were also identified as potential virulence factor genes (see Supplementary Table S3 online). Recently, Russo and colleagues identified, within the K locus gene cluster of *A. baumannii* strain AB307-0294, two loci *ptk* and *epsA* (encode a putative protein tyrosine kinase and a putative polysaccharide export outer membrane protein, respectively) that are required for capsule-positive phenotype, for survival in human serum and survival in a rat soft tissue infection model^61^; gene knock-out mutants of either gene were completely cleared from animals in the *in vivo* experiments. The encoded proteins of these two genes shared 90% and 95% identity over the entire protein lengths with the products of loci ABLAC_37120 and ABLAC_37100, respectively, suggesting importance of K locus gene cluster genes to the virulence of *A. baumannii*. More interestingly, as described above (Fig. 4), LAC-4's K locus gene cluster also contains a series of genes (*leg1*-*leg6*, ABLAC_37030–37080) apparently encoding enzymes necessary for biosynthesis of legionaminic acid, the precursor for an uncommon sugar (α-8-epi-legionaminic acid) found in the repeating unit of the surface polysaccharide of this strain^36^ and others^62^,^63^. It has been proposed that since legionaminic acid structurally resembles sialic acid on mammalian host cell's surface glycoproteins, bacterial pathogens may utilize this unusual sugar to mimic host cell surface, thus escaping from host immune surveillance to facilitate their colonization and invasion^64^,^65^,^66^. Since the legionaminic acid biosynthesis genes are not common among the 20 completely sequenced *A. baumannii* genomes (well conserved in only three of the 20 genomes), it is tempting to hypothesize that these genes may contribute to LAC-4's hypervirulence in mice. Furthermore, there are six ORFs (orfK1-6, ABLAC_36970–ABLAC_37020) in LAC-4's K locus that are absent in the other 20 completely sequenced genomes. It would be exciting to determine if any of these ORFs is involved in pathogenesis of this strain.

The ability of bacterial pathogens to obtain scarce iron from their mammalian hosts is critical for survival and infectivity^67^,^68^. Consequently, proteins that participate in uptake and utilization of iron or heme have been recognized as crucial virulence factors^69^,^70^,^71^,^72^. Not surprisingly, additional loci that were identified as putative virulence factor genes include genes from heme utilization cluster 1 (ABLAC_24390 and ABLAC_24450) and the *hemO* cluster (ABLAC_16780, ABLAC_16790 and ABLAC_16800) (see Supplementary Table S3 online). Also identified as potential virulence factor genes are many linked genes (ABLAC_10700, ABLAC_10710, ABLAC_10730, ABLAC_10750–ABLAC_10810, ABLAC_10840–ABLAC_10880) which are major constituents of a large 20-gene cluster (ABLAC_10690–ABLAC_10880) for the biosynthesis and transport of the acinetobactin siderophore in LAC-4 strain (see Supplementary Table S3 online); this gene cluster shares identical gene organization as described for the same gene clusters in several other *A. baumannii* genomes^73^. Similarly, a second siderophore biosynthesis and transport gene cluster (ABLAC_24780–ABLAC_24890) was identified from LAC-4 genome based on the presence of six of the loci within the gene cluster (ABLAC_24780–ABLAC_24820, ABLAC_24850 and ABLAC_24890) being on the list of potential virulence genes in LAC-4 (see Supplementary Table S3 online). The proteins encoded by the genes of this cluster and gene organization are identical to the cluster 5 described previously^73^ for a putative hydroxamate siderophore.

Our previous results^35^ and those of others^73^ suggested that a heme utilization gene cluster including a gene encoding for heme oxygenase (thus called *hemO* cluster) was present only in some *A. baumannii* strains and may account for their enhanced virulence. Bioinformatics analysis confirmed the presence of a *hemO* gene cluster in the LAC-4 genome (ABLAC_16780 to ABLAC_16850). Furthermore, comparative genomics analyses using BLASTp search + hit colocation analysis showed that 11 of the other 20 complete genomes of *A. baumannii* strains also contain the *hemO* cluster (see Supplementary Fig. S2 online). Another heme utilization gene cluster (without *hemO* gene; to be called heme utilization cluster 1) was reported to be present in all strains of *A. baumannii* tested^73^. As predicted, LAC-4 also harbors heme utilization cluster 1 (ABLAC_24350–ABLAC_24460), which is present in all the other 20 *A. baumannii* genomes (see Supplementary Fig. S3 online). The presence of *hemO* cluster in LAC-4 and 19 other outbreak isolates^5^ was further investigated by PCR. All 20 outbreak isolates (including LAC-4) were found to contain the *hemO* cluster (Table 1; see Supplementary Fig. S4 online), suggesting the relatively common presence of this gene cluster in this collection of clinical isolates. In this regard, our previous studies have shown that LAC-5, LAC-7, LAC-11 and LAC-16 are no more virulent than ATCC 17978, ATCC 17961 or clinical isolate AYE in the mouse model of intranasal infection^33^. Taken together, our data suggest that the presence of *hemO* cluster *per se* may not entirely account for the hypervirulence of LA-4. Indeed, the LAC-4 genome analysis identified four gene clusters for iron/heme utilization (the *hemO* cluster, the heme utilization cluster 1 and two gene clusters for siderophore biosynthesis and transport). It is highly likely that these gene clusters provide redundant function to ensure efficient acquisition of iron for cellular use. Further genome-wide molecular genetics studies (such as transposon mutagenesis) will be needed to decipher the relative contribution of genes or gene clusters of the iron/heme utilization and other processes in the virulence of this strain.

In summary, here we reported the complete genome sequence of a hypervirulent, multidrug resistant clinical outbreak isolate (LAC-4) of *A. baumannii* with an extremely rare MLST sequence type ST10 (MLST Pasteur Scheme). Among the 20 strains of *A. baumannii* whose complete genomes were available before this report, there are three strains of ST1, nine strains of ST2, two of ST79, one each for ST17, ST23, ST267, ST437, ST638 and ST639 (see Supplementary Table S4 online). Thus, the LAC-4 genomics study reported here represents the first complete genome sequence of an ST10 *A. baumannii* strain, making an important addition to the growing list of complete *A. baumannii* genomes for the scientific research community world-wide. Additionally, molecular tests and comparative genomics analyses offer insight in the mechanisms of resistance and virulence in this important bacterium.

## Methods

### Bacterial strains

The 20 outbreak strains (LAC-1 to LAC-20) were collected from four apparent clinical nosocomial outbreaks from 3 hospitals in Los Angeles County between 1996 and 2004 and obtained from Los Angeles County Public Health Laboratory^5^. The antimicrobial susceptibility and genetic profiles of these non-duplicate isolates were described in details previously^5^. Specifically, strains LAC-1 through LAC-5 were isolated from an outbreak in Hospital A that lasted for several years (1996–1999), while LAC-6 to LAC-10 were from the 2001 outbreak in the same hospital^5^. Separately, strains LAC-11 to LAC-15 were obtained from an outbreak in Hospital B during 2003–2004. Furthermore, there was an outbreak in Hospital C spanning 1997 and 1998 where LAC-16 to LAC-20 were archived^5^.

### Clonal relationships and sequence typing

Tri-locus multiplex PCR as described by Turton and colleagues^74^ was used to determine clonal relationships of these *A. baumannii* clinical isolates [Global Clones (GC) I, II or III, also known as International Clones I, II or III]. Multi-locus sequence typing (MLST) was performed based on methods of Diancourt et al^75^. PCR amplification was performed in separate reactions of a final volume of 50 μl. After amplification, aliquots of the PCR reactions were subject to agarose gel electrophoresis analysis. If successful, PCR products were purified using a QIAquick PCR purification kit (Qiagen, Valencia, CA) and then sequenced on an ABI 3730 automated fluorescent sequencer. Determination of the sequence type was carried out using details on the MLST scheme at www.pasteur.fr/mlst. To obtain MSLT ST assignments for *A. baumannii* strains whose genomes are complete, genome FASTA sequences were retrieved from NCBI and uploaded in the “batch sequence query” mode of MLST (Pasteur) database on the *Acinetobacter baumannii* MLST Databases website (http://pubmlst.org/abaumannii/), which will generate allelic profiles and STs.

### Genomic DNA sequencing, assembly and gap closing

The LAC-4 strain was grown overnight at 37°C to stationary phase in LB medium and total DNA was isolated from harvested cells. The genome sequence of LAC-4 was first determined using Roche 454 FLX+ and assembled with the GS *De Novo* Assembler, resulting in sequences with 43.5-fold coverage. Then 105 large contigs (>500 kb) were obtained by a combination of re-sequencing by Illumina Hiseq2000 (paired-end sequencing for 400-bp library, with 255.6-fold coverage) and Illumina Miseq (mate-pair sequencing for 3000-bp library, with 408-fold coverage). Finally, gaps between these contigs were closed by genomic PCR and sequencing of PCR products using conventional Sanger method (Applied Biosystems 3730 Genetic Analyzer). An integrity check that performs a BLASTp analysis on neighboring pairs of proteins identified 37 pairs of proteins, which may represent single genes that either have gained mutations or have split into two open reading frames (ORFs) due to sequencing errors. Additional PCR and DNA sequencing analysis have either corrected sequencing mistakes (likely as a result of 454 sequencing errors around homopolymer nucleotides) or combined gene pairs into single pseudogenes.

### Genome annotation and comparative genomics analysis

The replication origin (*oriC*) in LAC-4 genome was predicted by OriFinder^76^. The assembled genome sequences were annotated by using programs Glimmer 3.02 for identification of protein-coding sequences (CDSs)^77^ with manual validation of predicted CDSs on the basis of annotations of the BJAB7015 genome^17^ and the CBMAR database^78^, tRNAscan-SE for tRNA genes^79^, and RNAmmer for rRNA genes^80^. CDS functions were predicted using BLASTp searches^81^ of the NCBI non-redundant database followed by manual curation using the annotations of *A. baumannii* ATCC 17978 (NCBI accession no. NC_009083.1)^19^ and BJAB7015 (NC_021733.1)^17^ as references.

The virulence factor genes were predicted with the BLASTp searches against the virulence factor database (VFDB)^82^. The mobile genetic elements in the LAC-4 genome sequences were detected by the following online tools and/or open-access database and manual examinations: MobilomeFINDER for the tRNA/tmRNA gene-related genomic islands (GI)^83^, IslandViewer for the island-like regions^84^ and IS Finder for insertion sequence (IS) elements^85^. New IS names were provided by the curators of IS database^85^, while transposon (Tn) numbers were assigned by the Tn Number Registry^86^. The gene clusters carried by genomic islands (GIs) or involved in the heme utilization in the sequenced *A. baumannii* genomes were aligned by using the standalone package MultiGeneBlast^87^, an approach also known as “BLASTp searches + hit collocation”.

The genome sequence comparisons of LAC-4 with the other 20 completely sequenced *A. baumannii* strains were performed with the rapid multiple genome alignment tool mGenomeSubtractor^88^. All the annotated LAC-4 protein-coding genes (served as query) were examined by mGenomeSubtractor-facilitated BLASTn searches, using an *H*-value cut-off > 0.42 for conserved genes, against the other *A. baumannii* genomes (served as subject). The *H*-value (0 < = *H*-value < = 1.0) reflects the degree of similarity in terms of the length of match and the degree of identities at a nucleotide level between the matching gene in the subject genome and the query gene examined^88^.

### GenBank accession number

The genome sequences of the LAC-4 chromosome and two plasmids pABLAC1 and pABLAC2 have been submitted to the GenBank under accession numbers CP007712, CP007713 and CP007714, respectively.

### Antimicrobial susceptibility testing

Antimicrobial susceptibility of the clinical isolates or strains were determined using the broth microdilution protocols of Clinical Laboratory Standards Institute^89^ according to methods described previously^5^. *Escherichia coli* (ATCC #25922) and *Pseudomonas aeruginosa* (ATCC #27853) were used as quality control strains in the testing.

### Molecular detection of hemO cluster genes

PCR primer pairs specific for each gene of the eight-gene *hemO* cluster^35^ were designed based on alignment of gene cluster sequences of six *A. baumannii* strains [strains ACICU^21^, SDF^22^, MDR-ZJ06^25^, TYTH-1^27^, TCDC-AB07115^26^, and AB0057^18^], available as of May, 2013. The sequence alignment was performed by using Lasergene MegAlign program from DNASTAR, Inc. (Madison, WI). Primers were chosen from regions of highly conserved sequences and are shown in Supplementary Table S5 online. The PCR amplification of the *hemO* gene cluster was performed for the clinical isolates using genomic DNA as a template. Individual PCR reactions involved a 50 μl reaction mixture containing 1 × 5 PRIME MasterMix, 200 nM of forward and reverse primers, and 1 μl of genomic DNA template. Amplification was carried out with 5 min at 95°C; 36 cycles of 30 s at 95°C, 30 s at 55°C, and 1 min at 72°C; and 10 min at 72°C. The resulting PCR products were visualized and imaged through agarose gel electrophoresis analysis.

## Author Contributions

H.H.X., X.H., H.Y.O. and W.C. conceived the study. X.H., S.N.K., B.M.M. and P.J.E. performed experiments. H.Y.O. and H.H.X. performed genomics and bioinformatics analyses. H.H.X., H.Y.O., W.C., X.H., Z.D. and M.O. wrote the paper. All authors read and approved the final manuscript.

## Supplementary Material

Supplementary Information Supplementary Information

## Figures and Tables

**Table 1:** Molecular tests for 20 clinical isolates of *A. baumannii* obtained from Los Angeles County, CA

		MLST allelic profile								
Strain code	TLM-GC	*cpn60*	*fusA*	*gltA*	*pyrG*	*recA*	*rplB*	*rpoB*	MLST-ST	*hemO* cluster
LAC-1	NT	1	3	2	1	4	4	4	10	+
LAC-2	NT	1	3	2	1	4	4	4	10	+
LAC-3	NT	1	3	2	1	4	4	4	10	+
LAC-4	NT	1	3	2	1	4	4	4	10	+
LAC-5	NT	40	3	15	2	40	4	4	241	+
LAC-6	NT	1	2	2	2	11	1	5	417	+
LAC-7	NT	1	2	2	2	11	1	5	417	+
LAC-8	NT	40	3	15	2	40	4	4	241	+
LAC-9	NT	1	2	2	2	11	1	5	417	+
LAC-10	NT	1	2	2	2	11	1	5	417	+
LAC-11	II	2	2	2	2	2	2	2	2	+
LAC-12	II	2	2	2	2	2	2	2	2	+
LAC-13	II	2	2	2	2	2	2	2	2	+
LAC-14	II	2	2	2	2	2	2	2	2	+
LAC-15	NT	1	2	2	2	11	1	5	417	+
LAC-16	NT	1	3	2	1	4	4	4	10	+
LAC-17	NT	1	3	2	1	4	4	4	10	+
LAC-18	NT	1	3	2	1	4	4	4	10	+
LAC-19	NT	1	3	2	1	4	4	4	10	+
LAC-20	NT	1	3	2	1	4	4	4	10	+

TLM-GC, Trilocus multiplex assay Global Clone designation.

MLST-ST, Multi-locus sequence type.

NT, not typable.

**Table 2:** General features of the *A. baumannii* LAC-4 genome

Feature	Chromosome	pABLAC1	pABLAC2
Total number of base pairs	3,954,354	8,006	6,076
G + C content (%)	39	32	39
Number of protein-coding genes^a^	3,788	9	8
Number of rRNA operons	6	0	0
Number of tRNA/tmRNA genes	73	0	0
Number of genomic island-like regions	12	N/A^b^	N/A
	19 (IS*Aba1*)		
	14 (IS*Aba125*)		
Number of Insertion Sequences (ISs)	22 (IS*Aba13*)	0	0
	12 (IS*Aba25*)		
	14 (IS*Aba26*)		

^a^These features were obtained based on annotations using the Glimmer 3.02 program.

^b^NA, not applicable.

**Table 3:** Genomic island (GI)-like regions identified in the LAC-4 chromosome

Region	Coordinates [CDS]	Length (kb)	G + C%^a^	Features
GI1	120,830–133,655 [ABLAC_01100–01230]	12.8	42	Novel composite transposon Tn*6250* (formed by IS*Aba1* elements) encompassing entire island containing IS*1006*, partial IS*Vsa3*, streptomycin and sulphonamide resistance genes, conjugal transfer functions TraA and TraD
GI2	547,857–581,621 [ABLAC_05290–05570]	33.7	38	Novel IS elements (IS*Aba26*) flanking a 15-gene copper resistance cluster; another novel IS element (IS*Aba25*); integrase
GI3	788,377–800,975 [ABLAC_07480–07600]	12.6	43	Novel IS elements (IS*Aba25*); RND-type multidrug efflux pump proteins AdeI, AdeJ and AdeK and a partial IS*Aba1*.
GI4	940,403–951,920 [ABLAC_08880–08980]	11.5	40	IS*Aba25*; alpha/beta hydrolase superfamily; a gene encoding for ComEC-like protein; enzymes for biosynthesis of lipoproteins; purine biosynthesis enzymes
GI5	1,229,550–1,257,942 [ABLAC_11500–11860]	28.4	40	Insertion site: Arg tRNA gene (ABLAC_t00580); phage proteins
GI6	1,571,326–1,611,225 [ABLAC_14900–15460]	39.9	39	Two IS*Aba13* elements; mostly phage proteins
GI7	1,730,338–1,741,047 [ABLAC_16600–16720]	10.7	35	Integrase; phage proteins
GI8	1,941,480–1,993,076 [ABLAC_18640–19030]	51.6	39	Insertion site: Ser tRNA gene (ABLAC_t00260); IS*Aba13* and IS*Aba25*; phage proteins
GI9	2,963,891–2,993,411 [ABLAC_28350–28700]	29.5	36	Mostly phage protein; IS*Aba13*, IS*Aba125*, IS*Aba25*, IS*Aba26*
GI10	3,028,724–3,058,412 [ABLAC_29030–29480]	29.7	35	Insertion site: tmRNA gene (ABLAC_t00730); phage proteins; IS*Aba13*
GI11	3,367,720–3,386,189 [ABLAC_32320–32540]	18.5	40	IS*Aba26*; *gstB*, *frmA* and *frmB* genes; Genes similar to the plasmid p3ABAYE and pABTJ2 are found
GI12	3,852,499–3,882,676 [ABLAC_36850–37160]	33.4	35	The K locus, which consists of genes encoding enzymes involved in biosynthesis of capsular polysaccharides, including two series of enzymes responsible for biosynthesis of α-L-frucosamine and α-8-epi-legionaminic acid, two sugars of the surface polysaccharide repeating units; six ORFs of unknown function which are unique for LAC-4 among completely sequenced *A. baumannii* genomes.

^a^The G + C content of the LAC-4 chromosome is 39%.

**Table 4:** LAC-4 genes associated with antimicrobial resistance

Antimicrobial agent class	Gene products/function	Gene name (if available)	LAC-4 protein locus tag
β-lactams and cephalosporins	Class A β-lactamase		ABLAC_20980
	Class B β-lactamase (metallo-β-lactamase superfamily)		ABLAC_06190, ABLAC_08350, ABLAC_25540, ABLAC_27020, ABLAC_27490, ABLAC_34080
	Class C β-lactamase	*ampC*	ABLAC_10940,
			ABLAC_14670, ABLAC_34320
	Class D β-lactamase	*bla*_OXA-236_	ABLAC_13990
		*bla*_OXA-68_ (*bla*_OXA-51-like_)	ABLAC_23600
Aminoglycosides	Aminoglycoside 6-phosphotransferase	*strB* [*aph(6)-Id*]	ABLAC_01130
	Aminoglycoside 3″-phosphotransferase	*strA* [*aph(3″)-Ib*]	ABLAC_01120
	Aminoglycoside 2″-nucleotidyltransferase	*ant(2″)-Ia*	ABLAC_p200010
Phenicols	Chloramphenicol acetyltransferase		ABLAC_08080
Fluoroquinolones	Mutated GyrA (Ser-83-Leu),Mutated ParC (Glu-84-Lys)	mutated *gyrA, parC*	ABLAC_08740, ABLAC_35720
Folate pathway inhibitors	Dihydropteroate synthase		ABLAC_08190
	Dihydropteroate synthase type 2	*sul2*	ABLAC_01210
Heavy metals	Copper resistance protein CopD,Copper resistance protein CopC,Copper-exporting ATPase,Heavy metal sensor kinase CopS,Transcriptional activator protein CopR,Copper-resistance protein CopA,Copper resistance protein CopB,Heavy metal RND efflux outer membrane protein CzcC-like,Copper binding periplasmic protein CzcB-like,Cation efflux system protein CzcA-like	*copD, copC, actP, copS, copR, copA, copB, czcC-like, czcB-like, czcA-like*	ABLAC_05320–ABLAC_05470
	Chromate transport protein,Chromate transporter	*chrA*	ABLAC_22900ABLAC_22910
Multiple agents			
	MFS (major facilitator superfamily) efflux pump		
			ABLAC_00380
			ABLAC_19630
			ABLAC_13820
			ABLAC_06230
	Bcr/CflA subfamily	*bcr/cflA*	ABLAC_06830
	MFS permease		ABLAC_31900
	RND (resistance-nodulation-division) family efflux pump		
	Membrane fusion protein	*acrA-like*	ABLAC_00310
	RND pump protein	*acrB-like*	ABLAC_00320, ABLAC_06650, ABLAC_11930
	Membrane fusion protein	*adeA*	ABLAC_26450 (partial gene)
	RND pump protein	*adeB*	ABLAC_26440
	Outer membrane protein	*adeC*	ABLAC_26430
	Membrane fusion protein	*adeI*	ABLAC_07530
	RND pump protein	*adeJ*	ABLAC_07520
	Outer membrane protein	*adeK*	ABLAC_07510
	Membrane fusion protein	*adeF*	ABLAC_11940
	RND pump protein	*adeG*	ABLAC_11930
	Outer membrane protein	*adeH*	ABLAC_11920
	RNA pump protein	*adeB-like*	ABLAC_08370
	Co/Zn/Cd efflux system RND type	*czcA*	ABLAC_02870
		*czcB*	ABLAC_02860
		*czcC*	ABLAC_02860
	Copper efflux system RND type	*czcA-like*	ABLAC_05430
		*czcB-like*	ABLAC_05420
		*czcC-like*	ABLAC_05410
	MATE (multidrug and toxic compound extrusion) family efflux pump		
		*norM*	ABLAC_01030
		*matE*	ABLAC_00610
	SMR (small multidrug resistance) family protein	*qacEΔ1-like*	ABLAC_06380
	ABC transporter		ABLAC_14500
	Cadmium, cobalt and zinc/H(+)-K(+) antiporter	*czcD*	ABLAC_02880
