# Supplementary material for: Complete genome sequence of hypervirulent and outbreak-associated Acinetobacter baumannii strain LAC-4: epidemiology, resistance genetic determinants and potential virulence factors
Source: Sci Rep. 2015 Mar 2;5:8643. doi: 10.1038/srep08643 (PMC4345345; doi:10.1038/srep08643)
Supplement: Supplementary Information [file srep08643-s1.pdf]

SUPPLEMENTARY INFORMATION

Complete genome sequence of hypervirulent and outbreak-associated *Acinetobacter baumannii* strain LAC-4: epidemiology, resistance genetic determinants and potential virulence factors

Hong-Yu Ou[1], Shan N. Kuang[2], Xinyi He[1], Brenda M. Molgora[2], Peter J. Ewing[2], Zixin Deng[1], Melanie Osby[3]†, Wangxue Chen[4] and H. Howard Xu[2]*

[1]State Key Laboratory of Microbial Metabolism and School of Life Sciences & Biotechnology, Shanghai Jiaotong University, Shanghai, China; [2]Department of Biological Sciences, California State University Los Angeles, Los Angeles, California, USA; [3]Department of Pathology, LAC+USC Medical Center, Los Angeles, California, USA; [4]Human Health Therapeutics, National Research Council Canada, 100 Sussex Drive, Ottawa, Ontario K1A 0R6, Canada

†Current address: Martin Luther King, Jr. Outpatient Center, Los Angeles, California, USA.

*Correspondence and requests for materials should be addressed to H.H.X (hxu3@calstatela.edu)

Running title: Complete genome sequences of *A. baumannii* LAC-4

Key words: *Acinetobacter baumannii*, LAC-4, bacterial genome, antibiotic resistance, epidemiology, genomic island

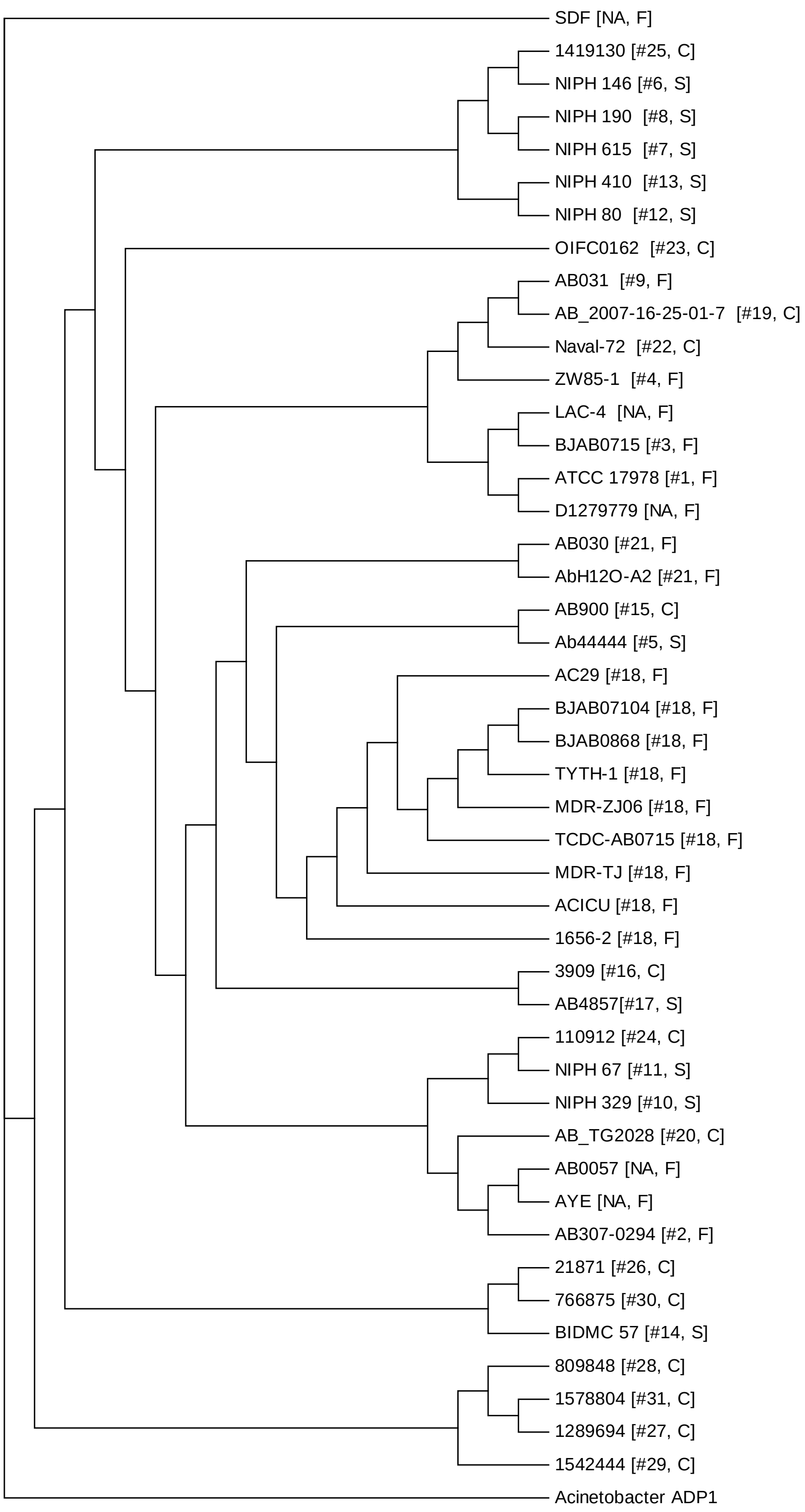

Supplementary Figure S1. Inferred phylogenetic relationships of the 45 sequenced *Acinetobacter baumannii* genomes, with the genome of *Acinetobacter* sp. ADP1 used as the out-group to root the tree. The neighbor-joining tree was generated with CVTree [1] by comparing their proteomes using a composition vector approach (K = 6). *A. baumannii* isolate names are followed by square brackets to indicate the genome group number given by NCBI (NA, denoting Not Available) and the genome assembly status (F, complete genome finished; S, scaffolds; C, contigs).

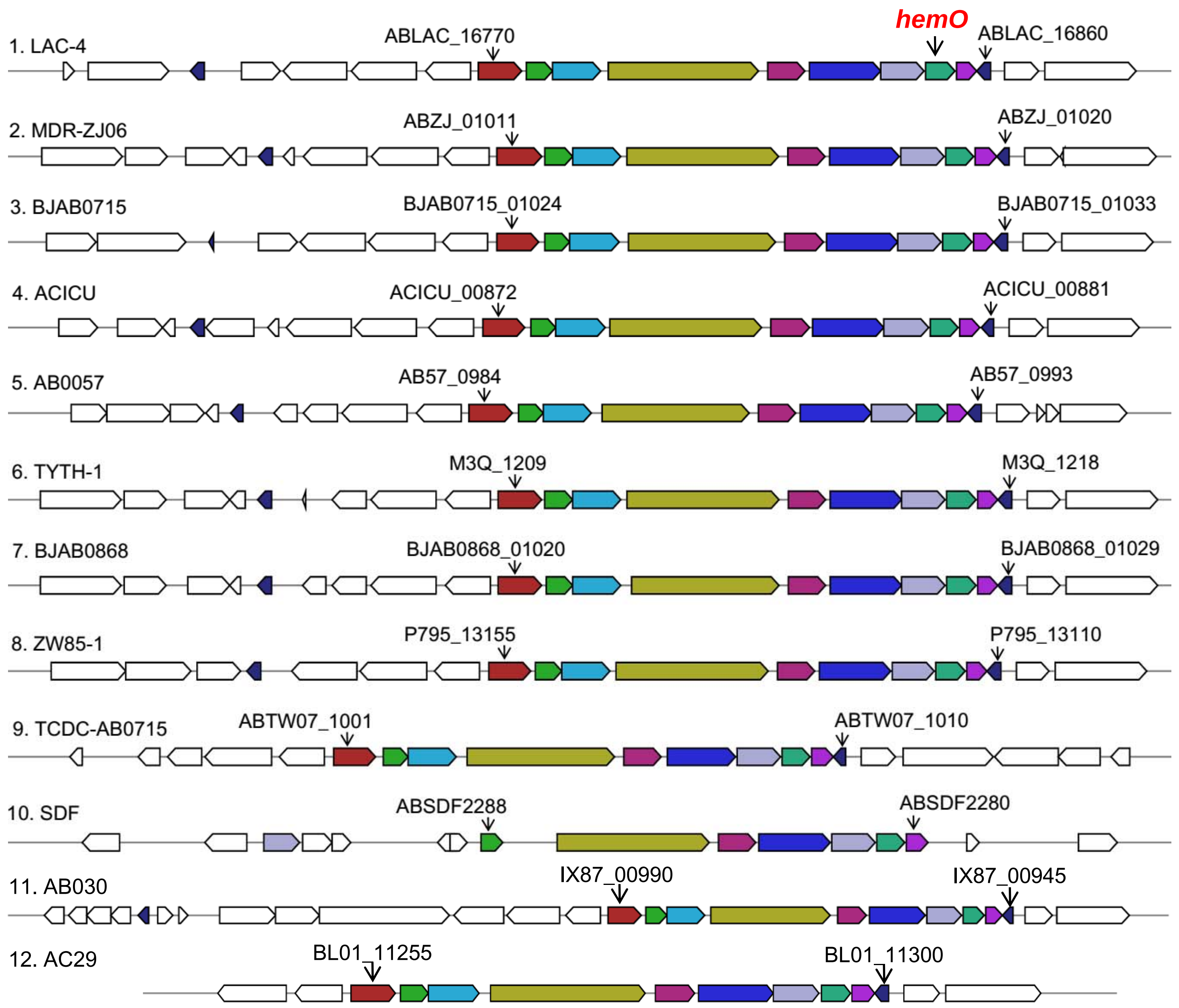

Supplementary Figure S2. Alignment of *hemO* clusters from 12 completely sequenced *A. baumannii* genomes. The BLASTp searches + hit colocation approach was used to generate the alignment, with matching genes shown as color-matched. Protein FASTA sequences of LAC-4 *hemO* cluster genes plus one additional locus on either direction (ABLAC_16770 - ABLAC_16860) were used as input. The *hemO* gene is marked by an arrow.

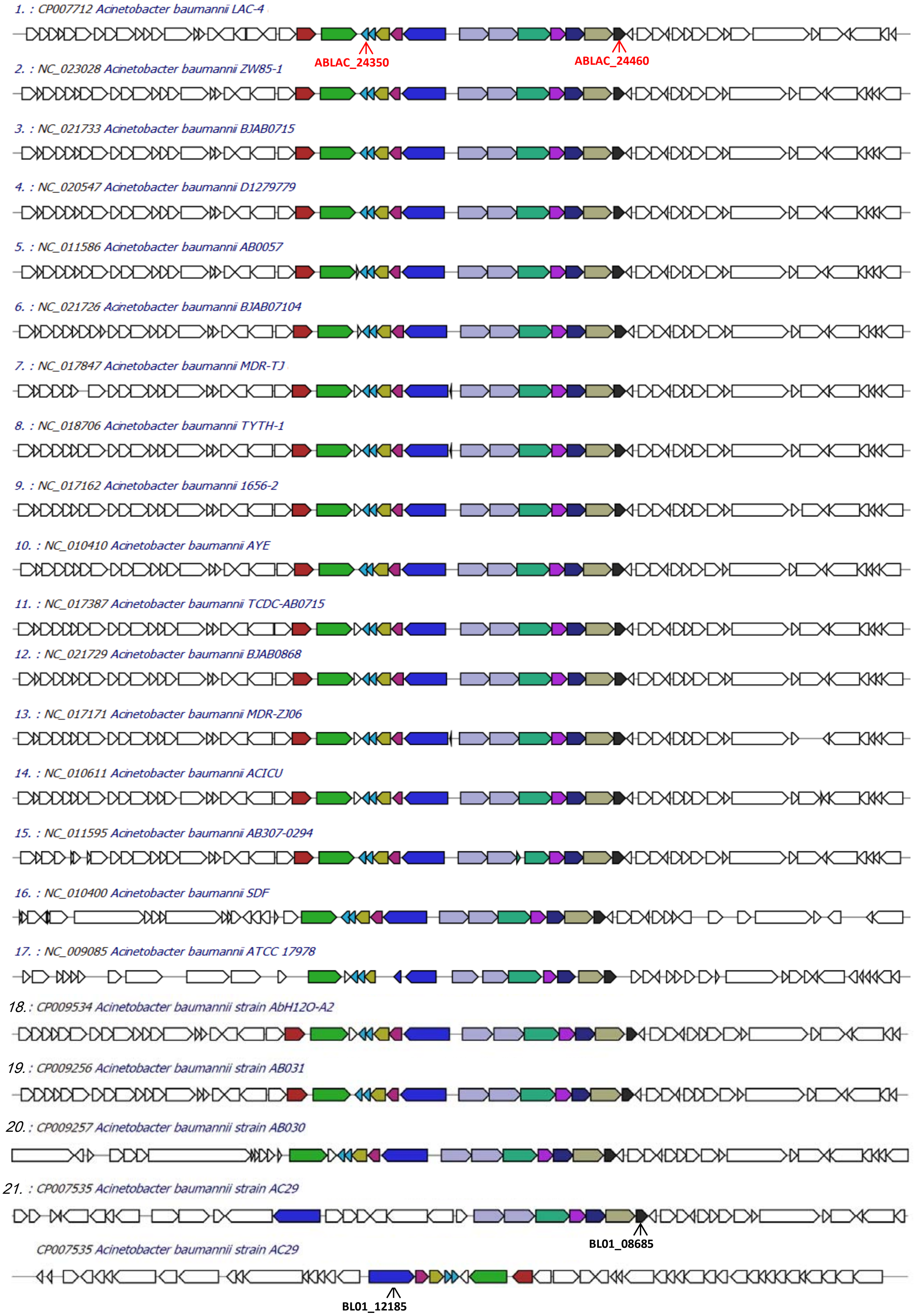

Supplementary Figure S3. Allignment of heme utilization cluster 1 sequences from 21 completely sequenced *A. baumannii* genomes. The BLASTp searches + hit colocation approach was used to generate the alignment, with matching genes shown as color-matched.

A

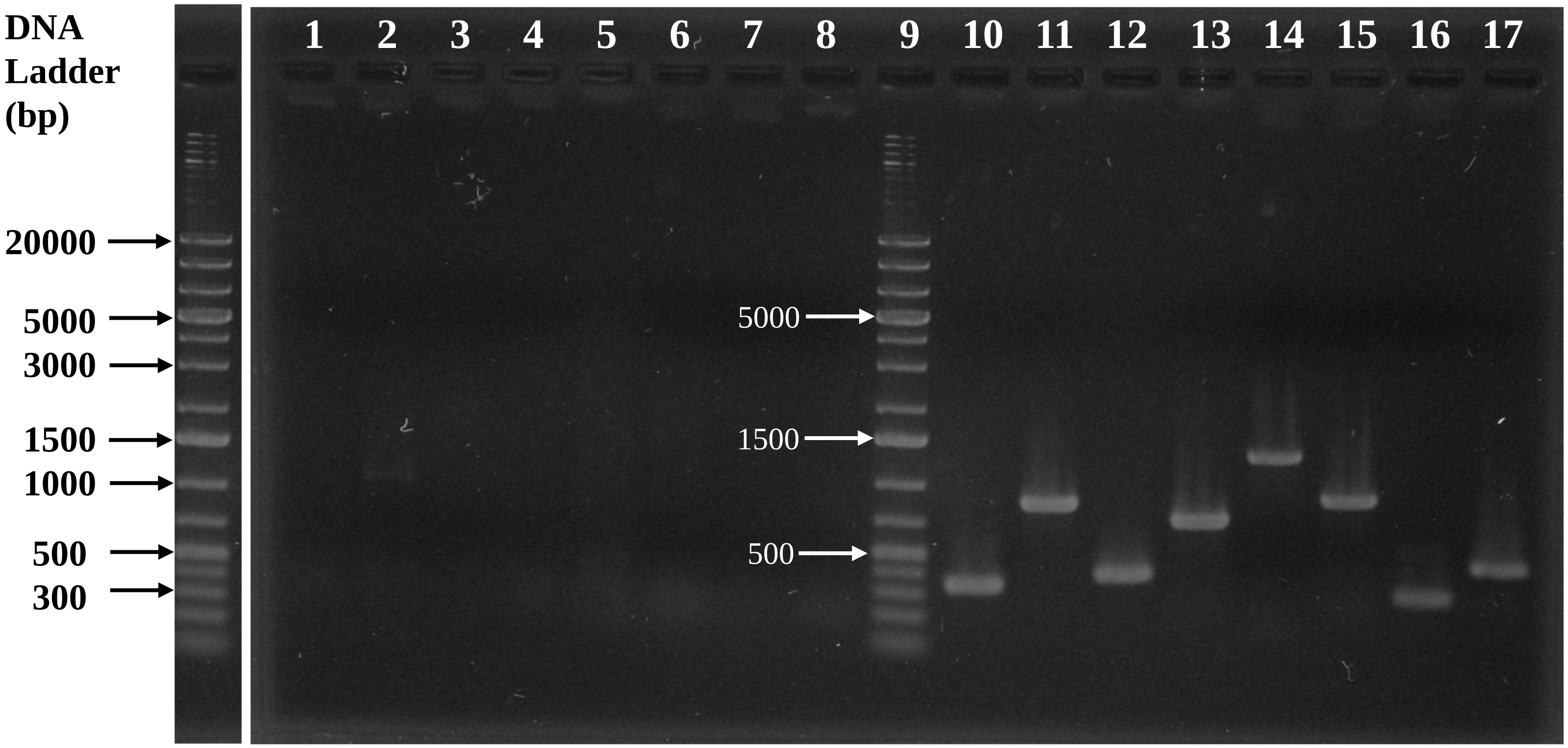

Supplementary Figure S4. Molecular detection of *hemO* cluster in *A. baumannii* isolates. PCR products were separated on agarose gels, with DNA ladder (1 kb plus from Fermentas) in lane 9. Lanes 1-8 contain amplicons using primer pairs for *hemO* cluster gene IDs (based on the ACICU genome [2]) 873, 874, 875, 876, 877, 878, 879, and 889 (using only the last three digit of the locus ID; e.g., ACICU_00873 is abbreviated as 873); similarly, lanes 10-17 contain amplicons using primer pairs for *hemO* cluster gene IDs (based on the ACICU genome [2]) 873, 874, 875, 876, 877, 878, 879, and 889; **A**. agarose gel electrophoresis results for negative control strain ATCC 17978 (lanes 1-8) and LAC-4 (lanes 10-17); **B**. agarose gel electrophoresis results for LAC-1 (lanes 1-8) and LAC-2 (lanes 10-17).

B

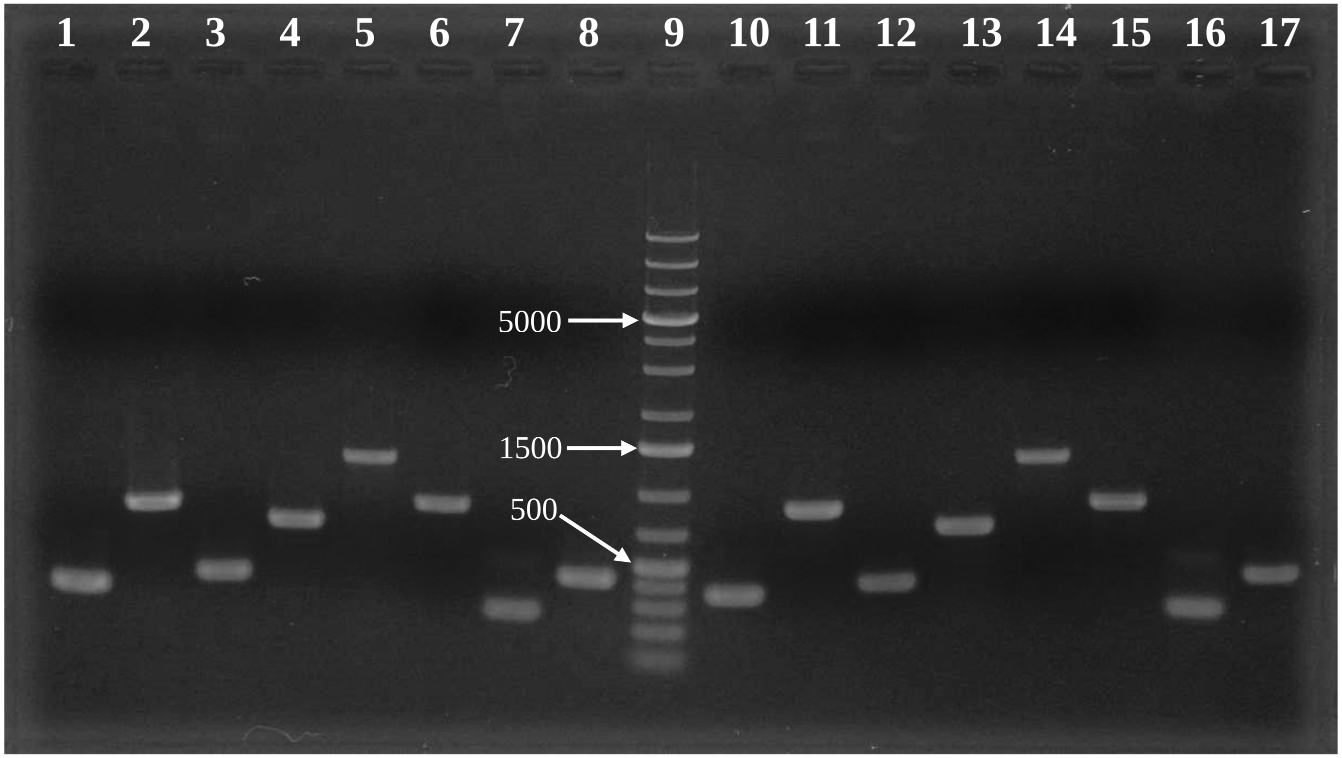

Supplementary Figure S4. Molecular detection of *hemO* cluster in *A. baumannii* isolates. PCR products were separated on agarose gels, with DNA ladder (1 kb plus from Fermentas) in lane 9. Lanes 1-8 contain amplicons using primer pairs for *hemO* cluster gene IDs (based on the ACICU genome [2]) 873, 874, 875, 876, 877, 878, 879, and 889 (using only the last three digit of the locus ID; e.g., ACICU_00873 is abbreviated as 873); similarly, lanes 10-17 contain amplicons using primer pairs for *hemO* cluster gene IDs (based on the ACICU genome [2]) 873, 874, 875, 876, 877, 878, 879, and 889; **A**. agarose gel electrophoresis results for negative control strain ATCC 17978 (lanes 1-8) and LAC-4 (lanes 10-17); **B**. agarose gel electrophoresis results for LAC-1 (lanes 1-8) and LAC-2 (lanes 10-17).

Supplementary Table S1. Insertion sequence types, genome coordinates and sequences of target site duplication if applicable

IS type	Genome coordinates		Orientation	Locus tag of tranposase gene(s)	Sequence of Target Site Duplication		Notes
					5' end	3' end	
ISAba1	120757	121938	-	ABLAC_01100, 01110	AAAAAATTG	not applicable	these two IS elements constitute a transposon
	132494	133673	+	ABLAC_01220, 01230	not applicable	AAAAAATTG	
	441008	442187	+	ABLAC_04150, 04160	ATATAATTA	ATATTAATTA	
	797218	798258	+	ABLAC_07560, 07570	ATATTTTTT	ATATTTTTT	Deletion within ORF
	835289	836468	-	ABLAC_07940, 07950	ATCTCTAAT	ATCTCTAAT	
	1036519	1037698	+	ABLAC_09850, 09860	AACTTACGA	not applicable	these two IS elements constitute a transposon
	1040661	1041840	+	ABLAC_09910, 09920	not applicable	AACTTACGA	
	1165840	1167019	+	ABLAC_10950, 10960	GATTAGCTC	GATTAGCTC	
	1442290	1443469	-	ABLAC_13690, 13700	ATGAAATAT	ATGAAATAT	
	1474005	1475184	-	ABLAC_13970, 13980	ATTTATTTA	not applicable	these two IS elements constitute a transposon
	1476092	1477271	+	ABLAC_14000, 14010	not applicable	ATTTATTTA	
	1649265	1650444	-	ABLAC_15840, 15850	AGGGATCTT	AGGGATCTT	
	1881264	1882443	+	ABLAC_18020, 18030	not detected	not detected	
	1889159	1890338	-	ABLAC_18130, 18140	CTAGATTTT	CTAGATTTT	
	2058343	2059522	-	ABLAC_19610, 19620	ATGTTTATA	ATGTTTATA	
	2388493	2389672	+	ABLAC_22790, 22800	not detected	not detected	
	2469997	2471036	+	ABLAC_23630, 23640	AATCTATTA	AATCTATTA	Deletion; IRL sequence changed
	3256026	3257205	+	ABLAC_31280, 31290	AAAAATGAC	AAAAATGAC	
	3393015	3394194	+	ABLAC_32600, 32610	AAAGTTTAT	AAAGTTTAT	
ISAba13	222235	223273	+	ABLAC_02030	AATAGAATG	AATAGAATG	
	257685	258723	-	ABLAC_02390	ATGTAAAGC	ATGTAAAGC	
	390705	391743	+	ABLAC_03660	TCTTTTATC	TCTTTTATC	
	407537	408575	+	ABLAC_03820	not detected	not detected	
	1459463	1460501	-	ABLAC_13800	TAATACAAT	TAATACAAT	
	1525267	1526305	-	ABLAC_14490	not detected	not detected	
	1569653	1570691	-	ABLAC_14880	CTTGTAGAC	CTTGTAGAC	
	1576364	1577402	+	ABLAC_14960	ATACAGTTT	ATACAGTTT	
	1607024	1608062	+	ABLAC_15430	CCTTCCAAT	CCTTCCAAT	
	1683240	1684278	+	ABLAC_16180	TATTTCATA	TATTTCATA	
	1805892	1806930	+	ABLAC_17320	TACTAAATT	TACTAAATT	
	1950373	1951411	-	ABLAC_18730	not detected	not detected	
	2025230	2026268	-	ABLAC_19320	TTTTAAAAG	TTTTAAAAG	
	2163300	2164338	+	ABLAC_20620	TCCACAAT	TCCACAAT	8-bp TSD
	2625079	2626117	-	ABLAC_25160	CCTTATAAT	CCTTATAAT	
	2802850	2803889	+	ABLAC_26860	not detected	not detected	
	2804794	2805832	-	ABLAC_26890	not detected	not detected	
	2968534	2969570	+	ABLAC_28430	not detected	not detected	
	2974685	2975723	-	ABLAC_28500	ATCTAATAT	ATCTAATAT	
	3028670	3029708	+	ABLAC_29030	TCCGAAATC	TCCGAAATC	
	3785007	3786045	-	ABLAC_36240	ATTTCATTA	ATTTCATTA	
	3831528	3832566	+	ABLAC_36690	not detected	not detected	

IS*Aba25*	577154	579644	+	ABLAC_05530, 05540, 05550	GGTTAATC	GGTTAATC	
	788291	790781	+	ABLAC_07480, 07490, 07500	TATCAATC	TATCAATC	
	798565	800961	-	ABLAC_07580, 07590, 07600	CACTTTTT	CACTTTTT	
	944564	947054	-	ABLAC_08910, 08920, 08930	ATTGCATC	ATTGCATC	
	1517251	1519741	+	ABLAC_14400, 14410, 14420	ATAGTAAA	ATAGTAAA	
	1944078	1946568	-	ABLAC_18670, 18680, 18690	AAAACAGC	AAAACAGC	
	2009185	2011677	+	ABLAC_19170, 19180, 19190	GAATAAAG	GAATAAAG	mutation causing trancated ORF
	2441386	2443876	+	ABLAC_23290, 23300, 23310	GGTGGTAA	GGTGGTAA	
	2631817	2634305	+	ABLAC_25240, 25250, 25260	CTCACAAA	CTCACAAA	
	2725437	2727927	-	ABLAC_26170, 26180, 26190	GTTCCGAT	GTTCCGAT	
	2966137	2968533	-	ABLAC_28400, 28410, 28420	not detected	not detected	trancated ORF
	3447038	3449528	-	ABLAC_33090, 33100, 33110	GGTTATAG	GGTTATAG	
IS*Aba26*	504386	505703	-	ABLAC_04900	AACAAATG	AACAAATG	
	547823	549140	-	ABLAC_05290	not detected	not detected	
	572154	573471	+	ABLAC_05500	TACCAGCT	TACCAGCT	
	780451	781768	-	ABLAC_07400	TGAATGAA	TGAATGAA	
	1902009	1903326	-	ABLAC_18290	not detected	not detected	
	2097204	2098521	+	ABLAC_19990	GATCCTAA	GATCCTAA	
	2209568	2210885	-	ABLAC_21090	TTTCCCCT	TTTCCCCT	
	2559209	2560526	+	ABLAC_24560	GACAAATA	GACAAATA	
	2697056	2698375	+	ABLAC_25880	TTGATAAT	TTGATAAT	
	2978037	2979354	-	ABLAC_28540	AAAAGCCC	AAAAGCCC	
	3003559	3004876	-	ABLAC_28750	TGTACCTT	TGTACCTT	
	3383962	3385279	-	ABLAC_32510	ACTAGGTA	ACTAGGTA	
	3522314	3523631	+	ABLAC_33840	ATGACGAT	ATGACGAT	
	3938086	3939403	-	ABLAC_37690	TACCAAGA	TACCAAGA	

IS*Aba125*	137035	138061	-	ABLAC_01245	GAG	GAG	
	841363	842449	-	ABLAC_08000	ATA	ATA	
	905814	906900	-	ABLAC_08560	TTC	TTC	
	918557	919643	-	ABLAC_08680	CCA	CCA	
	1010371	1011457	-	ABLAC_09610	ATC	ATC	
	1811303	1812389	+	ABLAC_17380	TTG	TTG	
	1996714	1997800	+	ABLAC_19080	GTG	GTG	
	2003128	2004214	+	ABLAC_19130	GGG	GGG	
	2112514	2113600	+	ABLAC_20120	TTT	TTT	
	2153480	2154566	-	ABLAC_20490	TGT	TGT	
	2341630	2342716	-	ABLAC_22300	AGT	AGT	
	2529245	2530331	+	ABLAC_24290	CTT	CTT	
	2730419	2731505	+	ABLAC_26230	CTA	CTA	
	2990405	2991491	+	ABLAC_28680	ATG	ATG	

Supplementary Table S2. Antimicrobial susceptibility profile of LAC-4.

Drug class	Antimicrobial drug	MIC (μg/mL)	Breakpoint interpretation*
Penicillins	ampicillin	>256	R
	carbenicillin	>256	R
	piperacillin	256	R
Carbapenems	imipenem	≤2	S
	meropenem	4	S
Cephalosporins	cefepime	16	I
	cefotaxime	256	R
	ceftazidime	128	R
	ceftriaxone	≥ 256	R
Aminoglycosides	kanamycin	>256	R
	amikacin	32	I
	gentamicin	16	R
	tobramycin	32	R
Quinolones/Fluoroquinolones	nalidixic acid	>256	R
	ciprofloxacin	16	R
	gatifloxacin	2	S
	levofloxacin	≤ 2	S
Tetracyclines	doxycycline	0.5	S
	minocycline	≤ 0.25	S
	tetracycline	8	I
	tigecycline	≤ 0.25	S
Folate pathway inhibitor	trimethoprim	32	R
Phenicol	chloramphenicol	64	R

R, resistant; I, intermediate; S, susceptible

Supplementary Table S3. Hit genes from LAC-4 genome after BLASTp search against VFDB.

Query	VFDB_ID	E-value	Score	% Identity	% Positive	% Matching Length	Description
ABLAC_00070	VFG0526	2.00E-13	172	29	46	35	(gi:16766168) sitB - Salmonella iron transporter: fur regulated [Salmonella enterica (serovar typhimurium) LT2]
ABLAC_00220	VFG2230	3.00E-13	167	30	49	52	(gi:17987701) wbkC - GDP-mannose 4,6-dehydratase / GDP-4-amino-4,6-dideoxy-D-mannose formyltransferase [Brucella melitensis 16M] (VF0367)
ABLAC_00300	VFG1474	1.00E-05	99	27	44	72	(gi:24528006) ORF23 - putative UidR transriptional regulator [Escherichia coli 536]
ABLAC_00400	VFG1584	8.00E-08	120	20	37	93	(gi:23954267) orf50 - hypothetical protein [Escherichia coli 536]
ABLAC_00420	VFG1604	2.00E-10	145	29	43	31	(gi:23954287) orf70 - hypothetical protein [Escherichia coli 536]
ABLAC_00530	VFG0080	1.00E-07	120	25	45	59	(gi:16803037) clpE - ATP-dependent protease [Listeria monocytogenes (serovar 1/2a) EGD-e] (VF0073)
ABLAC_00560	VFG1387	3.00E-12	156	30	44	90	(gi:15607920) purC - purC [Mycobacterium tuberculosis H37Rv]
ABLAC_00580	VFG1109	8.00E-17	197	24	43	100	(gi:15641779) nanA - N-acetylneuraminate lyase, putative [Vibrio cholerae N16961]
ABLAC_00620	VFG0490	2.00E-05	96	27	44	96	(gi:16764739) orf319 - putative inner membrane protein [Salmonella enterica (serovar typhimurium) LT2]
ABLAC_00680	VFG1671	6.00E-06	105	24	45	33	(gi:28316251) orf48 - putative lysin/cadaverin transporter [Escherichia coli 536]
ABLAC_00700	VFG1661	5.00E-33	339	27	44	94	(gi:28316241) orf38 - hypothetical protein [Escherichia coli 536]
ABLAC_00990	VFG1888	6.00E-17	200	24	46	51	(gi:52842130) letS - sensory box histidine kinase/response regulator [Legionella pneumophila Philadelphia 1] (VF0262)
ABLAC_01000	VFG1563	6.00E-40	396	37	58	97	(gi:23954247) orf29 - hypothetical protein [Escherichia coli 536]
ABLAC_01020	VFG1922	1.00E-09	135	28	52	58	(gi:15792655) ptmA - putative oxidoreductase (flagellin modification) [Campylobacter jejuni NCTC 11168] (VF0114)
ABLAC_01040	VFG1604	3.00E-12	160	26	48	43	(gi:23954287) orf70 - hypothetical protein [Escherichia coli 536]
ABLAC_01240	VFG2077	4.00E-32	335	24	40	58	(gi:15595289) vgrG1 - hypothetical protein [Pseudomonas aeruginosa PAO1] (VF0334)
ABLAC_01390	VFG0934	1.00E-11	153	29	46	71	(gi:26246575) entA - 2,3-dihydro-2,3-dihydroxybenzoate dehydrogenase [Escherichia coli CFT073] (VF0228)
ABLAC_01420	VFG0173	8.00E-28	293	28	45	96	(gi:15599413) phzS - probable FAD-dependent monooxygenase [Pseudomonas aeruginosa PAO1] (VF0100)
ABLAC_01530	VFG1415	6.00E-27	285	29	48	79	(gi:15610623) lipF - lipF [Mycobacterium tuberculosis H37Rv] (VF0307)
ABLAC_01580	VFG0163	1.00E-24	268	23	40	107	(gi:15597594) fpvA - ferripyoverdine receptor [Pseudomonas aeruginosa PAO1] (VF0094)
ABLAC_01740	VFG0164	1.00E-34	354	24	42	92	(gi:15599417) fptA - Fe(III)-pyochelin receptor precursor [Pseudomonas aeruginosa PAO1] (VF0095)
ABLAC_01780	VFG2362	3.00E-24	264	28	44	104	(gi:123443280) manB - phosphomannomutase [Yersinia enterocolitica 8081] (VF0392)
ABLAC_01850	VFG0141	2.00E-76	712	38	52	95	(gi:15600181) waaA - lipopolysaccharide core biosynthesis protein WaaP [Pseudomonas aeruginosa PAO1] (VF0085)
ABLAC_01900	VFG1817	7.00E-21	236	24	39	83	(gi:15609521) mbtA - mbtA [Mycobacterium tuberculosis H37Rv] (VF0299)
ABLAC_01910	VFG0933	3.00E-06	103	25	44	105	(gi:26246574) entB - Isochorismatase [Escherichia coli CFT073] (VF0228)
ABLAC_01950	VFG1746	5.00E-20	223	29	52	90	(gi:16120592) YPO0255 - putative two-component response regulator [Yersinia pestis CO92]
ABLAC_01970	VFG2045	3.00E-23	258	26	47	28	(gi:33592925) bvgS - virulence sensor protein [Bordetella pertussis Tohama I] (VF0336)
ABLAC_02060	VFG0869	1.00E-09	141	38	58	9	(gi:34148232) aatC - AatC ATB binding protein of ABC transporter [Escherichia coli 042] (VF0215)
ABLAC_02070	VFG1474	2.00E-07	114	29	48	49	(gi:24528006) ORF23 - putative UidR transriptional regulator [Escherichia coli 536]
ABLAC_02080	VFG1408	1.00E-10	144	26	45	56	(gi:15610077) mas - mas [Mycobacterium tuberculosis H37Rv] (VF0309)
ABLAC_02140	VFG0576	8.00E-39	385	64	79	57	(gi:16767506) ssb - ssDNA-binding protein controls activity of RecBCD nuclease [Salmonella enterica (serovar typhimurium) LT2]
ABLAC_02210	VFG0082	1.00E-58	560	30	50	99	(gi:15640836) aldA - aldehyde dehydrogenase [Vibrio cholerae N16961]
ABLAC_02220	VFG1584	2.00E-08	125	23	42	86	(gi:23954267) orf50 - hypothetical protein [Escherichia coli 536]
ABLAC_02230	VFG0572	1.00E-44	436	43	62	93	(gi:16767045) slsA - putative inner membrane protein [Salmonella enterica (serovar typhimurium) LT2]
ABLAC_02300	VFG1584	1.00E-07	118	27	48	41	(gi:23954267) orf50 - hypothetical protein [Escherichia coli 536]
ABLAC_02460	VFG0564	3.00E-08	125	23	47	30	(gi:16767037) sugR - ATP binding protein [Salmonella enterica (serovar typhimurium) LT2]
ABLAC_02480	VFG0537	2.00E-14	176	38	54	35	(gi:16766181) hilD - regulatory helix-turn-helix proteins, araC family [Salmonella enterica (serovar typhimurium) LT2] (VF0116)
ABLAC_02640	VFG1266	3.00E-16	195	24	47	43	(gi:15599418) pchI - probable ATP-binding component of ABC transporter [Pseudomonas aeruginosa PAO1] (VF0095)
ABLAC_02730	VFG0472	4.00E-20	228	28	47	57	(gi:16767541) basS - sensory kinase in two-component regulatory system with BasR [Salmonella enterica (serovar typhimurium) LT2]
ABLAC_02740	VFG1390	3.00E-41	407	43	61	89	(gi:15608121) mprA - hypothetical protein Rv0981 [Mycobacterium tuberculosis H37Rv] (VF0298)
ABLAC_02760	VFG2301	5.00E-37	375	38	57	30	(gi:17233497) mig-5 - putative carbonic anhydrase [Salmonella enterica (serovar typhimurium) LT2] (VF0396)
ABLAC_02770	VFG0934	6.00E-11	147	25	41	82	(gi:26246575) entA - 2,3-dihydro-2,3-dihydroxybenzoate dehydrogenase [Escherichia coli CFT073] (VF0228)
ABLAC_02790	VFG1042	2.00E-14	179	31	48	31	(gi:15808722) fecE - ATP-binding protein FecE [Shigella flexneri (serotype 2a) YSH6000]
ABLAC_02920	VFG0480	8.00E-13	165	25	42	80	(gi:16764729) orf48 - putative amino acid permease [Salmonella enterica (serovar typhimurium) LT2]
ABLAC_02950	VFG0839	4.00E-05	97	53	71	9	(gi:15801498) paa - Paa [Escherichia coli O157:H7 EDL933] (VF0194)
ABLAC_03050	VFG1222	2.00E-93	860	51	68	101	(gi:15600237) pilM - type 4 fimbrial biogenesis protein PilM [Pseudomonas aeruginosa PAO1] (VF0082)
ABLAC_03060	VFG1221	1.00E-29	306	40	55	75	(gi:15600236) pilN - type 4 fimbrial biogenesis protein PilN [Pseudomonas aeruginosa PAO1] (VF0082)
ABLAC_03070	VFG1220	3.00E-38	381	41	56	77	(gi:15600235) pilO - type 4 fimbrial biogenesis protein PilO [Pseudomonas aeruginosa PAO1] (VF0082)
ABLAC_03080	VFG1219	9.00E-25	263	36	56	89	(gi:15600234) pilP - type 4 fimbrial biogenesis protein PilP [Pseudomonas aeruginosa PAO1] (VF0082)
ABLAC_03090	VFG1218	1.00E-132	1198	39	55	99	(gi:15600233) pilQ - type 4 fimbrial biogenesis protein PilQ [Pseudomonas aeruginosa PAO1] (VF0082)
ABLAC_03200	VFG0221	3.00E-18	206	34	47	109	(gi:15675966) pilE - pilin PilE [Neisseria meningitidis MC58 (serogroup B)] (VF0075)
ABLAC_03260	VFG1826	5.00E-70	660	28	48	88	(gi:15609720) relA - relA [Mycobacterium tuberculosis H37Rv] (VF0287)
ABLAC_03340	VFG1210	4.00E-07	120	31	46	11	(gi:15599750) pilY1 - type 4 fimbrial biogenesis protein PilY1 [Pseudomonas aeruginosa PAO1] (VF0082)
ABLAC_03410	VFG1316	1.00E-07	118	27	38	60	(gi:21282026) geh - glycerol ester hydrolase [Staphylococcus aureus MW2] (VF0012)
ABLAC_03570	VFG0463	2.00E-39	390	51	67	79	(gi:16764404) sodCI - Gifsy-2 prophage: superoxide dismutase precursor (Cu-Zn) [Salmonella enterica (serovar typhimurium) LT2] (VF0109)
ABLAC_03650	VFG0480	1.00E-07	120	23	39	84	(gi:16764729) orf48 - putative amino acid permease [Salmonella enterica (serovar typhimurium) LT2]
ABLAC_03710	VFG0082	3.00E-37	376	30	44	97	(gi:15640836) aldA - aldehyde dehydrogenase [Vibrio cholerae N16961]
ABLAC_03790	VFG1584	6.00E-09	130	25	45	60	(gi:23954267) orf50 - hypothetical protein [Escherichia coli 536]
ABLAC_04020	VFG0344	3.00E-30	313	34	56	85	(gi:16272073) hitC - iron(III) ABC transporter, ATP-binding protein [Haemophilus influenzae Rd] (VF0268)
ABLAC_04740	VFG1234	3.00E-08	123	27	52	48	(gi:15595613) chpD - probable transcriptional regulator [Pseudomonas aeruginosa PAO1] (VF0082)
ABLAC_05170	VFG1443	1.00E-13	166	39	55	60	(gi:7188818) ompA - outer membrane protein A [Escherichia coli] (VF0236)
ABLAC_05350	VFG0574	1.00E-24	269	23	41	78	(gi:16767047) mgtB - Mg<up>2+</up> transport protein [Salmonella enterica (serovar typhimurium) LT2] (VF0106)
ABLAC_05360	VFG0595	4.00E-42	417	31	51	67	(gi:16764453) copS - Copper resistance; histidine kinase [Salmonella enterica (serovar typhimurium) LT2]

ABLAC_05370	VFG0596	5.00E-68	637	54	73	98	(gi:16764454) copR - Copper resistance; transcriptional regulatory protein [Salmonella enterica (serovar typhimurium) LT2]
ABLAC_05450	VFG1859	2.00E-74	697	33	48	98	(gi:52842863) feoB - ferrous iron transporter B [Legionella pneumophila Philadelphia 1] (VF0160)
ABLAC_05530	VFG1738	8.00E-08	115	37	59	48	(gi:26249452) c3617 - Unknown in putative ISEc8 [Escherichia coli CFT073]
ABLAC_05540	VFG1665	1.00E-10	138	35	52	91	(gi:28316245) pB171ORF50 - ORF50 protein of pB171 [Escherichia coli 536]
ABLAC_05550	VFG0793	3.00E-85	791	38	54	89	(gi:15804210) Z5098 - unknown protein encoded by ISEc8 within prophage CP-933L [Escherichia coli O157:H7 EDL933]
ABLAC_05570	VFG0783	2.00E-45	445	28	47	98	(gi:15804200) intL - putative integrase for prophage 933L and the LEE pathogenicity island [Escherichia coli O157:H7 EDL933]
ABLAC_05670	VFG1119	5.00E-22	241	43	65	48	(gi:15641789) radC - DNA repair protein RadC, putative [Vibrio cholerae N16961]
ABLAC_05770	VFG2045	2.00E-28	299	35	51	58	(gi:33592925) bvgS - virulence sensor protein [Bordetella pertussis Tohama I] (VF0336)
ABLAC_05820	VFG1040	2.00E-17	201	27	44	91	(gi:15808720) shf - putative protein Shf [Shigella flexneri (serotype 2a) YSH6000]
ABLAC_05830	VFG1936	4.00E-24	259	30	51	103	(gi:15792460) Cj1135 - putative two-domain glycosyltransferase [Campylobacter jejuni NCTC 11168] (VF0326)
ABLAC_05840	VFG0428	1.00E-11	153	30	45	50	(gi:16763118) tviE - Vi polysaccharide biosynthesis protein TviE, Glycosyl transferases group 1 [Salmonella enterica (serovar typhi) CT18] (VF0101)
ABLAC_05870	VFG1373	1.00E-98	904	47	67	97	(gi:15900286) cps4I - UDP-N-acetylglucosamine-2-epimerase [Streptococcus pneumoniae TIGR4] (VF0144)
ABLAC_05880	VFG1344	2.00E-19	214	42	59	98	(gi:22537325) cpsM - polysaccharide biosynthesis protein CpsM(V) [Streptococcus agalactiae 2603V/R] (VF0274)
ABLAC_05900	VFG1668	1.00E-17	208	25	50	43	(gi:28316248) orf45 - putative lysil-tRNA synthetase LysU [Escherichia coli 536]
ABLAC_05920	VFG0348	1.00E-07	122	27	38	38	(gi:16272220) hxuC - heme-hemopexin utilization protein C [Haemophilus influenzae Rd] (VF0269)
ABLAC_06000	VFG0493	7.00E-09	131	21	38	56	(gi:16764742) ssrA - Secretion system regulator:Sensor component [Salmonella enterica (serovar typhimurium) LT2]
ABLAC_06010	VFG1563	3.00E-38	380	38	58	101	(gi:23954247) orf29 - hypothetical protein [Escherichia coli 536]
ABLAC_06070	VFG1731	4.00E-14	178	26	50	33	(gi:26249445) c3610 - Putative receptor [Escherichia coli CFT073]
ABLAC_06200	VFG1404	5.00E-21	232	32	56	75	(gi:15609565) ahpC - ahpC [Mycobacterium tuberculosis H37Rv] (VF0306)
ABLAC_06310	VFG1507	5.00E-10	140	34	59	27	(gi:24528037) ORF56 - AraC-like transcriptional regulator [Escherichia coli 536]
ABLAC_06380	VFG1587	3.00E-07	109	27	50	89	(gi:23954270) orf53 - hypothetical protein [Escherichia coli 536]
ABLAC_06420	VFG1931	7.00E-20	224	31	46	65	(gi:15792793) cadF - outer membrane fibronectin-binding protein [Campylobacter jejuni NCTC 11168] (VF0322)
ABLAC_06540	VFG0917	3.00E-08	127	23	37	75	(gi:26250130) chuA - Outer membrane heme/hemoglobin receptor [Escherichia coli CFT073] (VF0227)
ABLAC_06570	VFG1883	9.00E-10	136	39	56	31	(gi:52842845) enhC - enhanced entry protein EnhC [Legionella pneumophila Philadelphia 1] (VF0163)
ABLAC_06660	VFG0124	2.00E-05	100	23	42	33	(gi:15598738) Alg44 - alginate biosynthesis protein Alg8 [Pseudomonas aeruginosa PAO1] (VF0091)
ABLAC_06680	VFG1225	3.00E-55	523	74	89	100	(gi:15595605) pilG - twitching motility protein PilG [Pseudomonas aeruginosa PAO1] (VF0082)
ABLAC_06690	VFG1226	2.00E-44	431	64	81	99	(gi:15595606) pilH - twitching motility protein PilH [Pseudomonas aeruginosa PAO1] (VF0082)
ABLAC_06700	VFG1227	1.00E-35	356	38	58	97	(gi:15595607) pilI - twitching motility protein PilI [Pseudomonas aeruginosa PAO1] (VF0082)
ABLAC_06710	VFG1228	1.00E-159	1431	50	66	93	(gi:15595608) pilJ - twitching motility protein PilJ [Pseudomonas aeruginosa PAO1] (VF0082)
ABLAC_06720	VFG1225	6.00E-22	249	36	66	8	(gi:15595605) pilG - twitching motility protein PilG [Pseudomonas aeruginosa PAO1] (VF0082)
ABLAC_06830	VFG1036	5.00E-09	132	25	44	47	(gi:15808716) tetA(B) - tetracycline resistance protein TetA(B) [Shigella flexneri (serotype 2a) YSH6000]
ABLAC_07110	VFG0537	1.00E-08	128	37	56	23	(gi:16766181) hilD - regulatory helix-turn-helix proteins, araC family [Salmonella enterica (serovar typhimurium) LT2] (VF0116)
ABLAC_07150	VFG0399	1.00E-10	143	29	55	35	(gi:16082737) virF - putative thermoregulatory protein [Yersinia pestis CO92] (VF0140)
ABLAC_07160	VFG1408	1.00E-20	231	24	40	97	(gi:15610077) mas - mas [Mycobacterium tuberculosis H37Rv] (VF0309)
ABLAC_07300	VFG0367	5.00E-18	209	29	41	53	(gi:16122163) ybtX - putative signal transducer [Yersinia pestis CO92] (VF0136)
ABLAC_07320	VFG1440	6.00E-18	210	22	40	87	(gi:4741821) ibeC - membrane protein YijP [Escherichia coli] (VF0237)
ABLAC_07330	VFG0473	6.00E-35	352	36	56	98	(gi:16767542) basR - response regulator in two-component regulatory system with BasS [Salmonella enterica (serovar typhimurium) LT2]
ABLAC_07340	VFG0472	4.00E-24	262	28	48	63	(gi:16767541) basS - sensory kinase in two-component regulatory system with BasR [Salmonella enterica (serovar typhimurium) LT2]
ABLAC_07370	VFG1584	5.00E-13	165	34	53	44	(gi:23954267) orf50 - hypothetical protein [Escherichia coli 536]
ABLAC_07440	VFG0934	4.00E-07	113	23	42	70	(gi:26246575) entA - 2,3-dihydro-2,3-dihydroxybenzoate dehydrogenase [Escherichia coli CFT073] (VF0228)
ABLAC_07480	VFG1738	8.00E-08	115	37	59	48	(gi:26249452) c3617 - Unknown in putative ISEc8 [Escherichia coli CFT073]
ABLAC_07490	VFG1665	1.00E-10	138	35	52	91	(gi:28316245) pB171ORF50 - ORF50 protein of pB171 [Escherichia coli 536]
ABLAC_07500	VFG0793	3.00E-85	791	38	54	89	(gi:15804210) Z5098 - unknown protein encoded by ISEc8 within prophage CP-933L [Escherichia coli O157:H7 EDL933]
ABLAC_07510	VFG1441	3.00E-57	548	33	52	85	(gi:4835717) ibeB - IbeB [Escherichia coli] (VF0237)
ABLAC_07580	VFG0793	3.00E-85	791	38	54	89	(gi:15804210) Z5098 - unknown protein encoded by ISEc8 within prophage CP-933L [Escherichia coli O157:H7 EDL933]
ABLAC_07590	VFG1665	1.00E-10	138	35	52	91	(gi:28316245) pB171ORF50 - ORF50 protein of pB171 [Escherichia coli 536]
ABLAC_07600	VFG1666	5.00E-08	116	37	56	49	(gi:28316246) orf43 - hypothetical protein [Escherichia coli 536]
ABLAC_07720	VFG0480	4.00E-46	452	30	50	98	(gi:16764729) orf48 - putative amino acid permease [Salmonella enterica (serovar typhimurium) LT2]
ABLAC_07730	VFG1593	5.00E-47	459	33	48	102	(gi:23954276) orf59 - hypothetical protein [Escherichia coli 536]
ABLAC_07770	VFG1405	7.00E-85	788	58	80	38	(gi:15609840) sigA - sigA [Mycobacterium tuberculosis H37Rv] (VF0257)
ABLAC_08030	VFG0680	2.00E-05	101	24	38	56	(gi:6470207) capA - CapA [Bacillus anthracis] (VF0141)
ABLAC_08040	VFG0680	9.00E-11	143	30	46	72	(gi:6470207) capA - CapA [Bacillus anthracis] (VF0141)
ABLAC_08100	VFG1037	4.00E-09	129	23	44	83	(gi:15808717) tetC - putative transcriptional regulator TetC [Shigella flexneri (serotype 2a) YSH6000]
ABLAC_08330	VFG1855	0	1749	66	79	96	(gi:52840925) htpB - Hsp60, 60K heat shock protein HtpB [Legionella pneumophila Philadelphia 1] (VF0159)
ABLAC_08460	VFG0930	2.00E-75	709	35	52	42	(gi:26246565) entF - Enterobactin synthetase component F [Escherichia coli CFT073] (VF0228)
ABLAC_08480	VFG1284	1.00E-05	98	25	42	73	(gi:21284314) icaR - ica operon transcriptional regulator [Staphylococcus aureus MW2] (VF0014)
ABLAC_08500	VFG0934	6.00E-11	146	27	45	71	(gi:26246575) entA - 2,3-dihydro-2,3-dihydroxybenzoate dehydrogenase [Escherichia coli CFT073] (VF0228)
ABLAC_08510	VFG1284	3.00E-05	95	34	50	27	(gi:21284314) icaR - ica operon transcriptional regulator [Staphylococcus aureus MW2] (VF0014)
ABLAC_08590	VFG1668	2.00E-30	314	29	48	103	(gi:28316248) orf45 - putative lysil-tRNA synthetase LysU [Escherichia coli 536]
ABLAC_08710	VFG0872	1.00E-16	196	28	47	57	(gi:26251200) fimE - Type 1 fimbriae Regulatory protein fimE [Escherichia coli CFT073] (VF0221)
ABLAC_08770	VFG0670	3.00E-64	607	42	58	92	(gi:24111748) gtrB - bactoprenol glucosyl transferase [Shigella flexneri (serotype 2a) 301] (VF0124)
ABLAC_08890	VFG1206	3.00E-24	260	35	49	93	(gi:15677997) fbpC - iron(III) ABC transporter, ATP-binding protein [Neisseria meningitidis MC58 (serogroup B)] (VF0272)
ABLAC_08910	VFG0793	3.00E-85	791	38	54	89	(gi:15804210) Z5098 - unknown protein encoded by ISEc8 within prophage CP-933L [Escherichia coli O157:H7 EDL933]
ABLAC_08920	VFG1665	1.00E-10	138	35	52	91	(gi:28316245) pB171ORF50 - ORF50 protein of pB171 [Escherichia coli 536]
ABLAC_08930	VFG1738	8.00E-08	115	37	59	48	(gi:26249452) c3617 - Unknown in putative ISEc8 [Escherichia coli CFT073]
ABLAC_08940	VFG2025	4.00E-15	183	24	49	86	(gi:15792459) htrB - putative lipid A biosynthesis lauroyl acyltransferase [Campylobacter jejuni NCTC 11168] (VF0326)

ABLAC_08970	VFG2230	2.00E-09	132	24	44	80	(gi:17987701) wbkC - GDP-mannose 4,6-dehydratase / GDP-4-amino-4,6-dideoxy-D-mannose formyltransferase [Brucella melitensis 16M] (VF0367)
ABLAC_09060	VFG1392	5.00E-07	111	27	38	83	(gi:15608361) sigE - sigE [Mycobacterium tuberculosis H37Rv] (VF0295)
ABLAC_09100	VFG1931	5.00E-12	154	33	52	53	(gi:15792793) cadF - outer membrane fibronectin-binding protein [Campylobacter jejuni NCTC 11168] (VF0322)
ABLAC_09270	VFG1404	7.00E-09	125	29	48	86	(gi:15609565) ahpC - ahpC [Mycobacterium tuberculosis H37Rv] (VF0306)
ABLAC_09340	VFG1584	2.00E-07	116	34	54	35	(gi:23954267) orf50 - hypothetical protein [Escherichia coli 536]
ABLAC_09350	VFG1206	2.00E-46	454	36	55	84	(gi:15677997) fbpC - iron(III) ABC transporter, ATP-binding protein [Neisseria meningitidis MC58 (serogroup B)] (VF0272)
ABLAC_09360	VFG0249	1.00E-09	136	24	42	72	(gi:15677998) fbpB - iron(III) ABC transporter, permease protein [Neisseria meningitidis MC58 (serogroup B)] (VF0272)
ABLAC_09370	VFG0249	1.00E-04	92	21	38	96	(gi:15677998) fbpB - iron(III) ABC transporter, permease protein [Neisseria meningitidis MC58 (serogroup B)] (VF0272)
ABLAC_09400	VFG0105	9.00E-05	94	47	61	12	(gi:15640858) acfC - accessory colonization factor AcfC [Vibrio cholerae N16961] (VF0127)
ABLAC_09570	VFG0934	2.00E-13	167	25	41	97	(gi:26246575) entA - 2,3-dihydro-2,3-dihydroxybenzoate dehydrogenase [Escherichia coli CFT073] (VF0228)
ABLAC_09640	VFG1965	2.00E-07	117	27	47	45	(gi:15792755) Cj1437c - putative aminotransferase [Campylobacter jejuni NCTC 11168] (VF0323)
ABLAC_09760	VFG1963	5.00E-09	128	26	51	66	(gi:15792753) Cj1435c - hypothetical protein Cj1435c [Campylobacter jejuni NCTC 11168] (VF0323)
ABLAC_09820	VFG1312	9.00E-77	715	39	58	98	(gi:21281868) cap8P - capsular polysaccharide synthesis enzyme Cap8P [Staphylococcus aureus MW2] (VF0003)
ABLAC_09930	VFG1285	4.00E-26	279	27	47	75	(gi:21284315) icaA - intercellular adhesion protein A [Staphylococcus aureus MW2] (VF0014)
ABLAC_10070	VFG1584	2.00E-05	98	28	46	58	(gi:23954267) orf50 - hypothetical protein [Escherichia coli 536]
ABLAC_10250	VFG1037	3.00E-06	104	30	55	37	(gi:15808717) tetC - putative transcriptional regulator TetC [Shigella flexneri (serotype 2a) YSH6000]
ABLAC_10290	VFG0082	1.00E-64	612	34	50	95	(gi:15640836) aldA - aldehyde dehydrogenase [Vibrio cholerae N16961]
ABLAC_10320	VFG1671	4.00E-06	107	22	47	30	(gi:28316251) orf48 - putative lysin/cadaverin transporter [Escherichia coli 536]
ABLAC_10360	VFG0344	7.00E-25	267	27	53	82	(gi:16272073) hitC - iron(III) ABC transporter, ATP-binding protein [Haemophilus influenzae Rd] (VF0268)
ABLAC_10380	VFG0581	8.00E-15	184	26	43	72	(gi:16767511) STM4261 - putative inner membrane protein [Salmonella enterica (serovar typhimurium) LT2]
ABLAC_10500	VFG1584	5.00E-12	156	24	42	98	(gi:23954267) orf50 - hypothetical protein [Escherichia coli 536]
ABLAC_10620	VFG1693	2.00E-14	173	30	46	96	(gi:26249391) int - Prophage P4 integrase [Escherichia coli CFT073]
ABLAC_10700	VFG0165	4.00E-57	548	32	50	69	(gi:15599421) pchF - pyochelin synthetase [Pseudomonas aeruginosa PAO1] (VF0095)
ABLAC_10710	VFG1821	1.00E-48	476	26	43	72	(gi:15609517) mbtE - mbtE [Mycobacterium tuberculosis H37Rv] (VF0299)
ABLAC_10730	VFG0922	2.00E-15	186	24	41	91	(gi:26250139) chuU - Putative permease of iron compound ABC transport system [Escherichia coli CFT073] (VF0227)
ABLAC_10750	VFG1042	2.00E-46	451	40	60	93	(gi:15808722) fecE - ATP-binding protein FecE [Shigella flexneri (serotype 2a) YSH6000]
ABLAC_10760	VFG1045	2.00E-08	125	34	52	30	(gi:15808725) fecB - FecB [Shigella flexneri (serotype 2a) YSH6000]
ABLAC_10770	VFG1046	3.00E-11	153	24	40	38	(gi:15808726) orf30 - unknown [Shigella flexneri (serotype 2a) YSH6000]
ABLAC_10780	VFG0618	8.00E-55	527	29	48	98	(gi:24114947) iucD - lysine 6-monooxygenase IucD [imported] [Shigella flexneri (serotype 2a) 301] (VF0123)
ABLAC_10790	VFG1403	2.00E-65	621	35	50	47	(gi:15609520) mbtB - mbtB [Mycobacterium tuberculosis H37Rv] (VF0299)
ABLAC_10800	VFG0932	1.00E-172	1540	56	72	99	(gi:26246573) entE - Enterobactin synthetase component E [Escherichia coli CFT073] (VF0228)
ABLAC_10810	VFG0933	4.00E-76	709	48	65	97	(gi:26246574) entB - Isochorismatase [Escherichia coli CFT073] (VF0228)
ABLAC_10840	VFG0365	7.00E-21	235	22	40	84	(gi:16122161) ybtP - lipoprotein inner membrane ABC-transporter [Yersinia pestis CO92] (VF0136)
ABLAC_10850	VFG0366	2.00E-38	386	42	61	38	(gi:16122162) ybtQ - inner membrane ABC-transporter YbtQ [Yersinia pestis CO92] (VF0136)
ABLAC_10860	VFG0169	3.00E-17	200	23	44	93	(gi:15599425) pchC - pyochelin biosynthetic protein PchC [Pseudomonas aeruginosa PAO1] (VF0095)
ABLAC_10870	VFG0929	1.00E-08	127	33	45	50	(gi:26246560) entD - 4'-phosphopantetheinyl transferase entD [Escherichia coli CFT073] (VF0228)
ABLAC_10880	VFG0931	7.00E-66	622	39	57	86	(gi:26246572) entC - Isochorismate synthase entC [Escherichia coli CFT073] (VF0228)
ABLAC_11030	VFG1398	1.00E-43	429	39	55	77	(gi:15609329) trpD - trpD [Mycobacterium tuberculosis H37Rv]
ABLAC_11040	VFG1731	7.00E-12	160	31	50	16	(gi:26249445) c3610 - Putative receptor [Escherichia coli CFT073]
ABLAC_11090	VFG1857	1.00E-17	207	21	41	98	(gi:52840983) iraAB - IraAB [Legionella pneumophila Philadelphia 1] (VF0293)
ABLAC_11100	VFG1399	1.00E-126	1146	49	65	100	(gi:15609357) glnA1 - glnA1 [Mycobacterium tuberculosis H37Rv]
ABLAC_11160	VFG1867	2.00E-64	606	59	70	94	(gi:52843161) sodB - superoxide dismutase [Legionella pneumophila Philadelphia 1] (VF0169)
ABLAC_11210	VFG0584	3.00E-34	353	32	56	25	(gi:16767514) yjcC - putative diguanylate cyclase/phosphodiesterase [Salmonella enterica (serovar typhimurium) LT2]
ABLAC_11390	VFG1234	1.00E-22	247	27	44	90	(gi:15595613) chpD - probable transcriptional regulator [Pseudomonas aeruginosa PAO1] (VF0082)
ABLAC_11490	VFG0526	6.00E-24	259	28	49	76	(gi:16766168) sitB - Salmonella iron transporter: fur regulated [Salmonella enterica (serovar typhimurium) LT2]
ABLAC_11520	VFG1460	3.00E-06	106	30	49	45	(gi:24527992) y1094 - ORF B protein [Escherichia coli 536]
ABLAC_11810	VFG0564	6.00E-12	156	46	61	15	(gi:16767037) sugR - ATP binding protein [Salmonella enterica (serovar typhimurium) LT2]
ABLAC_11920	VFG1441	4.00E-37	374	27	44	95	(gi:4835717) ibeB - IbeB [Escherichia coli] (VF0237)
ABLAC_11960	VFG1584	7.00E-13	164	23	43	75	(gi:23954267) orf50 - hypothetical protein [Escherichia coli 536]
ABLAC_12000	VFG1206	3.00E-31	322	36	52	93	(gi:15677997) fbpC - iron(III) ABC transporter, ATP-binding protein [Neisseria meningitidis MC58 (serogroup B)] (VF0272)
ABLAC_12010	VFG1586	2.00E-14	171	33	54	94	(gi:23954269) orf52 - hypothetical protein [Escherichia coli 536]
ABLAC_12040	VFG1604	7.00E-08	122	28	46	32	(gi:23954287) orf70 - hypothetical protein [Escherichia coli 536]
ABLAC_12090	VFG0182	1.00E-98	907	47	67	65	(gi:15598299) xcpR - general secretion pathway protein E [Pseudomonas aeruginosa PAO1] (VF0084)
ABLAC_12110	VFG0473	2.00E-43	426	41	60	98	(gi:16767542) basR - response regulator in two-component regulatory system with BasS [Salmonella enterica (serovar typhimurium) LT2]
ABLAC_12120	VFG0472	3.00E-18	212	26	44	64	(gi:16767541) basS - sensory kinase in two-component regulatory system with BasR [Salmonella enterica (serovar typhimurium) LT2]
ABLAC_12220	VFG1651	1.00E-19	222	32	50	55	(gi:14594881) iroE - IroE protein [Escherichia coli 536]
ABLAC_12330	VFG0524	7.00E-34	347	33	51	51	(gi:16766165) fhlA - formate hydrogen-lyase transcriptional activator for fdhF, hyc and hyp operons (EBP family) [Salmonella enterica (serovar typhimurium) LT2]
ABLAC_12400	VFG1866	5.00E-24	259	27	49	93	(gi:52841515) rpoS - stationary phase specific sigma factor RpoS [Legionella pneumophila Philadelphia 1] (VF0166)
ABLAC_12630	VFG2060	9.00E-08	118	30	41	75	(gi:15595272) ppkA - serine/threonine protein kinase PpkA [Pseudomonas aeruginosa PAO1] (VF0334)
ABLAC_12660	VFG0082	3.00E-48	472	29	47	85	(gi:15640836) aldA - aldehyde dehydrogenase [Vibrio cholerae N16961]
ABLAC_12670	VFG0480	3.00E-06	108	20	40	69	(gi:16764729) orf48 - putative amino acid permease [Salmonella enterica (serovar typhimurium) LT2]
ABLAC_12720	VFG0670	8.00E-06	101	37	58	27	(gi:24111748) gtrB - bactoprenol glucosyl transferase [Shigella flexneri (serotype 2a) 301] (VF0124)
ABLAC_12770	VFG1234	2.00E-18	212	22	41	97	(gi:15595613) chpD - probable transcriptional regulator [Pseudomonas aeruginosa PAO1] (VF0082)
ABLAC_12900	VFG0596	3.00E-57	544	49	67	98	(gi:16764454) copR - Copper resistance; transcriptional regulatory protein [Salmonella enterica (serovar typhimurium) LT2]
ABLAC_12910	VFG0595	2.00E-35	359	26	48	99	(gi:16764453) copS - Copper resistance; histidine kinase [Salmonella enterica (serovar typhimurium) LT2]
ABLAC_12960	VFG1382	3.00E-32	332	30	47	66	(gi:15607611) pcaA - umaA2 [Mycobacterium tuberculosis H37Rv] (VF0285)

ABLAC_12980	VFG1382	2.00E-15	186	24	39	78	(gi:15607611) pcaA - umaA2 [Mycobacterium tuberculosis H37Rv] (VF0285)
ABLAC_12990	VFG0595	1.00E-09	138	30	50	36	(gi:16764453) copS - Copper resistance; histidine kinase [Salmonella enterica (serovar typhimurium) LT2]
ABLAC_13000	VFG0596	4.00E-34	345	36	58	99	(gi:16764454) copR - Copper resistance; transcriptional regulatory protein [Salmonella enterica (serovar typhimurium) LT2]
ABLAC_13040	VFG1024	5.00E-11	146	28	49	59	(gi:15808704) orf8 - unknown [Shigella flexneri (serotype 2a) YSH6000]
ABLAC_13140	VFG1557	5.00E-35	355	36	57	69	(gi:23954241) hlyB - HlyB protein [Escherichia coli 536]
ABLAC_13420	VFG1040	9.00E-18	209	27	47	40	(gi:15808720) shf - putative protein Shf [Shigella flexneri (serotype 2a) YSH6000]
ABLAC_13430	VFG1285	9.00E-85	785	39	60	100	(gi:21284315) icaA - intercellular adhesion protein A [Staphylococcus aureus MW2] (VF0014)
ABLAC_13490	VFG1584	7.00E-19	215	29	49	61	(gi:23954267) orf50 - hypothetical protein [Escherichia coli 536]
ABLAC_13530	VFG1507	6.00E-08	121	29	60	26	(gi:24528037) ORF56 - AraC-like transcriptional regulator [Escherichia coli 536]
ABLAC_13540	VFG0934	3.00E-12	157	28	47	83	(gi:26246575) entA - 2,3-dihydro-2,3-dihydroxybenzoate dehydrogenase [Escherichia coli CFT073] (VF0228)
ABLAC_13570	VFG1821	1.00E-29	311	23	43	95	(gi:15609517) mbtE - mbtE [Mycobacterium tuberculosis H37Rv] (VF0299)
ABLAC_13650	VFG0574	1.00E-12	165	22	40	66	(gi:16767047) mgtB - Mg<up>2+</up> transport protein [Salmonella enterica (serovar typhimurium) LT2] (VF0106)
ABLAC_13670	VFG1701	2.00E-17	206	27	46	28	(gi:26249399) c3564 - Hypothetical protein [Escherichia coli CFT073]
ABLAC_13680	VFG1390	6.00E-34	344	36	59	93	(gi:15608121) mprA - hypothetical protein Rv0981 [Mycobacterium tuberculosis H37Rv] (VF0298)
ABLAC_13710	VFG1399	3.00E-20	229	28	42	61	(gi:15609357) glnA1 - glnA1 [Mycobacterium tuberculosis H37Rv]
ABLAC_13850	VFG1584	2.00E-10	141	29	45	60	(gi:23954267) orf50 - hypothetical protein [Escherichia coli 536]
ABLAC_13990	VFG1027	3.00E-17	200	26	44	73	(gi:15808707) oxa-1 - beta-lactamase Oxa-1 [Shigella flexneri (serotype 2a) YSH6000]
ABLAC_14120	VFG0082	1.00E-178	1596	57	71	100	(gi:15640836) aldA - aldehyde dehydrogenase [Vibrio cholerae N16961]
ABLAC_14150	VFG1593	2.00E-54	522	34	53	94	(gi:23954276) orf59 - hypothetical protein [Escherichia coli 536]
ABLAC_14240	VFG1146	4.00E-13	170	23	40	76	(gi:581263) hifC - membrane anchor [Haemophilus influenzae AM30] (VF0036)
ABLAC_14340	VFG1037	4.00E-06	102	31	68	30	(gi:15808717) tetC - putative transcriptional regulator TetC [Shigella flexneri (serotype 2a) YSH6000]
ABLAC_14350	VFG0619	8.00E-60	572	27	45	92	(gi:24114948) iutA - receptor-like protein iutA [imported] [Shigella flexneri (serotype 2a) 301] (VF0123)
ABLAC_14400	VFG1738	8.00E-08	115	37	59	48	(gi:26249452) c3617 - Unknown in putative ISEc8 [Escherichia coli CFT073]
ABLAC_14410	VFG1665	1.00E-10	138	35	52	91	(gi:28316245) pB171ORF50 - ORF50 protein of pB171 [Escherichia coli 536]
ABLAC_14420	VFG0793	3.00E-85	791	38	54	89	(gi:15804210) Z5098 - unknown protein encoded by ISEc8 within prophage CP-933L [Escherichia coli O157:H7 EDL933]
ABLAC_14450	VFG0163	1.00E-118	1075	34	52	101	(gi:15597594) fpvA - ferripyoverdine receptor [Pseudomonas aeruginosa PAO1] (VF0094)
ABLAC_14500	VFG1653	2.00E-06	111	24	42	46	(gi:28316233) iroC - ABC transport protein [Escherichia coli 536]
ABLAC_14560	VFG0574	0	2332	52	68	96	(gi:16767047) mgtB - Mg<up>2+</up> transport protein [Salmonella enterica (serovar typhimurium) LT2] (VF0106)
ABLAC_14570	VFG0575	7.00E-31	318	41	55	73	(gi:16767048) mgtC - Mg<up>2+</up> transport protein [Salmonella enterica (serovar typhimurium) LT2] (VF0106)
ABLAC_14620	VFG0441	2.00E-05	99	36	53	25	(gi:17233493) spvR - Salmonella plasmid virulence: regulation of spv operon, lysR family [Salmonella enterica (serovar typhimurium) LT2] (VF0107)
ABLAC_14650	VFG0934	2.00E-09	135	24	39	54	(gi:26246575) entA - 2,3-dihydro-2,3-dihydroxybenzoate dehydrogenase [Escherichia coli CFT073] (VF0228)
ABLAC_14730	VFG0157	1.00E-113	1037	36	50	104	(gi:15596041) plcH - hemolytic phospholipase C precursor [Pseudomonas aeruginosa PAO1] (VF0092)
ABLAC_14750	VFG1593	3.00E-44	435	31	48	103	(gi:23954276) orf59 - hypothetical protein [Escherichia coli 536]
ABLAC_14820	VFG2162	4.00E-06	106	22	45	73	(gi:16804106) bsh - bile salt hydrolase [Listeria monocytogenes (serovar 1/2a) EGD-e] (VF0350)
ABLAC_14860	VFG1037	1.00E-06	108	28	55	46	(gi:15808717) tetC - putative transcriptional regulator TetC [Shigella flexneri (serotype 2a) YSH6000]
ABLAC_15690	VFG1965	6.00E-07	114	27	46	41	(gi:15792755) Cj1437c - putative aminotransferase [Campylobacter jejuni NCTC 11168] (VF0323)
ABLAC_15750	VFG2361	5.00E-48	467	34	52	100	(gi:123443279) galE - UDP-glucose 4-epimerase [Yersinia enterocolitica 8081] (VF0392)
ABLAC_15880	VFG1214	2.00E-82	764	36	56	96	(gi:15599743) pilR - two-component response regulator PilR [Pseudomonas aeruginosa PAO1] (VF0082)
ABLAC_15890	VFG1213	2.00E-10	143	25	44	58	(gi:15599742) pilS - two-component sensor PilS [Pseudomonas aeruginosa PAO1] (VF0082)
ABLAC_16070	VFG0783	7.00E-17	200	27	40	95	(gi:15804200) intL - putative integrase for prophage 933L and the LEE pathogenicity island [Escherichia coli O157:H7 EDL933]
ABLAC_16690	VFG0783	1.00E-68	645	35	54	95	(gi:15804200) intL - putative integrase for prophage 933L and the LEE pathogenicity island [Escherichia coli O157:H7 EDL933]
ABLAC_16700	VFG1313	2.00E-08	124	51	77	23	(gi:21281813) spa - Immunoglobulin G binding protein A precursor [Staphylococcus aureus MW2] (VF0017)
ABLAC_16760	VFG1109	2.00E-12	161	25	42	99	(gi:15641779) nanA - N-acetylneuraminate lyase, putative [Vibrio cholerae N16961]
ABLAC_16770	VFG1584	2.00E-08	125	32	47	53	(gi:23954267) orf50 - hypothetical protein [Escherichia coli 536]
ABLAC_16780	VFG1049	1.00E-27	287	35	60	93	(gi:15808729) fecI - FecI [Shigella flexneri (serotype 2a) YSH6000]
ABLAC_16790	VFG1048	9.00E-21	232	31	50	64	(gi:15808728) fecR - FecR [Shigella flexneri (serotype 2a) YSH6000]
ABLAC_16800	VFG1047	1.00E-07	123	34	52	8	(gi:15808727) fecA - FecA [Shigella flexneri (serotype 2a) YSH6000]
ABLAC_16890	VFG1671	1.00E-08	128	23	37	86	(gi:28316251) orf48 - putative lysin/cadaverin transporter [Escherichia coli 536]
ABLAC_16940	VFG0082	2.00E-94	869	38	58	98	(gi:15640836) aldA - aldehyde dehydrogenase [Vibrio cholerae N16961]
ABLAC_17010	VFG1661	3.00E-10	141	23	44	65	(gi:28316241) orf38 - hypothetical protein [Escherichia coli 536]
ABLAC_17040	VFG1864	9.00E-13	157	39	50	85	(gi:52841028) mip - macrophage infectivity potentiator (Mip) [Legionella pneumophila Philadelphia 1] (VF0153)
ABLAC_17050	VFG1287	2.00E-17	206	24	46	34	(gi:21284317) icaB - intercellular adhesion protein B [Staphylococcus aureus MW2] (VF0014)
ABLAC_17060	VFG1285	2.00E-89	825	39	62	97	(gi:21284315) icaA - intercellular adhesion protein A [Staphylococcus aureus MW2] (VF0014)
ABLAC_17150	VFG0934	8.00E-18	205	28	45	100	(gi:26246575) entA - 2,3-dihydro-2,3-dihydroxybenzoate dehydrogenase [Escherichia coli CFT073] (VF0228)
ABLAC_17180	VFG1089	1.00E-06	111	26	45	49	(gi:16802879) hpt - highly similar to hexose phosphate transport protein [Listeria monocytogenes (serovar 1/2a) EGD-e] (VF0264)
ABLAC_17210	VFG0934	2.00E-18	211	27	42	100	(gi:26246575) entA - 2,3-dihydro-2,3-dihydroxybenzoate dehydrogenase [Escherichia coli CFT073] (VF0228)
ABLAC_17230	VFG0082	8.00E-82	760	34	54	99	(gi:15640836) aldA - aldehyde dehydrogenase [Vibrio cholerae N16961]
ABLAC_17300	VFG1234	6.00E-08	120	28	48	39	(gi:15595613) chpD - probable transcriptional regulator [Pseudomonas aeruginosa PAO1] (VF0082)
ABLAC_17420	VFG1582	1.00E-174	1560	66	79	98	(gi:23954265) orf48 - hypothetical protein [Escherichia coli 536]
ABLAC_17470	VFG1444	1.00E-16	199	37	56	25	(gi:9965749) aslA - putative arylsulfatase [Escherichia coli] (VF0238)
ABLAC_17500	VFG0935	0	1725	50	66	95	(gi:26247124) iroN - Siderophore receptor IroN [Escherichia coli CFT073] (VF0230)
ABLAC_17530	VFG2301	3.00E-25	268	34	54	100	(gi:17233497) mig-5 - putative carbonic anhydrase [Salmonella enterica (serovar typhimurium) LT2] (VF0396)
ABLAC_17550	VFG0869	6.00E-25	266	37	55	79	(gi:34148232) aatC - AatC ATB binding protein of ABC transporter [Escherichia coli 042] (VF0215)
ABLAC_17680	VFG1668	1.00E-155	1390	54	71	97	(gi:28316248) orf45 - putative lysil-tRNA synthetase LysU [Escherichia coli 536]
ABLAC_17770	VFG0775	2.00E-05	100	41	50	16	(gi:11527922) rorf5 - unknown [Escherichia coli E2348/69]
ABLAC_17780	VFG1381	4.00E-30	315	27	39	83	(gi:15607608) icl/aceA - aceA [Mycobacterium tuberculosis H37Rv] (VF0253)
ABLAC_17830	VFG0269	4.00E-28	288	61	75	100	(gi:15644703) ureA - urease alpha subunit (ureA) (urea amidohydrolase) [Helicobacter pylori 26695] (VF0050)

ABLAC_17840	VFG0269	9.00E-28	285	50	71	95	(gi:15644703) ureA - urease alpha subunit (ureA) (urea amidohydrolase) [Helicobacter pylori 26695] (VF0050)
ABLAC_17850	VFG0270	0	1655	56	72	100	(gi:15644702) ureB - urease beta subunit (urea amidohydrolase) (ureB) [Helicobacter pylori 26695] (VF0050)
ABLAC_17860	VFG0272	5.00E-05	92	28	52	68	(gi:15644700) ureE - urease accessory protein (ureE) [Helicobacter pylori 26695] (VF0050)
ABLAC_17870	VFG0273	2.00E-12	158	27	44	99	(gi:15644699) ureF - urease accessory protein (ureF) [Helicobacter pylori 26695] (VF0050)
ABLAC_17880	VFG0274	3.00E-73	682	60	84	96	(gi:15644698) ureG - urease accessory protein (ureG) [Helicobacter pylori 26695] (VF0050)
ABLAC_18280	VFG1440	3.00E-16	194	25	43	66	(gi:4741821) ibeC - membrane protein YijP [Escherichia coli] (VF0237)
ABLAC_18420	VFG1667	3.00E-38	383	32	53	94	(gi:28316247) orf44 - hypothetical protein [Escherichia coli 536]
ABLAC_18560	VFG1206	2.00E-26	282	33	54	45	(gi:15677997) fbpC - iron(III) ABC transporter, ATP-binding protein [Neisseria meningitidis MC58 (serogroup B)] (VF0272)
ABLAC_18580	VFG0163	2.00E-43	431	25	43	102	(gi:15597594) fpvA - ferripyoverdine receptor [Pseudomonas aeruginosa PAO1] (VF0094)
ABLAC_18650	VFG0598	1.00E-50	490	33	53	101	(gi:24114927) intC - Sai integrase [Shigella flexneri (serotype 2a) 301]
ABLAC_18670	VFG0793	3.00E-85	791	38	54	89	(gi:15804210) Z5098 - unknown protein encoded by ISEc8 within prophage CP-933L [Escherichia coli O157:H7 EDL933]
ABLAC_18680	VFG1665	1.00E-10	138	35	52	91	(gi:28316245) pB171ORF50 - ORF50 protein of pB171 [Escherichia coli 536]
ABLAC_18690	VFG1738	8.00E-08	115	37	59	48	(gi:26249452) c3617 - Unknown in putative ISEc8 [Escherichia coli CFT073]
ABLAC_18720	VFG0646	2.00E-09	134	26	42	49	(gi:24114246) SF2982 - orf, conserved hypothetical protein [Shigella flexneri (serotype 2a) 301]
ABLAC_18780	VFG1523	4.00E-21	234	30	52	71	(gi:24528052) ORF72 - hypothetical protein [Escherichia coli 536]
ABLAC_18890	VFG1105	3.00E-49	476	40	57	91	(gi:15641775) VC1772 - hypothetical protein [Vibrio cholerae N16961]
ABLAC_18990	VFG0627	9.00E-13	169	24	37	22	(gi:24114229) SF2965 - putative superfamily I DNA helicases [Shigella flexneri (serotype 2a) 301]
ABLAC_19060	VFG1284	9.00E-06	101	27	46	51	(gi:21284314) icaR - ica operon transcriptional regulator [Staphylococcus aureus MW2] (VF0014)
ABLAC_19090	VFG2077	3.00E-29	309	24	42	52	(gi:15595289) vgrG1 - hypothetical protein [Pseudomonas aeruginosa PAO1] (VF0334)
ABLAC_19150	VFG1537	8.00E-18	211	25	45	27	(gi:23954221) orf2 - hypothetical protein [Escherichia coli 536]
ABLAC_19170	VFG1738	8.00E-08	115	37	59	48	(gi:26249452) c3617 - Unknown in putative ISEc8 [Escherichia coli CFT073]
ABLAC_19180	VFG1665	1.00E-10	138	35	52	91	(gi:28316245) pB171ORF50 - ORF50 protein of pB171 [Escherichia coli 536]
ABLAC_19190	VFG0793	8.00E-54	518	36	51	80	(gi:15804210) Z5098 - unknown protein encoded by ISEc8 within prophage CP-933L [Escherichia coli O157:H7 EDL933]
ABLAC_19200	VFG0634	2.00E-22	241	44	61	93	(gi:24114236) SF2972 - orf, conserved hypothetical protein [Shigella flexneri (serotype 2a) 301]
ABLAC_19210	VFG1671	3.00E-05	100	23	44	30	(gi:28316251) orf48 - putative lysin/cadaverin transporter [Escherichia coli 536]
ABLAC_19230	VFG0480	1.00E-39	396	27	46	100	(gi:16764729) orf48 - putative amino acid permease [Salmonella enterica (serovar typhimurium) LT2]
ABLAC_19270	VFG0082	3.00E-72	677	32	50	102	(gi:15640836) aldA - aldehyde dehydrogenase [Vibrio cholerae N16961]
ABLAC_19350	VFG0082	7.00E-25	269	25	42	73	(gi:15640836) aldA - aldehyde dehydrogenase [Vibrio cholerae N16961]
ABLAC_19360	VFG0763	5.00E-07	113	24	50	34	(gi:6009400) perA/bfpT - transcriptional regulator BfpT [Escherichia coli B171] (VF0190)
ABLAC_19400	VFG2097	4.00E-08	124	21	40	81	(gi:52840784) phtA - major facilitator family transporter [Legionella pneumophila Philadelphia 1] (VF0337)
ABLAC_19430	VFG0082	1.00E-17	208	26	40	87	(gi:15640836) aldA - aldehyde dehydrogenase [Vibrio cholerae N16961]
ABLAC_19500	VFG1820	9.00E-05	97	21	36	80	(gi:15609516) mbtF - mbtF [Mycobacterium tuberculosis H37Rv] (VF0299)
ABLAC_19510	VFG0082	3.00E-50	488	28	48	100	(gi:15640836) aldA - aldehyde dehydrogenase [Vibrio cholerae N16961]
ABLAC_19590	VFG2057	4.00E-06	105	31	40	40	(gi:15595269) PA0071 - hypothetical protein [Pseudomonas aeruginosa PAO1] (VF0334)
ABLAC_19600	VFG1444	3.00E-18	212	25	37	79	(gi:9965749) aslA - putative arylsulfatase [Escherichia coli] (VF0238)
ABLAC_19660	VFG1415	9.00E-10	136	24	39	73	(gi:15610623) lipF - lipF [Mycobacterium tuberculosis H37Rv] (VF0307)
ABLAC_19670	VFG1922	3.00E-10	141	27	49	72	(gi:15792655) ptmA - putative oxidoreductase (flagellin modification) [Campylobacter jejuni NCTC 11168] (VF0114)
ABLAC_19710	VFG0937	3.00E-05	100	27	50	20	(gi:26249459) iucD - IucD protein [Escherichia coli CFT073] (VF0229)
ABLAC_19720	VFG0934	7.00E-14	172	29	47	68	(gi:26246575) entA - 2,3-dihydro-2,3-dihydroxybenzoate dehydrogenase [Escherichia coli CFT073] (VF0228)
ABLAC_19780	VFG0934	2.00E-14	177	30	48	68	(gi:26246575) entA - 2,3-dihydro-2,3-dihydroxybenzoate dehydrogenase [Escherichia coli CFT073] (VF0228)
ABLAC_19860	VFG1886	3.00E-17	194	63	81	65	(gi:52842308) csrA - carbon storage regulator RsmA [Legionella pneumophila Philadelphia 1] (VF0261)
ABLAC_20020	VFG0079	0	1747	44	61	100	(gi:16802278) clpC - endopeptidase Clp ATP-binding chain C [Listeria monocytogenes (serovar 1/2a) EGD-e] (VF0072)
ABLAC_20090	VFG1388	9.00E-17	199	40	58	24	(gi:15608039) ompA - ompA [Mycobacterium tuberculosis H37Rv]
ABLAC_20240	VFG1404	9.00E-30	307	38	56	83	(gi:15609565) ahpC - ahpC [Mycobacterium tuberculosis H37Rv] (VF0306)
ABLAC_20300	VFG1922	8.00E-12	154	23	41	104	(gi:15792655) ptmA - putative oxidoreductase (flagellin modification) [Campylobacter jejuni NCTC 11168] (VF0114)
ABLAC_20350	VFG0574	8.00E-21	236	23	39	72	(gi:16767047) mgtB - Mg<up>2+</up> transport protein [Salmonella enterica (serovar typhimurium) LT2] (VF0106)
ABLAC_20510	VFG1384	3.00E-21	235	26	46	101	(gi:15607641) proC - proC [Mycobacterium tuberculosis H37Rv]
ABLAC_20660	VFG1971	4.00E-67	632	44	60	92	(gi:15792761) kpsF - KpsF protein [Campylobacter jejuni NCTC 11168] (VF0323)
ABLAC_20700	VFG1206	5.00E-23	250	29	48	91	(gi:15677997) fbpC - iron(III) ABC transporter, ATP-binding protein [Neisseria meningitidis MC58 (serogroup B)] (VF0272)
ABLAC_20710	VFG0579	6.00E-05	97	26	44	37	(gi:16767509) STM4259 - putative ABC exporter outer membrane component homolog [Salmonella enterica (serovar typhimurium) LT2]
ABLAC_20730	VFG1557	2.00E-57	551	23	45	97	(gi:23954241) hlyB - HlyB protein [Escherichia coli 536]
ABLAC_20740	VFG1270	2.00E-28	298	28	46	95	(gi:33591936) cyaD - cyclolysin secretion protein [Bordetella pertussis Tohama I] (VF0028)
ABLAC_20760	VFG1306	2.00E-05	97	39	64	26	(gi:21281862) cap8J - capsular polysaccharide synthesis enzyme Cap8J [Staphylococcus aureus MW2] (VF0003)
ABLAC_20820	VFG0562	0	2106	49	66	98	(gi:16766215) mutS - methyl-directed mismatch repair, recognize exocyclic adducts of guanosine [Salmonella enterica (serovar typhimurium) LT2]
ABLAC_20870	VFG0584	4.00E-26	277	31	51	98	(gi:16767514) yjcC - putative diguanylate cyclase/phosphodiesterase [Salmonella enterica (serovar typhimurium) LT2]
ABLAC_20880	VFG2025	9.00E-09	128	23	39	91	(gi:15792459) htrB - putative lipid A biosynthesis lauroyl acyltransferase [Campylobacter jejuni NCTC 11168] (VF0326)
ABLAC_20890	VFG1284	6.00E-06	102	28	46	54	(gi:21284314) icaR - ica operon transcriptional regulator [Staphylococcus aureus MW2] (VF0014)
ABLAC_21000	VFG1584	7.00E-11	146	25	47	56	(gi:23954267) orf50 - hypothetical protein [Escherichia coli 536]
ABLAC_21070	VFG1883	2.00E-22	245	28	44	94	(gi:52842845) enhC - enhanced entry protein EnhC [Legionella pneumophila Philadelphia 1] (VF0163)
ABLAC_21100	VFG1037	2.00E-06	106	34	65	32	(gi:15808717) tetC - putative transcriptional regulator TetC [Shigella flexneri (serotype 2a) YSH6000]
ABLAC_21230	VFG0344	7.00E-36	362	34	56	78	(gi:16272073) hitC - iron(III) ABC transporter, ATP-binding protein [Haemophilus influenzae Rd] (VF0268)
ABLAC_21260	VFG2077	5.00E-33	342	23	40	72	(gi:15595289) vgrG1 - hypothetical protein [Pseudomonas aeruginosa PAO1] (VF0334)
ABLAC_21330	VFG1883	3.00E-16	193	28	51	63	(gi:52842845) enhC - enhanced entry protein EnhC [Legionella pneumophila Philadelphia 1] (VF0163)
ABLAC_21350	VFG2082	1.00E-36	370	24	43	100	(gi:15600885) VCA0114 - hypothetical protein [Vibrio cholerae N16961] (VF0335)
ABLAC_21360	VFG2064	1.00E-11	152	25	48	54	(gi:15595276) PA0078 - hypothetical protein [Pseudomonas aeruginosa PAO1] (VF0334)
ABLAC_21390	VFG1667	6.00E-07	112	28	46	49	(gi:28316247) orf44 - hypothetical protein [Escherichia coli 536]

ABLAC_21460	VFG0586	5.00E-50	479	65	74	98	(gi:16767516) soxR - redox-sensing transcriptional activator SoxR, contains iron-sulfur center for redox-sensing (MerR family) [Salmonella enterica (serovar typhimurium) LT2]
ABLAC_21480	VFG1109	9.00E-06	102	25	42	52	(gi:15641779) nanA - N-acetylneuraminate lyase, putative [Vibrio cholerae N16961]
ABLAC_21550	VFG1234	2.00E-08	124	25	45	54	(gi:15595613) chpD - probable transcriptional regulator [Pseudomonas aeruginosa PAO1] (VF0082)
ABLAC_21570	VFG0082	5.00E-08	124	22	38	58	(gi:15640836) aldA - aldehyde dehydrogenase [Vibrio cholerae N16961]
ABLAC_21600	VFG0082	3.00E-12	162	24	42	46	(gi:15640836) aldA - aldehyde dehydrogenase [Vibrio cholerae N16961]
ABLAC_21690	VFG1408	4.00E-05	97	60	68	8	(gi:15610077) mas - mas [Mycobacterium tuberculosis H37Rv] (VF0309)
ABLAC_21720	VFG1306	2.00E-07	114	46	67	27	(gi:21281862) cap8J - capsular polysaccharide synthesis enzyme Cap8J [Staphylococcus aureus MW2] (VF0003)
ABLAC_21770	VFG1584	1.00E-15	188	27	46	63	(gi:23954267) orf50 - hypothetical protein [Escherichia coli 536]
ABLAC_21840	VFG1206	4.00E-56	537	41	56	71	(gi:15677997) fbpC - iron(III) ABC transporter, ATP-binding protein [Neisseria meningitidis MC58 (serogroup B)] (VF0272)
ABLAC_21850	VFG0343	3.00E-09	134	18	43	71	(gi:16272072) hitB - iron(III) ABC transporter, permease protein [Haemophilus influenzae Rd] (VF0268)
ABLAC_21990	VFG0934	6.00E-20	223	27	43	96	(gi:26246575) entA - 2,3-dihydro-2,3-dihydroxybenzoate dehydrogenase [Escherichia coli CFT073] (VF0228)
ABLAC_22110	VFG1584	8.00E-13	163	23	43	79	(gi:23954267) orf50 - hypothetical protein [Escherichia coli 536]
ABLAC_22170	VFG1474	1.00E-05	99	35	52	35	(gi:24528006) ORF23 - putative UidR transriptional regulator [Escherichia coli 536]
ABLAC_22180	VFG0932	2.00E-37	379	25	44	93	(gi:26246573) entE - Enterobactin synthetase component E [Escherichia coli CFT073] (VF0228)
ABLAC_22230	VFG1584	4.00E-15	183	31	51	53	(gi:23954267) orf50 - hypothetical protein [Escherichia coli 536]
ABLAC_22340	VFG0934	1.00E-17	204	27	47	88	(gi:26246575) entA - 2,3-dihydro-2,3-dihydroxybenzoate dehydrogenase [Escherichia coli CFT073] (VF0228)
ABLAC_22420	VFG2045	8.00E-33	341	29	44	34	(gi:33592925) bvgS - virulence sensor protein [Bordetella pertussis Tohama I] (VF0336)
ABLAC_22430	VFG1226	1.00E-05	94	30	50	83	(gi:15595606) pilH - twitching motility protein PilH [Pseudomonas aeruginosa PAO1] (VF0082)
ABLAC_22440	VFG0151	2.00E-05	97	38	56	25	(gi:15596627) lasR - transcriptional regulator LasR [Pseudomonas aeruginosa PAO1] (VF0093)
ABLAC_22480	VFG1206	3.00E-41	406	37	57	97	(gi:15677997) fbpC - iron(III) ABC transporter, ATP-binding protein [Neisseria meningitidis MC58 (serogroup B)] (VF0272)
ABLAC_22490	VFG1983	6.00E-22	241	32	50	72	(gi:15792250) peb1A - probable ABC-type amino-acid transporter periplasmic solute-binding protein [Campylobacter jejuni NCTC 11168] (VF0327)
ABLAC_22500	VFG1983	7.00E-19	215	36	59	44	(gi:15792250) peb1A - probable ABC-type amino-acid transporter periplasmic solute-binding protein [Campylobacter jejuni NCTC 11168] (VF0327)
ABLAC_22510	VFG1584	2.00E-16	195	24	43	98	(gi:23954267) orf50 - hypothetical protein [Escherichia coli 536]
ABLAC_22570	VFG0934	1.00E-14	177	28	42	104	(gi:26246575) entA - 2,3-dihydro-2,3-dihydroxybenzoate dehydrogenase [Escherichia coli CFT073] (VF0228)
ABLAC_22580	VFG1584	3.00E-12	158	23	40	98	(gi:23954267) orf50 - hypothetical protein [Escherichia coli 536]
ABLAC_22610	VFG1584	2.00E-05	98	29	43	42	(gi:23954267) orf50 - hypothetical protein [Escherichia coli 536]
ABLAC_22740	VFG0362	6.00E-14	173	24	41	93	(gi:16122158) irp1 - yersiniabactin biosynthetic protein HMWP1 (high molecular weight protein 1) [Yersinia pestis CO92] (VF0136)
ABLAC_22780	VFG1604	2.00E-08	127	22	45	56	(gi:23954287) orf70 - hypothetical protein [Escherichia coli 536]
ABLAC_22890	VFG0441	5.00E-07	113	31	56	31	(gi:17233493) spvR - Salmonella plasmid virulence: regulation of spv operon, lysR family [Salmonella enterica (serovar typhimurium) LT2] (VF0107)
ABLAC_22950	VFG0037	2.00E-05	98	30	43	48	(gi:33591350) bplB - probable acetyltransferase [Bordetella pertussis Tohama I] (VF0033)
ABLAC_23000	VFG1604	2.00E-09	137	21	46	51	(gi:23954287) orf70 - hypothetical protein [Escherichia coli 536]
ABLAC_23030	VFG1234	2.00E-13	168	37	67	29	(gi:15595613) chpD - probable transcriptional regulator [Pseudomonas aeruginosa PAO1] (VF0082)
ABLAC_23050	VFG1965	3.00E-09	133	25	46	66	(gi:15792755) Cj1437c - putative aminotransferase [Campylobacter jejuni NCTC 11168] (VF0323)
ABLAC_23170	VFG0344	1.00E-21	240	26	49	73	(gi:16272073) hitC - iron(III) ABC transporter, ATP-binding protein [Haemophilus influenzae Rd] (VF0268)
ABLAC_23240	VFG1983	4.00E-19	217	29	45	91	(gi:15792250) peb1A - probable ABC-type amino-acid transporter periplasmic solute-binding protein [Campylobacter jejuni NCTC 11168] (VF0327)
ABLAC_23270	VFG1206	1.00E-33	341	34	57	90	(gi:15677997) fbpC - iron(III) ABC transporter, ATP-binding protein [Neisseria meningitidis MC58 (serogroup B)] (VF0272)
ABLAC_23290	VFG1738	8.00E-08	115	37	59	48	(gi:26249452) c3617 - Unknown in putative ISEc8 [Escherichia coli CFT073]
ABLAC_23300	VFG1665	1.00E-10	138	35	52	91	(gi:28316245) pB171ORF50 - ORF50 protein of pB171 [Escherichia coli 536]
ABLAC_23310	VFG0793	3.00E-85	791	38	54	89	(gi:15804210) Z5098 - unknown protein encoded by ISEc8 within prophage CP-933L [Escherichia coli O157:H7 EDL933]
ABLAC_23350	VFG1037	1.00E-06	107	30	51	50	(gi:15808717) tetC - putative transcriptional regulator TetC [Shigella flexneri (serotype 2a) YSH6000]
ABLAC_23380	VFG1584	2.00E-07	117	24	44	79	(gi:23954267) orf50 - hypothetical protein [Escherichia coli 536]
ABLAC_23430	VFG1584	1.00E-13	170	31	51	50	(gi:23954267) orf50 - hypothetical protein [Escherichia coli 536]
ABLAC_23460	VFG0009	3.00E-09	132	28	46	43	(gi:33592930) fimD - fimbrial adhesin [Bordetella pertussis Tohama I] (VF0031)
ABLAC_23470	VFG1467	2.00E-32	331	32	51	94	(gi:24527999) ORF16 - putative F17-like fimbrial usher [Escherichia coli 536]
ABLAC_23480	VFG1467	4.00E-96	884	34	53	93	(gi:24527999) ORF16 - putative F17-like fimbrial usher [Escherichia coli 536]
ABLAC_23490	VFG0007	2.00E-49	478	41	62	93	(gi:33592928) fimB - chaperone protein [Bordetella pertussis Tohama I] (VF0031)
ABLAC_23500	VFG1469	1.00E-23	253	39	51	97	(gi:24528001) ORF18 - putative F17-like fimbrial subunit [Escherichia coli 536]
ABLAC_23600	VFG1027	2.00E-21	235	28	49	80	(gi:15808707) oxa-1 - beta-lactamase Oxa-1 [Shigella flexneri (serotype 2a) YSH6000]
ABLAC_23770	VFG0082	1.00E-38	392	29	46	36	(gi:15640836) aldA - aldehyde dehydrogenase [Vibrio cholerae N16961]
ABLAC_23830	VFG0537	6.00E-12	156	35	57	29	(gi:16766181) hilD - regulatory helix-turn-helix proteins, araC family [Salmonella enterica (serovar typhimurium) LT2] (VF0116)
ABLAC_23850	VFG1269	5.00E-57	547	31	49	88	(gi:33591935) cyaB - cyclolysin secretion ATP-binding protein [Bordetella pertussis Tohama I] (VF0028)
ABLAC_24000	VFG1951	3.00E-15	182	25	47	96	(gi:15792741) Cj1423c - putative sugar-phosphate nucleotidyltransferase [Campylobacter jejuni NCTC 11168] (VF0323)
ABLAC_24020	VFG1368	2.00E-06	107	22	47	56	(gi:15900278) cps4D - capsular polysaccharide biosynthesis protein Cps4D [Streptococcus pneumoniae TIGR4] (VF0144)
ABLAC_24060	VFG1269	5.00E-75	702	34	54	87	(gi:33591935) cyaB - cyclolysin secretion ATP-binding protein [Bordetella pertussis Tohama I] (VF0028)
ABLAC_24080	VFG1335	3.00E-09	131	33	49	41	(gi:22537316) neuA - CMP-N-acetylneuraminic acid synthetase NeuA [Streptococcus agalactiae 2603V/R] (VF0274)
ABLAC_24150	VFG0177	3.00E-11	148	30	50	61	(gi:15598294) xcpW - general secretion pathway protein J [Pseudomonas aeruginosa PAO1] (VF0084)
ABLAC_24160	VFG0176	4.00E-34	347	34	52	102	(gi:15598293) xcpX - general secretion pathway protein K [Pseudomonas aeruginosa PAO1] (VF0084)
ABLAC_24390	VFG1047	7.00E-14	177	34	53	14	(gi:15808727) fecA - FecA [Shigella flexneri (serotype 2a) YSH6000]
ABLAC_24450	VFG0344	6.00E-24	262	29	49	45	(gi:16272073) hitC - iron(III) ABC transporter, ATP-binding protein [Haemophilus influenzae Rd] (VF0268)
ABLAC_24710	VFG0208	9.00E-10	137	30	54	26	(gi:15596910) exsA - transcriptional regulator ExsA [Pseudomonas aeruginosa PAO1] (VF0083)
ABLAC_24780	VFG0940	9.00E-42	415	27	46	81	(gi:26249462) iucA - IucA protein [Escherichia coli CFT073] (VF0229)
ABLAC_24790	VFG0937	3.00E-65	617	32	51	93	(gi:26249459) iucD - IucD protein [Escherichia coli CFT073] (VF0229)
ABLAC_24810	VFG0938	2.00E-51	500	28	47	92	(gi:26249460) iucC - IucC protein [Escherichia coli CFT073] (VF0229)

ABLAC_24820	VFG0938	8.00E-11	149	24	44	35	(gi:26249460) iucC - IucC protein [Escherichia coli CFT073] (VF0229)
ABLAC_24850	VFG0619	2.00E-88	819	30	49	96	(gi:24114948) iutA - receptor-like protein iutA [imported] [Shigella flexneri (serotype 2a) 301] (VF0123)
ABLAC_24890	VFG1082	3.00E-11	148	27	41	81	(gi:14626625) iucB - IucB [Shigella boydii 0-1392]
ABLAC_24980	VFG1630	4.00E-76	709	43	61	89	(gi:14594865) orf7 - hypothetical protein [Escherichia coli 536]
ABLAC_25030	VFG0163	4.00E-82	764	30	51	97	(gi:15597594) fpvA - ferripyoverdine receptor [Pseudomonas aeruginosa PAO1] (VF0094)
ABLAC_25040	VFG0323	4.00E-24	261	25	46	76	(gi:15645486) lpxB - lipid A disaccharide synthetase (lpxB) [Helicobacter pylori 26695] (VF0056)
ABLAC_25070	VFG0344	2.00E-22	248	32	54	38	(gi:16272073) hitC - iron(III) ABC transporter, ATP-binding protein [Haemophilus influenzae Rd] (VF0268)
ABLAC_25130	VFG0250	7.00E-05	95	29	43	41	(gi:15677862) porB - major outer membrane protein PIB [Neisseria meningitidis MC58 (serogroup B)] (VF0081)
ABLAC_25240	VFG1738	8.00E-08	115	37	59	48	(gi:26249452) c3617 - Unknown in putative ISEc8 [Escherichia coli CFT073]
ABLAC_25250	VFG1665	1.00E-10	138	35	52	91	(gi:28316245) pB171ORF50 - ORF50 protein of pB171 [Escherichia coli 536]
ABLAC_25260	VFG0793	3.00E-85	791	38	54	89	(gi:15804210) Z5098 - unknown protein encoded by ISEc8 within prophage CP-933L [Escherichia coli O157:H7 EDL933]
ABLAC_25500	VFG0934	1.00E-21	239	30	45	97	(gi:26246575) entA - 2,3-dihydro-2,3-dihydroxybenzoate dehydrogenase [Escherichia coli CFT073] (VF0228)
ABLAC_25530	VFG1214	1.00E-62	594	41	60	73	(gi:15599743) pilR - two-component response regulator PilR [Pseudomonas aeruginosa PAO1] (VF0082)
ABLAC_25590	VFG1038	1.00E-12	161	30	52	43	(gi:15808718) tetD - putative transcriptional regulator TetD [Shigella flexneri (serotype 2a) YSH6000]
ABLAC_25680	VFG1206	2.00E-35	357	36	57	83	(gi:15677997) fbpC - iron(III) ABC transporter, ATP-binding protein [Neisseria meningitidis MC58 (serogroup B)] (VF0272)
ABLAC_25720	VFG0163	4.00E-11	152	19	40	103	(gi:15597594) fpvA - ferripyoverdine receptor [Pseudomonas aeruginosa PAO1] (VF0094)
ABLAC_25760	VFG1023	7.00E-29	301	25	50	97	(gi:21450882) orf7 - putative transcriptional regulator [Shigella flexneri (serotype 2a) YSH6000]
ABLAC_25770	VFG0775	8.00E-06	102	33	53	20	(gi:11527922) rorf5 - unknown [Escherichia coli E2348/69]
ABLAC_25820	VFG1584	3.00E-12	157	31	52	61	(gi:23954267) orf50 - hypothetical protein [Escherichia coli 536]
ABLAC_25850	VFG0934	9.00E-17	196	26	42	99	(gi:26246575) entA - 2,3-dihydro-2,3-dihydroxybenzoate dehydrogenase [Escherichia coli CFT073] (VF0228)
ABLAC_25860	VFG0167	3.00E-07	116	31	63	23	(gi:15599423) pchR - transcriptional regulator PchR [Pseudomonas aeruginosa PAO1] (VF0095)
ABLAC_25900	VFG0934	1.00E-20	229	29	45	97	(gi:26246575) entA - 2,3-dihydro-2,3-dihydroxybenzoate dehydrogenase [Escherichia coli CFT073] (VF0228)
ABLAC_25940	VFG0167	3.00E-09	133	33	58	23	(gi:15599423) pchR - transcriptional regulator PchR [Pseudomonas aeruginosa PAO1] (VF0095)
ABLAC_26010	VFG0344	4.00E-21	232	38	56	71	(gi:16272073) hitC - iron(III) ABC transporter, ATP-binding protein [Haemophilus influenzae Rd] (VF0268)
ABLAC_26020	VFG0934	4.00E-41	406	45	63	74	(gi:26246575) entA - 2,3-dihydro-2,3-dihydroxybenzoate dehydrogenase [Escherichia coli CFT073] (VF0228)
ABLAC_26030	VFG0933	2.00E-63	597	50	70	98	(gi:26246574) entB - Isochorismatase [Escherichia coli CFT073] (VF0228)
ABLAC_26040	VFG0478	6.00E-08	116	26	52	93	(gi:16764063) fur - transcriptional repressor of iron-responsive genes (Fur family) (ferric uptake regulator) [Salmonella enterica (serovar typhimurium) LT2] (VF0113)
ABLAC_26170	VFG0793	3.00E-85	791	38	54	89	(gi:15804210) Z5098 - unknown protein encoded by ISEc8 within prophage CP-933L [Escherichia coli O157:H7 EDL933]
ABLAC_26180	VFG1665	1.00E-10	138	35	52	91	(gi:28316245) pB171ORF50 - ORF50 protein of pB171 [Escherichia coli 536]
ABLAC_26190	VFG1738	8.00E-08	115	37	59	48	(gi:26249452) c3617 - Unknown in putative ISEc8 [Escherichia coli CFT073]
ABLAC_26290	VFG0009	4.00E-14	175	33	51	41	(gi:33592930) fimD - fimbrial adhesin [Bordetella pertussis Tohama I] (VF0031)
ABLAC_26300	VFG1467	1.00E-145	1308	36	53	96	(gi:24527999) ORF16 - putative F17-like fimbrial usher [Escherichia coli 536]
ABLAC_26310	VFG1468	2.00E-45	442	41	61	95	(gi:24528000) ORF17 - putative F17-like fimbrial chaperone [Escherichia coli 536]
ABLAC_26320	VFG1469	8.00E-20	220	42	57	80	(gi:24528001) ORF18 - putative F17-like fimbrial subunit [Escherichia coli 536]
ABLAC_26430	VFG1441	8.00E-65	613	34	54	93	(gi:4835717) ibeB - IbeB [Escherichia coli] (VF0237)
ABLAC_26500	VFG1284	1.00E-06	106	25	43	75	(gi:21284314) icaR - ica operon transcriptional regulator [Staphylococcus aureus MW2] (VF0014)
ABLAC_26550	VFG1826	1.00E-08	123	31	47	81	(gi:15609720) relA - relA [Mycobacterium tuberculosis H37Rv] (VF0287)
ABLAC_26630	VFG0843	1.00E-06	109	34	54	39	(gi:3822164) hlyD - hemolysin transport protein [Escherichia coli O157:H7 EDL933] (VF0207)
ABLAC_26710	VFG1234	1.00E-05	101	30	52	24	(gi:15595613) chpD - probable transcriptional regulator [Pseudomonas aeruginosa PAO1] (VF0082)
ABLAC_26730	VFG1042	1.00E-36	367	34	52	92	(gi:15808722) fecE - ATP-binding protein FecE [Shigella flexneri (serotype 2a) YSH6000]
ABLAC_26740	VFG0922	5.00E-38	381	32	52	88	(gi:26250139) chuU - Putative permease of iron compound ABC transport system [Escherichia coli CFT073] (VF0227)
ABLAC_26770	VFG1474	2.00E-07	114	25	46	74	(gi:24528006) ORF23 - putative UidR transriptional regulator [Escherichia coli 536]
ABLAC_26790	VFG1270	3.00E-05	98	22	38	90	(gi:33591936) cyaD - cyclolysin secretion protein [Bordetella pertussis Tohama I] (VF0028)
ABLAC_26800	VFG0025	2.00E-15	187	32	47	47	(gi:33591934) cyaA - bifunctional hemolysin-adenylate cyclase precursor [Bordetella pertussis Tohama I] (VF0028)
ABLAC_26910	VFG1284	8.00E-05	92	26	41	65	(gi:21284314) icaR - ica operon transcriptional regulator [Staphylococcus aureus MW2] (VF0014)
ABLAC_26930	VFG0168	2.00E-39	395	26	43	97	(gi:15599424) pchD - pyochelin biosynthesis protein PchD [Pseudomonas aeruginosa PAO1] (VF0095)
ABLAC_26970	VFG0934	1.00E-15	188	30	44	63	(gi:26246575) entA - 2,3-dihydro-2,3-dihydroxybenzoate dehydrogenase [Escherichia coli CFT073] (VF0228)
ABLAC_26980	VFG0934	6.00E-15	180	25	40	101	(gi:26246575) entA - 2,3-dihydro-2,3-dihydroxybenzoate dehydrogenase [Escherichia coli CFT073] (VF0228)
ABLAC_26990	VFG0934	1.00E-20	229	30	44	101	(gi:26246575) entA - 2,3-dihydro-2,3-dihydroxybenzoate dehydrogenase [Escherichia coli CFT073] (VF0228)
ABLAC_27010	VFG1474	3.00E-05	95	39	54	33	(gi:24528006) ORF23 - putative UidR transriptional regulator [Escherichia coli 536]
ABLAC_27060	VFG0491	5.00E-06	100	26	52	64	(gi:16764740) orf242 - putative regulatory proteins, merR family [Salmonella enterica (serovar typhimurium) LT2]
ABLAC_27120	VFG0082	5.00E-16	193	23	40	83	(gi:15640836) aldA - aldehyde dehydrogenase [Vibrio cholerae N16961]
ABLAC_27140	VFG2097	1.00E-05	102	28	45	27	(gi:52840784) phtA - major facilitator family transporter [Legionella pneumophila Philadelphia 1] (VF0337)
ABLAC_27180	VFG1023	4.00E-08	122	26	48	47	(gi:21450882) orf7 - putative transcriptional regulator [Shigella flexneri (serotype 2a) YSH6000]
ABLAC_27270	VFG0082	5.00E-63	598	32	50	96	(gi:15640836) aldA - aldehyde dehydrogenase [Vibrio cholerae N16961]
ABLAC_27300	VFG0364	1.00E-05	101	27	52	36	(gi:16122160) ybtA - transcriptional regulator YbtA [Yersinia pestis CO92] (VF0136)
ABLAC_27330	VFG0934	2.00E-09	132	24	42	102	(gi:26246575) entA - 2,3-dihydro-2,3-dihydroxybenzoate dehydrogenase [Escherichia coli CFT073] (VF0228)
ABLAC_27410	VFG2097	1.00E-04	95	20	37	71	(gi:52840784) phtA - major facilitator family transporter [Legionella pneumophila Philadelphia 1] (VF0337)
ABLAC_27430	VFG0934	3.00E-07	114	25	45	78	(gi:26246575) entA - 2,3-dihydro-2,3-dihydroxybenzoate dehydrogenase [Escherichia coli CFT073] (VF0228)
ABLAC_27470	VFG0441	2.00E-06	108	34	51	31	(gi:17233493) spvR - Salmonella plasmid virulence: regulation of spv operon, lysR family [Salmonella enterica (serovar typhimurium) LT2] (VF0107)
ABLAC_27660	VFG0700	1.00E-19	221	30	50	100	(gi:30962648) bcs1 - bifunctional; ribulose 5-phosphate reductase; CDP-ribitol pyrophosphorylase [Haemophilus influenzae 1007] (VF0043)
ABLAC_27800	VFG1630	5.00E-77	718	47	63	88	(gi:14594865) orf7 - hypothetical protein [Escherichia coli 536]
ABLAC_27820	VFG0080	1.00E-151	1361	44	64	81	(gi:16803037) clpE - ATP-dependent protease [Listeria monocytogenes (serovar 1/2a) EGD-e] (VF0073)
ABLAC_27940	VFG0163	4.00E-35	359	24	40	102	(gi:15597594) fpvA - ferripyoverdine receptor [Pseudomonas aeruginosa PAO1] (VF0094)
ABLAC_27950	VFG0483	2.00E-06	108	25	43	71	(gi:16764732) orf408 - putative regulatory protein, deoR family [Salmonella enterica (serovar typhimurium) LT2]

ABLAC_28040	VFG1895	6.00E-14	173	26	46	62	(gi:15791456) flhF - flagellar biosynthesis protein [Campylobacter jejuni NCTC 11168] (VF0114)
ABLAC_28140	VFG1584	3.00E-13	166	23	41	103	(gi:23954267) orf50 - hypothetical protein [Escherichia coli 536]
ABLAC_28260	VFG0584	3.00E-27	291	31	51	37	(gi:16767514) yjcC - putative diguanylate cyclase/phosphodiesterase [Salmonella enterica (serovar typhimurium) LT2]
ABLAC_28310	VFG2177	8.00E-06	104	26	39	46	(gi:21693306) cylA - cytolysin activator [Enterococcus faecalis MMH594] (VF0356)
ABLAC_28330	VFG1671	1.00E-06	112	22	41	80	(gi:28316251) orf48 - putative lysin/cadaverin transporter [Escherichia coli 536]
ABLAC_28400	VFG0793	3.00E-85	791	38	54	89	(gi:15804210) Z5098 - unknown protein encoded by ISEc8 within prophage CP-933L [Escherichia coli O157:H7 EDL933]
ABLAC_28410	VFG1665	1.00E-10	138	35	52	91	(gi:28316245) pB171ORF50 - ORF50 protein of pB171 [Escherichia coli 536]
ABLAC_29480	VFG0783	1.00E-48	473	29	49	102	(gi:15804200) intL - putative integrase for prophage 933L and the LEE pathogenicity island [Escherichia coli O157:H7 EDL933]
ABLAC_29490	VFG0679	6.00E-24	263	26	40	57	(gi:6470206) dep/capD - <gamma>-glutamyltranspeptidase [Bacillus anthracis] (VF0141)
ABLAC_29620	VFG1223	1.00E-174	1558	84	92	99	(gi:15595592) pilT - twitching motility protein PilT [Pseudomonas aeruginosa PAO1] (VF0082)
ABLAC_29630	VFG1224	1.00E-153	1377	67	83	99	(gi:15595593) pilU - twitching motility protein PilU [Pseudomonas aeruginosa PAO1] (VF0082)
ABLAC_29640	VFG0478	1.00E-47	459	59	77	96	(gi:16764063) fur - transcriptional repressor of iron-responsive genes (Fur family) (ferric uptake regulator) [Salmonella enterica (serovar typhimurium) LT2] (VF0113)
ABLAC_29720	VFG2362	2.00E-25	274	23	43	98	(gi:123443280) manB - phosphomannomutase [Yersinia enterocolitica 8081] (VF0392)
ABLAC_29750	VFG1388	1.00E-20	228	44	60	48	(gi:15608039) ompA - ompA [Mycobacterium tuberculosis H37Rv]
ABLAC_29790	VFG1265	2.00E-07	116	25	45	92	(gi:15596651) fleN - flagellar synthesis regulator FleN [Pseudomonas aeruginosa PAO1] (VF0273)
ABLAC_29820	VFG1235	4.00E-05	95	27	45	75	(gi:15595614) chpE - probable chemotaxis protein [Pseudomonas aeruginosa PAO1] (VF0082)
ABLAC_29830	VFG2409	8.00E-24	263	31	52	23	(gi:21281992) essC - hypothetical protein [Staphylococcus aureus MW2] (VF0403)
ABLAC_29950	VFG1823	2.00E-17	204	28	42	71	(gi:15609519) mbtC - mbtC [Mycobacterium tuberculosis H37Rv] (VF0299)
ABLAC_30020	VFG0082	3.00E-74	695	34	53	100	(gi:15640836) aldA - aldehyde dehydrogenase [Vibrio cholerae N16961]
ABLAC_30040	VFG1415	8.00E-18	206	32	43	69	(gi:15610623) lipF - lipF [Mycobacterium tuberculosis H37Rv] (VF0307)
ABLAC_30070	VFG1584	4.00E-15	183	28	46	61	(gi:23954267) orf50 - hypothetical protein [Escherichia coli 536]
ABLAC_30100	VFG0320	7.00E-23	245	42	64	91	(gi:15646084) kdtB - lipopolysaccharide core biosynthesis protein (kdtB) [Helicobacter pylori 26695] (VF0056)
ABLAC_30240	VFG1416	1.00E-25	268	47	63	88	(gi:15610737) panD - panD [Mycobacterium tuberculosis H37Rv] (VF0319)
ABLAC_30310	VFG0542	2.00E-07	109	32	53	96	(gi:16766187) iacP - putative acyl carrier protein [Salmonella enterica (serovar typhimurium) LT2] (VF0116)
ABLAC_30320	VFG0934	8.00E-21	231	27	47	101	(gi:26246575) entA - 2,3-dihydro-2,3-dihydroxybenzoate dehydrogenase [Escherichia coli CFT073] (VF0228)
ABLAC_30330	VFG0362	5.00E-16	191	25	42	98	(gi:16122158) irp1 - yersiniabactin biosynthetic protein HMWP1 (high molecular weight protein 1) [Yersinia pestis CO92] (VF0136)
ABLAC_30840	VFG1584	1.00E-06	109	22	43	102	(gi:23954267) orf50 - hypothetical protein [Escherichia coli 536]
ABLAC_31020	VFG0493	9.00E-25	269	26	45	65	(gi:16764742) ssrA - Secretion system regulator:Sensor component [Salmonella enterica (serovar typhimurium) LT2]
ABLAC_31030	VFG0596	6.00E-30	309	34	48	98	(gi:16764454) copR - Copper resistance; transcriptional regulatory protein [Salmonella enterica (serovar typhimurium) LT2]
ABLAC_31080	VFG1474	3.00E-06	104	37	58	27	(gi:24528006) ORF23 - putative UidR transriptional regulator [Escherichia coli 536]
ABLAC_31120	VFG1584	2.00E-12	159	27	52	49	(gi:23954267) orf50 - hypothetical protein [Escherichia coli 536]
ABLAC_31150	VFG0531	3.00E-08	124	30	58	25	(gi:16766173) hilC - bacterial regulatory helix-turn-helix proteins, araC family [Salmonella enterica (serovar typhimurium) LT2] (VF0116)
ABLAC_31190	VFG1584	4.00E-10	140	25	47	57	(gi:23954267) orf50 - hypothetical protein [Escherichia coli 536]
ABLAC_31220	VFG1584	1.00E-09	136	26	44	60	(gi:23954267) orf50 - hypothetical protein [Escherichia coli 536]
ABLAC_31300	VFG1584	3.00E-26	279	28	47	94	(gi:23954267) orf50 - hypothetical protein [Escherichia coli 536]
ABLAC_31360	VFG1586	2.00E-07	110	29	54	80	(gi:23954269) orf52 - hypothetical protein [Escherichia coli 536]
ABLAC_31530	VFG0171	3.00E-17	203	25	49	44	(gi:15599427) pchA - salicylate biosynthesis isochorismate synthase [Pseudomonas aeruginosa PAO1] (VF0095)
ABLAC_31540	VFG1965	2.00E-26	282	26	50	93	(gi:15792755) Cj1437c - putative aminotransferase [Campylobacter jejuni NCTC 11168] (VF0323)
ABLAC_31670	VFG1649	1.00E-04	89	32	51	52	(gi:14594879) sfaX - SfaX protein [Escherichia coli 536]
ABLAC_31690	VFG0182	1.00E-177	1585	65	76	97	(gi:15598299) xcpR - general secretion pathway protein E [Pseudomonas aeruginosa PAO1] (VF0084)
ABLAC_31750	VFG1384	2.00E-23	253	31	46	99	(gi:15607641) proC - proC [Mycobacterium tuberculosis H37Rv]
ABLAC_31800	VFG1254	5.00E-17	200	29	48	57	(gi:15596301) fliI - flagellum-specific ATP synthase FliI [Pseudomonas aeruginosa PAO1] (VF0273)
ABLAC_31950	VFG0168	8.00E-43	424	27	44	103	(gi:15599424) pchD - pyochelin biosynthesis protein PchD [Pseudomonas aeruginosa PAO1] (VF0095)
ABLAC_31990	VFG1417	2.00E-50	486	46	56	91	(gi:15610738) panC - panC [Mycobacterium tuberculosis H37Rv] (VF0319)
ABLAC_32050	VFG0934	7.00E-10	137	25	43	70	(gi:26246575) entA - 2,3-dihydro-2,3-dihydroxybenzoate dehydrogenase [Escherichia coli CFT073] (VF0228)
ABLAC_32060	VFG1887	1.00E-155	1397	40	61	96	(gi:52841687) relA - GTP pyrophosphokinase ((p)ppGpp synthetase I) stringent stress response RelA [Legionella pneumophila Philadelphia 1] (VF0260)
ABLAC_32100	VFG1888	4.00E-83	774	28	48	89	(gi:52842130) letS - sensory box histidine kinase/response regulator [Legionella pneumophila Philadelphia 1] (VF0262)
ABLAC_32150	VFG0934	1.00E-08	127	25	42	73	(gi:26246575) entA - 2,3-dihydro-2,3-dihydroxybenzoate dehydrogenase [Escherichia coli CFT073] (VF0228)
ABLAC_32240	VFG1584	6.00E-08	121	37	60	26	(gi:23954267) orf50 - hypothetical protein [Escherichia coli 536]
ABLAC_32360	VFG1721	1.00E-06	108	23	40	88	(gi:26249435) c3600 - Hypothetical protein [Escherichia coli CFT073]
ABLAC_32400	VFG1593	8.00E-18	207	21	42	95	(gi:23954276) orf59 - hypothetical protein [Escherichia coli 536]
ABLAC_32460	VFG1408	2.00E-13	169	24	41	101	(gi:15610077) mas - mas [Mycobacterium tuberculosis H37Rv] (VF0309)
ABLAC_32470	VFG1408	1.00E-09	136	23	41	92	(gi:15610077) mas - mas [Mycobacterium tuberculosis H37Rv] (VF0309)
ABLAC_32480	VFG1584	6.00E-19	216	29	50	57	(gi:23954267) orf50 - hypothetical protein [Escherichia coli 536]
ABLAC_32490	VFG0934	2.00E-13	166	23	46	92	(gi:26246575) entA - 2,3-dihydro-2,3-dihydroxybenzoate dehydrogenase [Escherichia coli CFT073] (VF0228)
ABLAC_32550	VFG0584	3.00E-28	299	32	52	36	(gi:16767514) yjcC - putative diguanylate cyclase/phosphodiesterase [Salmonella enterica (serovar typhimurium) LT2]
ABLAC_32670	VFG0869	6.00E-24	262	34	52	27	(gi:34148232) aatC - AatC ATB binding protein of ABC transporter [Escherichia coli 042] (VF0215)
ABLAC_32680	VFG1441	3.00E-34	350	25	46	95	(gi:4835717) ibeB - IbeB [Escherichia coli] (VF0237)
ABLAC_32770	VFG1859	2.00E-06	110	32	49	25	(gi:52842863) feoB - ferrous iron transporter B [Legionella pneumophila Philadelphia 1] (VF0160)
ABLAC_32830	VFG1217	2.00E-26	280	30	54	93	(gi:15599000) pilF - type 4 fimbrial biogenesis protein PilF [Pseudomonas aeruginosa PAO1] (VF0082)
ABLAC_32880	VFG2366	4.00E-13	167	32	45	45	(gi:123443284) rfpB - putative galactosyltransferase [Yersinia enterocolitica 8081] (VF0392)
ABLAC_32940	VFG1595	2.00E-06	110	23	41	80	(gi:23954278) orf61 - hypothetical protein [Escherichia coli 536]
ABLAC_32950	VFG0082	3.00E-27	289	25	44	80	(gi:15640836) aldA - aldehyde dehydrogenase [Vibrio cholerae N16961]
ABLAC_32970	VFG1583	3.00E-68	643	37	55	95	(gi:23954266) orf49 - hypothetical protein [Escherichia coli 536]
ABLAC_33050	VFG0080	3.00E-06	108	24	39	74	(gi:16803037) clpE - ATP-dependent protease [Listeria monocytogenes (serovar 1/2a) EGD-e] (VF0073)

ABLAC_33060	VFG0077	2.00E-76	709	67	85	93	(gi:16804506) clpP - ATP-dependent Clp protease proteolytic subunit [Listeria monocytogenes (serovar 1/2a) EGD-e] (VF0074)
ABLAC_33090	VFG0793	3.00E-85	791	38	54	89	(gi:15804210) Z5098 - unknown protein encoded by ISEc8 within prophage CP-933L [Escherichia coli O157:H7 EDL933]
ABLAC_33100	VFG1665	1.00E-10	138	35	52	91	(gi:28316245) pB171ORF50 - ORF50 protein of pB171 [Escherichia coli 536]
ABLAC_33110	VFG1738	8.00E-08	115	37	59	48	(gi:26249452) c3617 - Unknown in putative ISEc8 [Escherichia coli CFT073]
ABLAC_33120	VFG0164	6.00E-11	150	26	40	40	(gi:15599417) fptA - Fe(III)-pyochelin receptor precursor [Pseudomonas aeruginosa PAO1] (VF0095)
ABLAC_33170	VFG1883	2.00E-07	113	34	55	43	(gi:52842845) enhC - enhanced entry protein EnhC [Legionella pneumophila Philadelphia 1] (VF0163)
ABLAC_33380	VFG0082	7.00E-35	355	28	44	86	(gi:15640836) aldA - aldehyde dehydrogenase [Vibrio cholerae N16961]
ABLAC_33530	VFG1408	4.00E-06	105	25	39	72	(gi:15610077) mas - mas [Mycobacterium tuberculosis H37Rv] (VF0309)
ABLAC_33550	VFG0841	1.00E-09	139	25	44	36	(gi:3822163) hlyB - hemolysin transport protein [Escherichia coli O157:H7 EDL933] (VF0207)
ABLAC_33570	VFG2025	5.00E-08	121	19	42	91	(gi:15792459) htrB - putative lipid A biosynthesis lauroyl acyltransferase [Campylobacter jejuni NCTC 11168] (VF0326)
ABLAC_33580	VFG0242	4.00E-12	157	23	44	91	(gi:15677553) rfaK - alpha-1,2-N-acetylglucosamine transferase [Neisseria meningitidis MC58 (serogroup B)] (VF0078)
ABLAC_33660	VFG0763	3.00E-08	124	31	59	22	(gi:6009400) perA/bfpT - transcriptional regulator BfpT [Escherichia coli B171] (VF0190)
ABLAC_33720	VFG1411	6.00E-37	369	40	53	96	(gi:15610124) leuD - leuD [Mycobacterium tuberculosis H37Rv]
ABLAC_33750	VFG1584	3.00E-08	123	25	48	69	(gi:23954267) orf50 - hypothetical protein [Escherichia coli 536]
ABLAC_33780	VFG1963	8.00E-09	127	27	43	85	(gi:15792753) Cj1435c - hypothetical protein Cj1435c [Campylobacter jejuni NCTC 11168] (VF0323)
ABLAC_33810	VFG1862	0	2028	54	68	98	(gi:52842598) katB - catalase-peroxidase KatB [Legionella pneumophila Philadelphia 1] (VF0168)
ABLAC_33830	VFG1023	3.00E-09	132	27	49	55	(gi:21450882) orf7 - putative transcriptional regulator [Shigella flexneri (serotype 2a) YSH6000]
ABLAC_33920	VFG1821	7.00E-16	193	22	36	91	(gi:15609517) mbtE - mbtE [Mycobacterium tuberculosis H37Rv] (VF0299)
ABLAC_33930	VFG1584	2.00E-22	245	25	47	101	(gi:23954267) orf50 - hypothetical protein [Escherichia coli 536]
ABLAC_33940	VFG1922	2.00E-06	107	21	41	107	(gi:15792655) ptmA - putative oxidoreductase (flagellin modification) [Campylobacter jejuni NCTC 11168] (VF0114)
ABLAC_33950	VFG0919	8.00E-31	319	29	47	71	(gi:26250136) chuW - Putative oxygen independent coproporphyrinogen III oxidase [Escherichia coli CFT073] (VF0227)
ABLAC_34200	VFG0180	8.00E-25	264	43	55	74	(gi:15598297) xcpT - general secretion pathway protein G [Pseudomonas aeruginosa PAO1] (VF0084)
ABLAC_34210	VFG0181	1.00E-101	926	46	66	100	(gi:15598298) xcpS - general secretion pathway protein F [Pseudomonas aeruginosa PAO1] (VF0084)
ABLAC_34420	VFG0173	3.00E-05	100	24	40	59	(gi:15599413) phzS - probable FAD-dependent monooxygenase [Pseudomonas aeruginosa PAO1] (VF0100)
ABLAC_34450	VFG0564	2.00E-08	128	31	55	24	(gi:16767037) sugR - ATP binding protein [Salmonella enterica (serovar typhimurium) LT2]
ABLAC_34580	VFG0112	0	1731	56	75	100	(gi:15599722) pilB - type 4 fimbrial biogenesis protein PilB [Pseudomonas aeruginosa PAO1] (VF0082)
ABLAC_34590	VFG0113	1.00E-133	1203	59	77	91	(gi:15599723) pilC - still frameshift type 4 fimbrial biogenesis protein PilC [Pseudomonas aeruginosa PAO1] (VF0082)
ABLAC_34600	VFG1882	7.00E-58	551	42	59	96	(gi:52841754) pilD - type 4 (IV) prepilin-like protein leader peptide processing enzyme PilD [Legionella pneumophila Philadelphia 1] (VF0155)
ABLAC_34740	VFG1234	6.00E-06	103	25	51	34	(gi:15595613) chpD - probable transcriptional regulator [Pseudomonas aeruginosa PAO1] (VF0082)
ABLAC_34860	VFG1379	4.00E-07	109	30	53	67	(gi:15607494) hspR - hspR [Mycobacterium tuberculosis H37Rv] (VF0297)
ABLAC_35080	VFG0368	4.00E-27	289	29	50	53	(gi:16122164) ybtS - putative salicylate synthetase [Yersinia pestis CO92] (VF0136)
ABLAC_35100	VFG2139	4.00E-07	112	30	52	44	(gi:15605397) CT664 - (FHA domain; homology to adenylate cyclase) [Chlamydia trachomatis D/UW-3/CX] (VF0344)
ABLAC_35110	VFG0184	2.00E-52	509	50	71	28	(gi:15598301) xcpQ - general secretion pathway protein D [Pseudomonas aeruginosa PAO1] (VF0084)
ABLAC_35140	VFG0710	5.00E-06	98	33	53	95	(gi:2865271) ler - Orf1 [Escherichia coli E2348/69] (VF0189)
ABLAC_35200	VFG0119	1.00E-53	514	43	64	101	(gi:15600454) algR - alginate biosynthesis regulatory protein AlgR [Pseudomonas aeruginosa PAO1] (VF0091)
ABLAC_35210	VFG0120	7.00E-46	449	34	55	81	(gi:15600455) algZ - sigma factor AlgU [Pseudomonas aeruginosa PAO1] (VF0091)
ABLAC_35250	VFG1441	3.00E-05	99	21	41	81	(gi:4835717) ibeB - IbeB [Escherichia coli] (VF0237)
ABLAC_35370	VFG1859	1.00E-67	639	28	44	116	(gi:52842863) feoB - ferrous iron transporter B [Legionella pneumophila Philadelphia 1] (VF0160)
ABLAC_35390	VFG1028	9.00E-16	188	59	67	21	(gi:15808708) intI1 - Tn21 integrase IntI1 [Shigella flexneri (serotype 2a) YSH6000]
ABLAC_35440	VFG1889	2.00E-65	615	55	76	99	(gi:52842852) letA - response regulator GacA [Legionella pneumophila Philadelphia 1] (VF0262)
ABLAC_35450	VFG1213	3.00E-34	349	36	61	41	(gi:15599742) pilS - two-component sensor PilS [Pseudomonas aeruginosa PAO1] (VF0082)
ABLAC_35460	VFG1214	1.00E-129	1166	50	70	94	(gi:15599743) pilR - two-component response regulator PilR [Pseudomonas aeruginosa PAO1] (VF0082)
ABLAC_35650	VFG1109	1.00E-20	231	24	45	95	(gi:15641779) nanA - N-acetylneuraminate lyase, putative [Vibrio cholerae N16961]
ABLAC_35710	VFG0168	7.00E-36	365	27	47	63	(gi:15599424) pchD - pyochelin biosynthesis protein PchD [Pseudomonas aeruginosa PAO1] (VF0095)
ABLAC_35840	VFG1050	3.00E-06	108	21	39	58	(gi:15808730) orf34 - unknown [Shigella flexneri (serotype 2a) YSH6000]
ABLAC_35850	VFG0586	5.00E-10	135	32	51	88	(gi:16767516) soxR - redox-sensing transcriptional activator SoxR, contains iron-sulfur center for redox-sensing (MerR family) [Salmonella enterica (serovar typhimurium) LT2]
ABLAC_36090	VFG0390	1.00E-32	335	27	44	91	(gi:16082728) yscN - putative Yops secretion ATP synthase [Yersinia pestis CO92] (VF0140)
ABLAC_36110	VFG0048	1.00E-30	319	26	43	75	(gi:33593244) bscN - putative ATP synthase in type III secretion system [Bordetella pertussis Tohama I] (VF0035)
ABLAC_36180	VFG1330	3.00E-07	115	23	40	76	(gi:22537388) lmb - laminin-binding surface protein [Streptococcus agalactiae 2603V/R] (VF0275)
ABLAC_36200	VFG0526	1.00E-18	213	27	48	85	(gi:16766168) sitB - Salmonella iron transporter: fur regulated [Salmonella enterica (serovar typhimurium) LT2]
ABLAC_36210	VFG1497	4.00E-06	104	35	47	50	(gi:24528027) ORF46 - putative ABC transporter membrane protein [Escherichia coli 536]
ABLAC_36340	VFG1026	3.00E-33	339	38	55	78	(gi:15808706) aadA1 - streptomycin adenyltransferase AadA1 [Shigella flexneri (serotype 2a) YSH6000]
ABLAC_36410	VFG1298	9.00E-06	102	25	41	54	(gi:21281854) cap8B - capsular polysaccharide synthesis enzyme Cap8B [Staphylococcus aureus MW2] (VF0003)
ABLAC_36480	VFG1821	2.00E-24	266	23	42	94	(gi:15609517) mbtE - mbtE [Mycobacterium tuberculosis H37Rv] (VF0299)
ABLAC_36500	VFG0082	8.00E-49	476	30	50	92	(gi:15640836) aldA - aldehyde dehydrogenase [Vibrio cholerae N16961]
ABLAC_36510	VFG1023	2.00E-05	99	32	47	29	(gi:21450882) orf7 - putative transcriptional regulator [Shigella flexneri (serotype 2a) YSH6000]
ABLAC_36590	VFG0164	1.00E-26	286	23	41	101	(gi:15599417) fptA - Fe(III)-pyochelin receptor precursor [Pseudomonas aeruginosa PAO1] (VF0095)
ABLAC_36680	VFG2077	5.00E-33	342	23	40	69	(gi:15595289) vgrG1 - hypothetical protein [Pseudomonas aeruginosa PAO1] (VF0334)
ABLAC_36770	VFG1381	6.00E-12	155	23	38	99	(gi:15607608) icl/aceA - aceA [Mycobacterium tuberculosis H37Rv] (VF0253)
ABLAC_36790	VFG1965	4.00E-06	106	22	41	64	(gi:15792755) Cj1437c - putative aminotransferase [Campylobacter jejuni NCTC 11168] (VF0323)
ABLAC_36810	VFG1472	1.00E-41	412	35	52	95	(gi:24528004) ORF21 - putative FMN-dependent dehydrogenase [Escherichia coli 536]
ABLAC_36840	VFG2362	1.00E-168	1508	61	78	96	(gi:123443280) manB - phosphomannomutase [Yersinia enterocolitica 8081] (VF0392)
ABLAC_36850	VFG2361	3.00E-97	892	51	65	99	(gi:123443279) galE - UDP-glucose 4-epimerase [Yersinia enterocolitica 8081] (VF0392)
ABLAC_36870	VFG1969	1.00E-15	188	22	45	71	(gi:15792759) kfiD - putative UDP-glucose 6-dehydrogenase [Campylobacter jejuni NCTC 11168] (VF0323)
ABLAC_36880	VFG0964	5.00E-60	570	41	63	99	(gi:15675935) hasC - UDP-glucose pyrophosphorylase [Streptococcus pyogenes SF370] (VF0244)
ABLAC_36890	VFG1309	5.00E-35	353	40	62	89	(gi:21281865) cap8M - capsular polysaccharide synthesis enzyme Cap8M [Staphylococcus aureus MW2] (VF0003)

ABLAC_36910	VFG1310	5.00E-10	137	28	48	90	(gi:21281866) cap8N - capsular polysaccharide synthesis enzyme Cap8N [Staphylococcus aureus MW2] (VF0003)
ABLAC_36930	VFG1308	2.00E-20	230	26	50	61	(gi:21281864) cap8L - capsular polysaccharide synthesis enzyme Cap8L [Staphylococcus aureus MW2] (VF0003)
ABLAC_36940	VFG1303	1.00E-119	1078	53	71	99	(gi:21281859) cap8G - capsular polysaccharide synthesis enzyme Cap8G [Staphylococcus aureus MW2] (VF0003)
ABLAC_36950	VFG1302	1.00E-89	827	42	62	100	(gi:21281858) cap8F - capsular polysaccharide synthesis enzyme Cap8F [Staphylococcus aureus MW2] (VF0003)
ABLAC_36960	VFG1301	1.00E-138	1248	69	84	96	(gi:21281857) cap8E - capsular polysaccharide synthesis enzyme Cap8E [Staphylococcus aureus MW2] (VF0003)
ABLAC_37000	VFG1922	2.00E-44	436	36	58	100	(gi:15792655) ptmA - putative oxidoreductase (flagellin modification) [Campylobacter jejuni NCTC 11168] (VF0114)
ABLAC_37010	VFG1921	2.00E-30	313	33	52	96	(gi:15792654) ptmB - acylneuraminate cytidylyltransferase (flagellin modification) [Campylobacter jejuni NCTC 11168] (VF0114)
ABLAC_37020	VFG0038	2.00E-08	126	21	42	73	(gi:33591351) bplA - probable oxidoreductase [Bordetella pertussis Tohama I] (VF0033)
ABLAC_37030	VFG1951	5.00E-22	243	33	50	65	(gi:15792741) Cj1423c - putative sugar-phosphate nucleotidyltransferase [Campylobacter jejuni NCTC 11168] (VF0323)
ABLAC_37040	VFG1336	2.00E-10	141	25	48	100	(gi:22537317) neuD - neuD protein [Streptococcus agalactiae 2603V/R] (VF0274)
ABLAC_37050	VFG1338	1.00E-67	637	37	57	97	(gi:22537319) neuB - N-acetyl neuramic acid synthetase NeuB [Streptococcus agalactiae 2603V/R] (VF0274)
ABLAC_37060	VFG1337	6.00E-69	648	37	56	101	(gi:22537318) neuC - UDP-N-acetylglucosamine-2-epimerase NeuC [Streptococcus agalactiae 2603V/R] (VF0274)
ABLAC_37070	VFG2226	2.00E-55	532	32	53	93	(gi:17987697) per - perosamine synthetase [Brucella melitensis 16M] (VF0367)
ABLAC_37080	VFG0029	2.00E-24	265	28	48	69	(gi:33591342) bplL - lipopolysaccharide biosynthesis protein [Bordetella pertussis Tohama I] (VF0033)
ABLAC_37090	VFG0431	1.00E-170	1525	66	82	99	(gi:16763123) tviB - Vi polysaccharide biosynthesis protein, UDP-glucose/GDP-mannose dehydrogenase [Salmonella enterica (serovar typhi) CT18] (VF0101)
ABLAC_37100	VFG0699	2.00E-17	204	23	40	98	(gi:30962647) bexD - BexD [Haemophilus influenzae 1007] (VF0043)
ABLAC_37120	VFG1298	1.00E-30	320	35	57	25	(gi:21281854) cap8B - capsular polysaccharide synthesis enzyme Cap8B [Staphylococcus aureus MW2] (VF0003)
ABLAC_37140	VFG1864	1.00E-25	266	52	64	100	(gi:52841028) mip - macrophage infectivity potentiator (Mip) [Legionella pneumophila Philadelphia 1] (VF0153)
ABLAC_37150	VFG1864	2.00E-37	373	36	51	98	(gi:52841028) mip - macrophage infectivity potentiator (Mip) [Legionella pneumophila Philadelphia 1] (VF0153)
ABLAC_37200	VFG0157	5.00E-22	241	35	49	92	(gi:15596041) plcH - hemolytic phospholipase C precursor [Pseudomonas aeruginosa PAO1] (VF0092)
ABLAC_37210	VFG0157	2.00E-99	913	40	56	104	(gi:15596041) plcH - hemolytic phospholipase C precursor [Pseudomonas aeruginosa PAO1] (VF0092)
ABLAC_37310	VFG0700	1.00E-10	143	26	48	62	(gi:30962648) bsc1 - bifunctional; ribulose 5-phosphate reductase; CDP-ribitol pyrophosphorylase [Haemophilus influenzae 1007] (VF0043)
ABLAC_37390	VFG1206	9.00E-32	326	36	52	82	(gi:15677997) fbpC - iron(III) ABC transporter, ATP-binding protein [Neisseria meningitidis MC58 (serogroup B)] (VF0272)
ABLAC_37400	VFG1474	5.00E-06	103	36	61	27	(gi:24528006) ORF23 - putative UidR transriptional regulator [Escherichia coli 536]
ABLAC_37460	VFG2161	2.00E-09	130	28	48	73	(gi:16803884) lspA - signal peptidase II [Listeria monocytogenes (serovar 1/2a) EGD-e] (VF0351)
ABLAC_37520	VFG1415	4.00E-12	158	25	41	65	(gi:15610623) lipF - lipF [Mycobacterium tuberculosis H37Rv] (VF0307)
ABLAC_37560	VFG1584	3.00E-09	132	23	39	90	(gi:23954267) orf50 - hypothetical protein [Escherichia coli 536]
ABLAC_37840	VFG1674	1.00E-08	129	27	43	55	(gi:28316254) orf51 - hypothetical protein [Escherichia coli 536]

Supplementary Table S4. MLST (Pasteur) sequence types generated by using genome FASTA sequences.

Strain	GenBank Accession No.	MLST (Pasteur) Allelic Profile							MLST-ST
		cpn60	*fusA*	*gltA*	*pyrG*	*recA*	*rplB*	*rpoB*	
Acinetobacter baumannii 1656-2	NC_017162.1	2	2	2	2	2	2	2	2
Acinetobacter baumannii AB030	CP009257.1	26	2	2	2	29	4	5	79
Acinetobacter baumannii AB031	CP009256.1	100	3	14	1	7	1	4	638
Acinetobacter baumannii AB0057	NC_011586.1	1	1	1	1	5	1	1	1
Acinetobacter baumannii AB307-0294	NC_011595.1	1	1	1	1	5	1	1	1
Acinetobacter baumannii AbH12O-A2	CP009534.1	26	2	2	2	29	4	5	79
Acinetobacter baumannii AC29	CP007535.1	2	2	2	2	2	2	2	2
Acinetobacter baumannii ACICU	NC_010611.1	2	2	2	2	2	2	2	2
Acinetobacter baumannii ATCC 17978	NC_009085.1	3	2	2	2	30	4	28	437#
Acinetobacter baumannii AYE	NC_010410.1	1	1	1	1	5	1	1	1
Acinetobacter baumannii BJAB07104	NC_021726.1	2	2	2	2	2	2	2	2
Acinetobacter baumannii BJAB0715	NC_021733.1	1	3	10	1	4	4	4	23
Acinetobacter baumannii BJAB0868	NC_021729.1	2	2	2	2	2	2	2	2
Acinetobacter baumannii D1279779	NC_020547.2	12	37	2	2	3	2	14	267
Acinetobacter baumannii MDR-TJ	NC_017847.1	2	2	2	2	2	2	2	2
Acinetobacter baumannii MDR-ZJ06	NC_017171.1	2	2	2	2	2	2	2	2*
Acinetobacter baumannii SDF	NC_010400.1	3	29	30	1	9	1	4	17
Acinetobacter baumannii TCDC-AB0715	NC_017387.1	2	2	2	2	2	2	2	2
Acinetobacter baumannii TYTH-1	NC_018706.1	2	2	2	2	2	2	2	2
Acinetobacter baumannii ZW85-1	NC_023028.1	3	3	2	2	11	57	4	639
Acinetobacter baumannii LAC-4	CP007712	1	3	2	1	4	4	4	10

ST assigned using genome sequence (ST437) is different from reported by Diancourt et al 2010 [3].

* The authors (Zhou et al 2011) reported MLST (Oxford) ST90 [4].

Table S5. Primer pairs used to amplify individual genes of the *hemO* cluster from *A. baumannii* strains.

hemO cluster locus*	Primer name	Sequence (5' – 3')	PCR fragment size[a] (bp)
873	ACICU_873-For	GGCTTCACCAATGGTTGAGAC	342
	ACICU_873-Rev	GGTAATGCAAAATAAAAGCTTGCTG	
874	ACICU_874-For	TGGATTGTTCAACTGAGTTCGG	854
	ACICU_874-Rev	CATTGACCCGCACATGTTG	
875	ACICU_875-For	GTGATAACTCTCATTTTTCGGGC	389
	ACICU_875-Rev	AAGGCCACTGCGGAACA	
876	ACICU_876-For	TGCTTGTTCAACTGCATTTGC	712
	ACICU_876-Rev	CTGGGCAATACCCGCTAAAG	
877	ACICU_877-For	ATATGCCGACGAAGACACACAG	1314
	ACICU_877-Rev	GGCATTTGCACAACAAAGGTAT	
878	ACICU_878-For	GTTGCCTTTACCACAAGGGC	865
	ACICU_878-Rev	GATTGAGTTTGAGTTGGACTGGAA	
879	ACICU_879-For	AAACAAGAAACTGCTGCTGAACA	240
	ACICU_879-Rev	CTGCCCGTTGGGTTGAA	
880	ACICU_880-For	ATGTCATGGTAAAACATGAGCAGTC	412
	ACICU_880-Rev	TTTAAGCCACATCCACGCC	

* *hemO* cluster genes are based on the locus IDs of strain ACICU [2], using only the last three digit of the locus ID; e.g., ACICU_00879 is abbreviated as 879.

[a] The estimated amplicon size is predicted based on the genome sequence of *A. baumannii* strain ACICU [2].

References:

1. Xu, Z., & Hao, B. CVTree update: a newly designed phylogenetic study platform using composition vectors and whole genomes. *Nucleic Acids Res*. **37**(Web Server issue): W174-W178 (2009).
2. Iacono, M. et al. Whole-genome pyrosequencing of an epidemic multidrug-resistant Acinetobacter baumannii strain belonging to the European clone II group. *Antimicrob Agents Chemother* **52**, 2616-25 (2008).
3. Diancourt, L., Passet, V., Nemec, A., Dijkshoorn, L. & Brisse, S. The population structure of Acinetobacter baumannii: expanding multiresistant clones from an ancestral susceptible genetic pool. *PLoS One* **5**, e10034 (2010).
4. Zhou, H. et al. Genomic analysis of the multidrug-resistant Acinetobacter baumannii strain MDR-ZJ06 widely spread in China. *Antimicrob Agents Chemother* **55**, 4506-12 (2011).
